# Evidence for a comprehensive approach to Aboriginal tobacco control to maintain the decline in smoking: an overview of reviews among Indigenous peoples

**DOI:** 10.1186/s13643-017-0520-9

**Published:** 2017-07-10

**Authors:** Catherine Chamberlain, Susan Perlen, Sue Brennan, Lucie Rychetnik, David Thomas, Raglan Maddox, Noore Alam, Emily Banks, Andrew Wilson, Sandra Eades

**Affiliations:** 1 Aboriginal Health Domain, Baker IDI Heart and Diabetes Institute, Level 4, 99 Commercial Rd, Melbourne, VIC 3004 Australia; 2 School of Public Health and Preventive Medicine, Monash University, Melbourne, VIC 3004 Australia; 3 Judith Lumley Centre, La Trobe University, 217 Franklin St, Melbourne, VIC 3000 Australia; 4 Healthy Mothers, Healthy Families Group, Murdoch Children’s Research Institute, Royal Children’s Hospital, Flemington Road, Parkville, VIC 3052 Australia; 5 School of Medicine, University of Notre Dame, 160 Oxford St, Darlinghurst, NSW 2010 Australia; 6 The Australian Prevention Partnership Centre, 13/235 Jones St, Ultimo, NSW 2007 Australia; 7 Tobacco Control Research, Menzies School of Health Research, PO Box 41096, Casuarina, NT 0811 Australia; 8 Well Living House, Centre for Urban Health Solutions, Li Ka Shing Knowledge Institute, St Michael’s Hospital, 209 Victoria St, Toronto, Canada; 9 Faculty of Health, University of Canberra, University Dr, Bruce, Canberra, ACT 2617 Australia; 10 Cancer Epidemiology Unit, University of Oxford, Oxford, UK; 11 Prevention Division, Department of Health, Queensland Government, 15 Butterfield St, Herston, QLD 4006 Australia; 12 National Centre for Epidemiology and Population Health, Australian National University, Mills Road, Canberra, ACT 2601 Australia; 13 Menzies Centre for Health Policy, University of Sydney, Camperdown, NSW 2006 Australia

**Keywords:** Indigenous, Aboriginal, Smoking, Tobacco, Overview, Systematic review, Framework

## Abstract

**Background:**

Tobacco smoking is a leading cause of disease and premature mortality among Aboriginal and Torres Strait Islander (Indigenous) Australians. While the daily smoking prevalence among Indigenous Australians has declined significantly from 49% in 2001, it remains about three times higher than that of non-Indigenous Australians (39 and 14%, respectively, for age ≥15 years in 2014–15). This overview of systematic reviews aimed to synthesise evidence about reducing tobacco consumption among Indigenous peoples using a comprehensive framework for Indigenous tobacco control in Australia comprised of the National Tobacco Strategy (NTS) and National Aboriginal and Torres Strait Islander Health Plan (NATSIHP) principles and priorities.

**Methods:**

MEDLINE, EMBASE, systematic review and Indigenous health databases were searched (2000 to Jan 2016) for reviews examining the effects of tobacco control interventions among Indigenous peoples. Two reviewers independently screened reviews, extracted data, and assessed review quality using Assessing the Methodological Quality of Systematic Reviews. Data were synthesised narratively by framework domain. Reporting followed the PRISMA statement.

**Results:**

Twenty-one reviews of varying quality were included. There was generally limited Indigenous-specific evidence of effective interventions for reducing smoking; however, many reviewers recommended multifaceted interventions which incorporate Indigenous leadership, partnership and engagement and cultural tailoring. Under the NTS priority areas, reviewers reported evidence for brief smoking cessation interventions and pharmacological support, mass media campaigns (on knowledge and attitudes) and reducing affordability and regulation of tobacco sales. Aspects of intervention implementation related to the NATSIHP domains were less well described and evidence was limited; however, reviewers suggested that cultural tailoring, holistic approaches and building workforce capacity were important strategies to address barriers. There was limited evidence regarding social media and mobile applications, for Indigenous youth, pregnant women and prisoners, and no evidence regarding interventions to protect communities from industry interference, the use of electronic cigarettes, interventions for people experiencing mental illness, juvenile justice, linguistic diversity or ‘pubs, clubs and restaurants’.

**Conclusions:**

There is limited Indigenous-specific evidence for most tobacco interventions. A ‘comprehensive approach’ incorporating NTS and NATSIHP Principles and Priorities of partnership and engagement, evidence from other settings, programme logic and responsive evaluation plans may improve intervention acceptability, effectiveness and implementation and mitigate risks of adapting tobacco evidence for Indigenous Australians.

**Electronic supplementary material:**

The online version of this article (doi:10.1186/s13643-017-0520-9) contains supplementary material, which is available to authorized users.

## Background

### Tobacco smoking and health inequities

Worldwide, 5.4 million people die every year due to tobacco use [1]. Tobacco smoking is one of the top preventable risk factors that influence the burden of disease among both Indigenous [2] and non-Indigenous [3] people in Australia. The prevalence of smoking in Australia is among the lowest in the world [4], with 14.5% of adults reporting smoking daily in 2014–15 [5]. However, while smoking rates have declined among Indigenous people in Australia (from 49% in 2002 to 39% among those aged 15 years and older in 2014–15) smoking rates remain about three times higher compared to the general population [5, 6]. These disparities between Indigenous and non-Indigenous peoples are similar in other high-income countries such as Canada, New Zealand, and the USA [7].

In Australia, the average life expectancy of Indigenous people born in 2010–2012 is approximately 10.6 years lower than that of non-Indigenous people [8]. These disparities are frequently cited as the worst among Indigenous and non-Indigenous peoples in similar high-income countries (Canada, New Zealand, and the USA) [9], and improving health equity for Indigenous people is a national priority in Australia [10]. Tobacco smoking was the single largest risk factor accounting for approximately 12% of the total burden of disease for Indigenous Australians and 23% of the ‘health gap’ in 2011 [2]. Thus, sustaining the decline in tobacco smoking is critical to improving healthy equity between Indigenous and non-Indigenous Australians.

The Council of Australian Governments National Healthcare Agreement includes a target to halve the daily smoking prevalence among Indigenous Australians from the 2008 prevalence of 44.8%, by 2018 [11]. The *Tackling Indigenous Smoking* programme was launched to achieve this ambitious target. In 2012, the Commonwealth, state and territory Health Ministers endorsed the National Tobacco Strategy, which included reducing smoking rates among Indigenous Australians as a priority [12]. More recently, the *National Aboriginal and Torres Strait Islander Health Plan (NATSIHP)* [13] was developed with extensive consultation with Indigenous communities to guide efforts towards ‘closing the gap’; and reduction in smoking is a significant focus of the implementation plan [14].

### Overview rationale

This overview was conducted under the auspices of the Australian Prevention Partnership Centre and is the second stage of a four-part project described in detail elsewhere [15]. The first stage of the project developed a framework for guiding a ‘Comprehensive approach to Aboriginal and Torres Strait Islander tobacco control’ (CATs) [16]. The CATs Framework combines the ‘key priority areas’ from the National Tobacco Strategy (NTS) [12] (aligned with the World Health Organization Framework Convention on Tobacco Control) with the vision, principles and priorities of the National Aboriginal and Torres Strait Islander Health Plan (NATSIHP) [13] which were identified as important by Indigenous communities. The methods for developing this framework are available on request [16].

The aim of this second stage of the project was to synthesise systematic review evidence to capture what is known about reducing tobacco use among Indigenous peoples worldwide, contextualised by and considered against the components of the CATs Framework.

The research questions for this overview are as follows:

1. What interventions have been examined in reviews of tobacco control among Indigenous peoples?
2. Is the range of identified interventions comprehensive when mapped against the CATs Framework domains?
3. What are the main intervention outcomes reported under each of the CATs Framework domains?
4. What is the quality of reviews of tobacco control among Indigenous peoples?

## Methods

We used methods for conducting an overview of systematic reviews. This approach was taken because there is a proliferation of reviews in the field of tobacco control, and overview methods enabled us to examine the coverage and applicability of evidence from these reviews in relation to the CATs Framework. By using overview methods, we were also able to examine the quality and extent of overlap and discordance among existing reviews, in order to help decision-makers apply existing review evidence for Indigenous Australians and identify gaps in review activity. This overview was led by Indigenous researchers and guided by an advisory group of investigators and key stakeholders, which included Indigenous and non-Indigenous experts in tobacco control and review methods. We developed a review protocol a priori (not registered with PROSPERO but available on request). We followed the PRISMA statement for reporting systematic reviews when items were applicable to overviews of reviews (Additional file 1).

### Criteria for inclusion of reviews in this overview

#### Types of studies

Any review or systematic review of published, peer-reviewed and grey literature was potentially eligible for inclusion.

#### Characteristics of participants

The participants are Indigenous people from Australia, Canada, New Zealand, and the USA. Reviews focused on ‘disadvantaged’, ‘vulnerable’ and ‘special’ populations, but reviews which made no explicit mention of Indigenous people were not included in this overview. Our rationale was that while Indigenous people share some common characteristics with other disadvantaged people and are often grouped together, there are unique issues for Indigenous peoples, such as those associated with experiences of colonisation and dispossession from land and culture. We also checked these reviews for any studies among Indigenous people’s that might be additional to those already included within Indigenous-specific reviews. However, no new studies among Indigenous peoples were identified, and therefore, the value of including these reviews was low. The four countries were selected as they are high-income countries with demonstrated success in tobacco control and the Indigenous peoples share similar histories of colonisation and health inequities.

#### Types of interventions

Interventions to reduce smoking of commercial tobacco were the focus of this overview. Interventions to reduce traditional or ceremonial tobacco use [17] were not included. The interventions examined were categorised according to the following key priority areas of the NTS and principles and priorities of the NATSIHP (CATs Framework domains):

National Tobacco Strategy^1^:

1. Continue to reduce affordability of tobacco products
2. Protect public health policy including tobacco control policies, from tobacco industry interference
3. Consider further regulation of contents, product disclosure and supply of tobacco products and alternative nicotine delivery systems
4. Strengthen mass media campaigns
5. Provide greater access to a range of evidence-based cessation services to support smokers to quit
6. Reduce exceptions to smoke-free workplaces, public places and other settings
7. Eliminate remaining advertising, promotion and sponsorship of tobacco products
  National Aboriginal and Torres Strait Islander Health Plan^2^—Principles and Priorities:
  Principles:
8. Equality
9. Partnership
10. Engagement
11. Accountability
  Priorities: Health enablers^2^ and Whole of Life:
12. Social and emotional wellbeing
13. Cultural respect
14. Evidence-based practice
15. Human and community capability
16. Whole of life (parents, children, adolescents, adults, ageing)

#### Types of outcome measures

Because this was a broad overview, aiming to map the type and amount of available evidence, our outcome eligibility criteria were deliberately inclusive. We included measures of the following primary and secondary outcomes irrespective of the outcome definition, measurement method or follow-up time specified by review authors.

The primary outcomes are as follows:

- Smoking cessation
- Prevention of initiation
- Prevalence reduction
- Tobacco sales reduction
- Morbidity/mortality

The secondary outcomes are as follows:

- Relapse prevention
- Quit attempts
- Smoke-free homes/workplaces
- Cost-effectiveness/cost
- Change in knowledge/norms (people, service providers)
- Change in practice
- Human and community capability/workforce development
- Adverse effects
- Self-efficacy/empowerment/strengths
- Improvements in equality
- Partnership
- Engagement
- Cultural respect

While the criteria in the protocol was broad, we included some guidance on what to look for (Additional file 2). We then used the independent review process to refine consensus on whether the outcome measures reported were relevant to that outcome where there was uncertainty.

### Search methods for identification of reviews

#### Electronic searches

Bibliographic databases, collections of systematic reviews and websites of institutes and organisations dedicated to Indigenous Health were searched (1 January 2000 until 31 January 2016) for identification of potentially relevant reviews. These included MEDLINE, EMBASE, PubMed, Turning Research into Practice (TRIPs), Epistemonikos, Centre for Reviews and Dissemination (CRD), Google Scholar, ATSIhealth, PDQ evidence, Health*Info*Net and AIHW Closing the Gap clearinghouse using the MESH and string terms outlined in Additional file 3 (‘tobacco’ AND ‘indigenous’ AND ‘review’) and detailed in Additional file 4. Reviews published prior to 2000 were excluded as considerable developments in the tobacco control landscape since this time make it unlikely that reviews prior to this date would still be considered relevant by decision-makers. Results from each search engine were downloaded into an Endnote reference library and saved as separate groups. Duplicate studies across the combined groups were deleted.

### Data collection and synthesis

#### Selection of reviews

Two reviewers (CC/SP) independently screened titles and abstracts for potentially relevant reviews. The full texts of remaining reviews were independently screened by two reviewers (CC/SP) and selected if they met the inclusion criteria. Advice was sought from a third reviewer (SB) if there were disagreements about reviews for inclusion, and a decision was reached by consensus. A general principle of erring towards inclusion of reviews was adopted where there was uncertainty. Excluded reviews are listed in Additional file 5.

#### Data extraction and management

A data extraction tool was developed in Microsoft Excel. The tool was piloted by two reviewers (CC/SP) on two reviews and modified to include the following:

- General review information (author, search dates)
- Review scope and aims
- Included study characteristics (study design, number of reviews, population and setting)
- Intervention descriptions under each of the NTS key priority areas and NATSIHP principles.
- Outcomes
- Assessing methodological quality of systematic reviews (AMSTAR) assessment
- Summary of review conclusions

Data were extracted independently by two reviewers (CC/SP), and any discrepancies or uncertainties discussed with a third reviewer (SB). Data were extracted from reviews only, and no data were extracted from individual studies. A matrix detailing the included studies within each review was extracted by one reviewer (SB).

#### Assessment of risk of bias in included reviews

As with all research, the design, conduct and reporting of reviews may introduce biases that influence the review findings. Two reviewers (CC, SP) independently assessed the quality of the included review’s methodology using AMSTAR (Additional file 6) as follows, with checking from a third reviewer (SB). AMSTAR items were rated (yes, no, cannot answer, not applicable), a rationale for each decision recorded, and an overall judgement was made about whether there were important concerns about biases in the review process or the interpretation of the evidence (see Additional file 2, items 6–6.14 for coding guidance). We did not assess the quality of individual studies within each review; instead, we report the review author’s assessment of quality. Considering the quality of evidence *across* reviews would have required an approach such as GRADE. However, the extent of narrative synthesis and diversity of approaches to assessing bias/quality of the primary evidence made it infeasible to apply current GRADE guidance.

#### Data synthesis

Two reviewers (CC/SP) synthesised data narratively in text and tabular form under each of the following subheadings, with the matrix of studies within reviews collated by one reviewer (SB):

1. A summary of characteristics of reviews, including populations, objectives, key outcomes, conclusions and AMSTAR appraisals (Table 1).

**Table 1:** Characteristics of included reviews

Review ID (Search dates) Risk of bias	Review title	Indigenous population	Interventions	No. and type of included studies	Synthesis	Main outcomes reported (summary)	Summary of reviewer conclusions
Minichiello 2016 [32] (1980 to 2014) Moderate	‘Effective strategies to reduce commercial tobacco use in Indigenous communities globally: A systematic review’	All	Any	93 (73 interventions) Quantitative [56] Mixed method [25] Qualitative [12]	Mainly statements about statistical significance	Smoking cessation: Mostly increased quit rates (4 studies) Prevention of initiation: 2/4 studies reported sig. effect Prevalence reduction: No sig. change (3 studies) Tobacco sales reduction: Unclear/no sig. change (1 study). Smoke-free homes/workplaces: no sig. effect (8 studies) Knowledge: Mostly positive impact (8 studies) Engagement: Increased community interest	Increasing priority and readiness to tackle high rates of commercial tobacco use employing comprehensive (multiple activities, centring of Aboriginal leadership, long-term community investments) and tailored interventions (provision of culturally appropriate health materials and activities).
Carson 2014 [33] (Up to 15 Aug 2014) Low	‘Smoking cessation and tobacco prevention in Indigenous populations’	All	Any	91 Randomised controlled trials [10], controlled clinical trials [5], pre-post studies [10], government reports [53], and protocols [4]	Mainly qualitative statements	Smoking cessation: Reduced smoking levels at follow-up in 12/15 controlled trials. Prevention of initiation: Results for youth not clear (9 studies).	Recommend multifaceted programmes that concurrently address behavioural, psychological and biochemical sides of addiction, using culturally tailored resources for individual Indigenous population needs. Interventions with more components, and greater intensity, were more likely to be effective than those of shorter duration and lower intensity.
Johnston 2013 [21] 1980 to May 2012 Moderate	‘Reducing smoking among indigenous populations: new evidence from a review of trials’	All	Any (if reporting Indigenous and non-Indigenous outcomes to assess effect of cultural tailoring)	5 Randomised controlled trials and cluster RCT’s [5]	Mainly statements about statistical significance	Smoking cessation: No sig. effect for either Indigenous or non-Indigenous participants in 3/5 studies.	No significant difference between Indigenous and non-Indigenous populations for smoking cessation and suggest not all tobacco control interventions can/need to be culturally adapted. Promising evidence on effectiveness of behavioural interventions using mobile phone technology.
Carson 2012a [27] (Up to April 2011) Low	‘Interventions for smoking cessation in Indigenous populations’	All	Any	4 Randomised controlled trials and cluster RCT’s [2] Non-randomised [2]	Mainly pooled effect estimates from meta-analysis	Smoking cessation: Sig. effect (risk ratio 1.43, 95% CI 1.03 to 1.98, *p* = 0.032). Adverse effects: Insomnia, rash and other minor complications reported from NRT treatment (26% versus 9%), compared to placebo. Knowledge: No sig. difference in ‘readiness to quit’. Costs and mortality reported.	Review highlights lack of available evidence to assess effectiveness of smoking cessation interventions, despite recognised success in non-Indigenous populations. Limited but available evidence does show smoking cessation interventions specifically targeted at Indigenous populations can result in smoking abstinence.
Carson 2015 [36] (Unclear) High	‘Culturally tailored interventions for smoking cessation in indigenous populations: A Cochrane systematic review and meta-analysis’	All	Any (focus on cultural tailoring)	9 Randomised or non-randomised controlled trials [9]	Mainly pooled effect estimates from meta-analysis	Smoking cessation: Non-sig effect (risk ratio 1.43 (95% CI 0.96 to 2.14); *p* = 0.08, 7 studies).	Some evidence supports using culturally tailored smoking cessation interventions for Indigenous populations. Most effective interventions were multifaceted cognitive and behavioural, mixing several initiatives simultaneously with health professional participation
DiGiacomo 2011 [22] (1990-2010) Moderate	‘Smoking cessation in indigenous populations of Australia, New Zealand, Canada, and the United States: elements of effective interventions’	All	Smoking cessation	9 Randomised controlled trials and cluster RCT’s [1] Non-randomised [8]	Mainly qualitative and descriptive statements	Quit rates: Higher quit rates reported for bupropion vs. placebo. Prevalence reduction: Mixed results from 5 studies. Cultural considerations: Describes cultural tailoring and levels of community engagement. Workforce/organisation: Describes Indigenous workforce involvement, organisational support, and financial/transport assistance for clients. Self-determination/flexibility: Describes programme flexibility and availability. Partnerships and engagement: Discusses strategies to promote engagement and principles for establishing partnerships.	Few identified interventions tailored for Indigenous populations. Successful interventions featured integrated, flexible, community-based approaches that addressed known barriers/facilitators to quit smoking.
CADTH 2013 [38] (Jan 1 2003 to Jun 26 2013) Moderate	‘Indigenous Knowledge for Smoking Cessation: Benefits and Effectiveness	All	Indigenous knowledge for smoking cessation	1 Systematic review [1]	Mainly qualitative statements	No studies found in systematic review.	No evidence regarding Indigenous knowledge for smoking cessation was identified.
Gould 2013a [25] (Up to Oct 2011) Low	Should anti-tobacco media messages be culturally targeted for Indigenous populations? A systematic review and narrative synthesis’	All	Culturally tailored mass media campaigns	21 Randomised controlled trials [4] Non-randomised [4] Database analysis [1] Qualitative [6] Mixed methods [6]	Mainly qualitative and descriptive statements	Smoking cessation: Higher quit rates reported among intervention groups. Intention to smoke: Significant decrease in future intention to smoke. Knowledge: Variable impact on recall, knowledge and intentions to quit reported. Cultural respect: 12 studies measured cultural suitability and/or relevance and qualitative studies showed preference for culturally targeted messages. Believability and usability also reported (3 studies).	Indigenous people had good recall of generic anti-tobacco messages, but preferred culturally targeted messages. Maori possibly less responsive to holistic targeted campaigns than generic fear campaigns. Culturally targeted internet/mobile phone messages just as effective in American Indians/Maori as generic general population messages. Where culturally targeted messages trialled, campaigns shown to be effective regarding change of knowledge, attitudes and behaviour.
Passey 2013 [26] (Up to Dec 2012) High	‘How will we close the gap in smoking rates for pregnant Indigenous women’	All (pregnant women only)	Any	2 Randomised controlled trials and Cluster RCT’s [1] Non-randomised [1]	Mainly qualitative statements	Smoking cessation: No sig. effect. Relapse prevention: No sig. effect.	No evidence for effective interventions that support pregnant Indigenous Australian women to quit smoking.
Carson 2012b [34] (Up to Nov 2011) Low	‘Interventions for tobacco use prevention in Indigenous youth’	All (adolescents only)	Any (controlled trials only)	2 Randomised controlled trials [2] Controlled clinical trial [0]	Mainly effect estimates for single studies	Tobacco use: No sig. changes between intervention/control groups at final follow-up. Changes in attitudes towards drugs and self-esteem: No sig. differences. Changes in knowledge: Sig. increases in knowledge in intervention groups.	Conclusion cannot be derived about efficacy of tailored tobacco prevention initiatives for Indigenous youth. This review highlights lack of data and need for more research in this area.
Carson 2013 [28] (Unclear) High (abstract only)	‘Interventions for tobacco prevention in Indigenous youth: A Cochrane review and a narrative synthesis’	All (adolescents only)	Any (controlled trials only)	6 Randomized or non-randomized controlled clinical trials [6]	Mainly qualitative statements	Tobacco use: No evidence of change.	Review highlights lack of data for tobacco prevention initiatives tailored to Indigenous youth.
Ivers 2003 [23] (1980 to March 2001) Moderate	‘A review of tobacco interventions for Indigenous Australians’	Australian (includes reflection on evidence from other populations)	Any	4 Qualitative [3] Other [1]	Mainly qualitative statements	Prevention of initiation: Reduced consumption reported. Knowledge: Knowledge about tobacco increased (1 study). Practice change/human capability: Some practice changed after training health professionals in brief interventions (1 study).	Major lack of research/evaluation on tobacco interventions for Indigenous people.
Ivers 2011 [18] (Unclear) High	‘Anti-tobacco programmes for Aboriginal and Torres Strait Islander people’	Australian	Any	Unclear Randomised controlled trials and Cluster RCT’s, Non-randomised, and qualitative - unclear how many	Mainly qualitative statements	Smoking cessation: Reports ‘successful approaches’ as: health professionals providing brief quit advice with pharmacotherapy; training health professionals; Quit groups; and multicomponent anti-tobacco programmes. Prevention of initiation/prevalence reduction: Sig. increases in readiness to quit and knowledge of tobacco from multicomponent interventions. Consumption declined in community with most tobacco control activity. Smoke-free homes/workplaces: A workplace quit smoking programme was acceptable. No other programmes aimed at decreasing environmental smoke sufficiently evaluated. Self-efficacy: Many smokers quit ‘by themselves’, emphasising importance of self-efficacy. Equality: Presents differential effects of interventions. Partnership and engagement: Notes importance of partnerships with community health organisations, and that programme delivery is enhanced by community involvement, ongoing funding and coordination. Cultural respect: Culturally appropriate, non-coercive counselling approaches likely to be appropriate. Aboriginal people believed tobacco programmes must be locally based, include local content, involve Elders and significant community members in design/delivery, and have a broad community focus.	Suggest successful approaches include: health professionals providing brief quit advice and pharmacotherapy; cessation advice training for health professionals; Quit groups; and well-delivered multicomponent anti-tobacco programmes. Community health organisations play key role in tobacco control, mainly in delivery of brief interventions and prescribing nicotine replacement therapy/pharmacotherapies, promoting smoke-free environments in antenatal/early childhood programmes, and in quit groups’ coordination.
Ivers 2014 [19] (Unclear) High	‘Attachment Two: The NSW Strategic Framework for Aboriginal Tobacco Resistance and Control – Supporting evidence’	Australian	Any	Unclear Randomised controlled trials and Cluster RCT’s, Non-randomised, and qualitative - unclear how many	Mainly qualitative statements	Smoking cessation: Effect seen from brief advice combined with pharmacotherapy; a locally developed intensive tobacco intervention; free nicotine patches/brief advice; and a quit group. No effect seen in an intervention for pregnant Aboriginal women; or National Tobacco Campaign evaluation. Prevalence reduction: Tobacco price reduced prevalence and cigarette costs identified as one of the reasons for quitting smoking. Knowledge: High levels of awareness, increases in knowledge and recall seen several campaigns; with high proportions finding them believable/relevant and considering quitting or cutting down but few report accessing Quitline. Workforce/practice change: Few health workers/practitioners recommended Quitline, despite increases in health workers confidence to talk about smoking. Increased numbers of health workers reported giving advice about NRT, environmental tobacco smoke, and reducing tobacco use. Self-efficacy: Evidence suggests quitting unaided improves self- efficacy in quitting. Partnership and engagement: Suggests critical to success. Cultural respect: Preferred campaigns are specifically designed for Aboriginal people, locally based, include local content, involve elders and significant community members in design/delivery, and have a broad community focus. Brief advice preferred in culturally appropriate, supportive and non-coercive way.	Factors that are vital to tobacco resistance and control programmes success include: Aboriginal communities develop, deliver and evaluate programmes; comprehensive and multi-component; funding for sustainable programmes over the long term; prevent duplication of effort between communities, non-government organisations and government agencies by coordination and partnerships. Types of effective interventions in decreasing Aboriginal smoking include: health professionals providing brief quitting advice and pharmacotherapy; cessation advice training for Aboriginal health workers and health professionals; multi-component tobacco resistance and control programmes; Quit groups; and intensive advice on smoking cessation.
Power 2009 [35] (2001 to 2007 (update of Ivers 2001 [18])) Moderate	‘Tobacco interventions for Indigenous Australians: a review of current evidence’	Australian	Any	12 Non-randomised [10] Qualitative [2]	Mainly qualitative statements	Smoking cessation: Increased quit rates reported in several studies. Prevalence and tobacco sales reduction: Changes in prevalence and store compliance with sales restriction legislation reported, except where vendor machines available (1 study). Knowledge: knowledge changes reported (1 study). Human capability and practice change: Increased health worker confidence in brief intervention (1 study). Cultural respect: 1 study attributes success to creating a culturally safe space.	Individually targeted smoking cessation approaches (e.g. NRT and/or counselling) may be effective for Indigenous Australians. No evidence about interventions likely to be effective in encouraging more Indigenous Australians to access quit support strategies. Limited evidence about possible effective approaches in surmounting major social/cultural barriers to Indigenous smoking cessation.
Upton 2014 [20] (2004–2014) High	‘Tackling Indigenous Smoking and Healthy Lifestyle Programme Review: A rapid review of the literature’	Australian	Any	36 (27 interventions) Randomised controlled trials and cluster RCT’s, non-randomised studies, qualitative studies, systematic reviews, policy documents, and unpublished reports	Mainly qualitative statements	Smoking cessation: Some of the 7 studies showed increased quit rates, including from intensive counselling and NRT. Prevention of initiation: Increased student self-esteem; positive impact on students’ knowledge/attitudes and self-efficacy (3 studies). Prevalence reduction: Reduction in self-reported smoking prevalence, but only statistically significant in one site (1 study). Tobacco sales reduction: Compliance with legislation around selling tobacco to minors in Indigenous Australian communities more difficult to achieve and problems with ongoing monitoring in remote areas, especially if tobacco access via vending machines/independent traders (2 studies). Quit attempts: Smoke-free workplace policies encouraged Quit attempts. Knowledge: Changes in knowledge reported from several interventions. Two studies demonstrated a clear link between health messages/negative attitudes to smoking/increased promotion/maintenance of smoke-free areas at home and in broader Indigenous Australian community. Also part of multi-component interventions. Adverse effects: Concerns anti-tobacco campaigns may have led smokers to feel persecuted/more defensive; and reports smokers feel more knowledgeable about smoking impacts, but possible barrier to ongoing engagement with messaging as ‘know everything’. Human capability and practice change: Increased confidence (1 study) and reviewer recommends developing local capacity/local workforce; expanding the Indigenous Australian workforce and increasing its capacity to deliver effective care. Self-efficacy: Discussion of importance of holistic approach.	Smoking environment changed significantly over recent years, with mixed evidence about if this has led smokers to feel persecuted/more defensive. Clear link seen in two studies between health messages/negative attitudes to smoking, and greater promotion/maintenance of smoke-free areas at home and in broader Indigenous Australian community. Many motivations to quit, but no particular reason encouraged Indigenous Australian smokers to ‘choose’ to quit. Evidence shows multilevel tobacco control approaches likely more effective for smoking prevalence decrease in Indigenous Australian communities. Formal/informal policies to ensure smoke-free environments in local organisations/businesses can also be effective, but require active participation of community members to ensure local ownership. Evidence supports high intensity counselling and brief interventions and use of NRT. Limited evidence around: school based interventions, Quitlines and pricing increases.
Clifford 2011 [31] (Jan 1990 to Aug 2007) Moderate	‘Smoking, nutrition, alcohol and physical activity interventions targeting Indigenous Australians: rigorous evaluations and new directions needed’	Australian	Any	5 Non-randomised [5]	Mainly qualitative statements	Smoking cessation: Increased quit rates reported in 3/4 studies. Costs: reported for 1 study.	Reviewer suggests it is comparatively rare for evaluations to be methodologically rigorous. Findings consistent with previous reviews showing intervention studies seldom done in Indigenous health and tend to have small effects. Recommend development and implementation of evaluation designs be informed by building capacity of local Indigenous communities and their healthcare services to engage as equal partners in research process.
Brusse 2014 [24] (Unclear) Moderate	‘Social media and mobile apps for health promotion in Australian Indigenous populations: scoping review’	Australian	Social media and mobile applications	4 Randomised controlled trials [1] App/social media programmes [3]	Mainly qualitative statements	Smoking cessation: Increased cessation in intervention (28%) compared to control (13%) group: Intervention as effective in Maori as non-Maori. Knowledge: Reports information on web downloads.	Current evidence for effectiveness/health benefit of social media and mobile software interventions especially for Indigenous/other traditionally underserved populations is scant and mixed.
Gould 2013b [37] (Up to March 2011) Low	‘Knowledge and views about maternal tobacco smoking and barriers for cessation in Aboriginal and Torres Strait Islanders: A systematic review and meta-ethnography’	Australian (women only)	Knowledge, attitudes, beliefs and barriers around smoking and cessation.	7 Non-randomised [1] Qualitative [5] Mixed methods [1]	Mainly qualitative statements	Smoking cessation: ‘Quitting is hard’ (1 study). Attitudes, beliefs and knowledge detailed. Smoke free homes/workplaces: Importance of reducing harm, being a protector (2 studies). Human capability/workforce: Role of IHW’s and other health professionals is challenging. Cultural respect: Cultural appropriateness and ethics is an important consideration in Indigenous studies.	Reviewer suggests comprehensive approaches, considering environmental context, increase knowledge of smoking harms/cessation methods, and provide culturally targeted support. Long-term, broad approaches are needed to de-normalise smoking in Indigenous communities as social norms and stressors perpetuate tobacco use in pregnancy. There is lack of knowledge of smoking harms and inadequate salience of current antismoking messages for maternal smokers, as well as poor knowledge of, access to, and use of evidence-based treatments for smoking cessation in pregnancy.
Thompson 2011 [29] (Unclear) Moderate	‘A review of the barriers preventing Indigenous Health Workers delivering tobacco interventions to their communities’	Australian (health workers only)	Impact of smoking status on provision of tobacco information.	14 Non-randomised [3] Qualitative [8] Reviews [3]	Mainly qualitative statements	Smoking cessation: Reports 9% quit rate; Relapse related to stressful times in clients lives. Knowledge/practice/workforce capability: Degree smoking information delivered may depend on IHWs’ tobacco use. Non-smoking IHWs more likely than smokers to talk to community about smoking (1 study) and smoking was barrier to giving support and/or information to community (1 study). Overall outcome showed IHWs own smoking was a barrier to service provision, but was not conclusive in one study. Need for workforce development recommended in 8 publications. Specific recommendations included training, mostly of health staff in brief interventions.	Overall, literature suggests IHWs’ smoking status is a barrier, but poor quality of most studies weakens evidence for this conclusion. Literature review has shown a need for practical quit support to help IHWs who want to quit. Training may also help increase IHWs knowledge in supporting community members wanting to alter smoking behaviour.
Clifford 2009 [30] (Jan 1990 to Aug 2007) Moderate	‘Disseminating best-evidence healthcare to Indigenous healthcare settings and programmes in Australia: identifying the gaps’	Australian	Dissemination of ‘smoking, nutrition, alcohol and physical activity’ interventions.	2 Non-randomised [2]	Mainly qualitative statements.	No smoking-related outcomes reported.	Review shows dissemination strategies targeting uptake of evidence-based SNAP interventions by healthcare providers working in Indigenous healthcare settings are not widely implemented, and evaluation outcomes often not published in peer-review literature. Recommend need for effective partnerships between government and research agencies, health-care providers and Indigenous healthcare services to improve likelihood of dissemination strategies implemented in Indigenous healthcare settings are feasible, acceptable and effective.

See Additional file 6 for detailed AMSTAR ratings for each review
2. A summary of interventions reported in reviews against each of the NTS priority areas (Table 2).

**Table 2:** Summary of interventions against the National Tobacco Strategy priority areas

Review ID	NTS P1 Continue to reduce affordability of tobacco products	NTS P2 Protect public health policy including tobacco control policies, from tobacco industry interference	NTS P3 Consider further regulation of contents, product disclosure and supply of tobacco products	NTS P4 Strengthen mass media campaigns	NTS P5 Provide greater access to a range of evidence-based cessation services to support smokers to quit	NTS P6 Reduce exceptions to smoke-free workplaces, public places and other settings	NTS P7 Eliminate remaining advertising, promotion and sponsorship of tobacco products
Minichiello 2016 [32]	2	0	0	4	75	9	0
Carson 2014 [33]	0	0	0	53	25	3	0
Johnston 2013 [21]	0	0	0	0	5	0	
Carson 2012a [27]					4		
Carson 2015 [36]					9		
DiGiacomo 2011 [22]					9		
CADTH 2013					0		
Gould 2013a [25]			2	8	13		
Passey 2013 [26]					2		
Carson 2012b [34]	0	0	0	2	0	0	0
Carson 2013 [28]	0	0	0	6	0	0	0
Ivers 2003 [23]	0	0	0	2	1	1	0
Ivers 2011 [18]	1	0	0	2	7	1	0
Ivers 2014 [19]	1	0	0	3	5	0	0
Power 2009 [35]	0	0	1	1	10	0	0
Upton 2014 [20]	1	0	2	5	15	7	0
Clifford 2011 [31]				3	2		
Brusse 2014 [24]				0	4		
Gould 2013b [37]					7		
Thompson 2011 [29]				0	11	0	
Clifford 2009 [30]				0	2		

0 if reviewer looked for; otherwise, blank
3. A summary of interventions reported in reviews against each of the NATSIHP principles and priorities (Table 3).

**Table 3:** Summary of NATSIHP principles and enablers addressed within included reviews^a^

	NATSIHP P1 equality and human rights approach	NATSIHP P2 partnership	NATSIHP P3 engagement	NATSIHP P4 accountability	NATSIHP health enablers/social and emotional wellbeing	NATSIHP health enablers/cultural respect	NATSIHP health enablers/evidence-based	NATSIHP health enablers/human capability	NATSIHP whole of life approaches
Minichiello 2016 [32]		✓	✓	▬				✓	▬
Carson 2014 [33]				▬		▬	▬	▬	▬
Johnston 2013 [21]	✓	▬	▬			✓		▬	
Carson 2012a [27]	▬		▬	▬		▬	▬		▬
Carson 2015 [36]				▬		✓			
DiGiacomo 2011 [22]	▬	▬	▬	▬	▬	✓	▬	✓	
CADTH 2013						▬			
Gould 2013a [25]		▬	▬		▬	✓	▬		▬
Passey 2013 [26]			▬		▬	▬	▬	▬	
Carson 2012b [34]	▬	▬			▬	▬	▬		
Carson 2013 [28]						▬			
Ivers 2003 [23]	▬		▬	▬		▬	▬	▬	
Ivers 2011 [18]	▬	▬	▬	▬	▬	▬			▬
Ivers 2014 [19]	▬	▬	▬		▬	▬	▬	▬	
Power 2009 [35]						▬	▬	▬	
Upton 2014 [20]	▬	▬	▬	▬	▬	▬		▬	▬
Clifford 2011 [31]		✓	✓	▬		▬		▬	
Brusse 2014 [24]							▬		
Gould 2013b [37]	▬				▬	✓	▬	▬	▬
Thompson 2011 [29]		▬			▬		▬	✓	
Clifford 2009		▬					✓		

✓indicates reviews assessed for and found studies explicitly addressing this principle or priority

▬indicates the issue is mentioned in the review, but not systematically assessed and reported

^a^Priority of ‘Health system effectiveness and clinically appropriate care’ was not included

This overview aims to provide a summary of the coverage and main conclusions from review level evidence against the CATs Framework. Meta-analysis was not conducted, as it is unlikely that an overall estimate of effect would have been meaningful. Meta-analysis was also not feasible, as most included reviews did not report a sufficient level of analysis for individual strategies. The degree of overlap of studies between reviews was considered to identify where there was a risk of ‘double counting’ the number of interventions where the same studies were reported in different reviews. In the presentation of our overview findings, we included selected extracts from the included reviews, particularly where these represent the overall findings in relation to the priority areas. Where possible, we avoided repeating narrative reporting of extracts about the same studies where multiple reviewers reported these. We also clarified where reviewer recommendations or suggestions appeared to be based on evidence from studies within the review. Where the evidence for the reviewer recommendation appeared to be based on expertise rather than clearly derived study data presented within the review, we have specified this and used verbs such as ‘the reviewer asserted, suggested, or recommended’.

#### Determining coverage of primary research and extent of overlap across reviews

The extent of overlap of studies across reviews helps determine whether consistent findings across reviews can be expected, and identifies possible explanations for discrepant findings. One reviewer (SB) extracted the list of references for studies included in each review, and then tabulated these in a matrix to show the coverage of primary research and the extent of overlap across reviews (Additional file 7). Only references to included primary studies (i.e. not reviews) that involved Indigenous people or communities and focussed on tobacco control were included in the matrix. Where reviews did not provide a complete list of included studies [18–20], studies were identified from citations in the text of the review.

We listed all unique references in the matrix, irrespective of whether there were multiple references for the same programme, study or both. The name of the programme/policy or project (hereafter referred to as programme) to which each reference was linked was included in the matrix when this information was available in the review. In general, the study-level characteristics reported by reviews were too limited and inconsistent to enable us to match all references from the same study or to determine if results were duplicated across references (as may be the case, for example, if results from a grey literature report to government are subsequently published in several peer-reviewed papers). The final set of references in the matrix therefore includes multiple references for some studies, encompassing different components or stages of an evaluation (e.g. programme description, study protocol, trial results, process evaluation) or reporting for different audiences (e.g. commissioners, researchers).

## Results

**Fig. 1:**
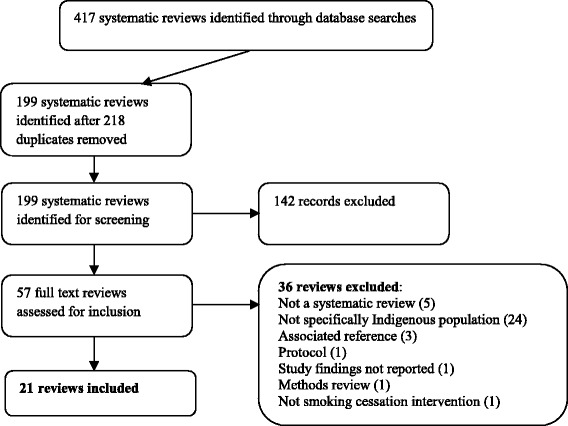
Flow chart of included reviews

Searches generated 417 possible reviews published between 2000 and February 2016. Following removal of duplications, 199 publications were screened using title and abstract and 142 were excluded, leaving 57 articles for full-text review. Of these, 36 were excluded because they did not specifically target Indigenous populations or they were not a review, and 21 reviews were retained. A list of excluded reviews and reasons are outlined in Additional file 5. A flow chart summarising search and screening results is provided in Fig. 1.

### Participants

The majority of reviews included studies involving Indigenous populations exclusively (*n* = 17). The remaining four studies also referred to evidence among general populations and/or included studies which compared outcomes between Indigenous and non-Indigenous people [21–24]. Specific sub-groups of populations that were reported as the focus of the reviews included pregnant women (*n* = 2) [25, 26], adolescents (*n* = 2) [27, 28], school students (*n* = 1) [28] and Aboriginal Health Workers (*n* = 1) [29]. Thirteen reviews included Indigenous people in any country, but only reported studies based in Australia, Canada, New Zealand, and the USA. Eight reviews were restricted to studies involving Indigenous people in Australia only.

### Interventions (review scope)

While reviews published after 2000 only were included, a number of studies within those reviews dated back to 1980. Eighteen reviews specifically focussed on tobacco programmes/interventions, while the remaining three encompassed a broader scope of health promotion [24] or ‘smoking, nutrition, alcohol, physical activity’ trials [30, 31] which included a tobacco programme component.

Four reviews aimed to evaluate all tobacco control programmes among Indigenous peoples [32–34], while five reviews assessed all interventions for Indigenous people in Australia [18–20, 23, 35]. Three reviews assessed the effect of culturally tailored interventions [21, 36, 37], and one review looked for evidence of Indigenous knowledge to support smoking cessation [38]. Two reviews included all tobacco control interventions to reduce smoking among Indigenous adolescents [27] and among Indigenous pregnant women [26]. Remaining reviews looked specifically at individual smoking cessation strategies among Indigenous people [22] evidence for social media and mobile apps among Indigenous Australians [24] smoking among Indigenous Health Workers [29] knowledge and attitudes of Indigenous Australian mothers to smoking [25] and methods [31] and dissemination [30] of ‘smoking, nutrition, alcohol and physical activity’ trials among Indigenous Australians.

### Types of studies included within reviews and comparisons

Approximately half of the included reviews (*n* = 11) included unrestricted study designs (qualitative, quantitative or reports) [18–20, 23–25, 29, 32, 33, 35, 37], and the remainder restricted the inclusion criteria to quantitative randomised controlled trials and controlled trials.

Comparisons for the interventions examined within included reviews were not well documented. Inclusion criteria for included reviews either did not specify a type of comparison (*n* = 8), specified ‘any’ comparison (*n* = 10), specified comparisons as ‘usual practice, placebo or none’ (*n* = 2) or there was no comparison or it was ‘not applicable’ (*n* = 1). In the descriptions of comparisons that were found by reviewers, these were either not reported (*n* = 12), ‘any’ type of comparison or unclear (*n* = 1), specified as ‘usual practice, placebo or none’ (*n* = 6) or ‘not applicable’ (*n* = 2).

### Outcomes

Smoking cessation outcomes were synthesised in a number of ways in the reviews. Only two reviews included meta-analysis [34, 36], one reported effect estimates of single studies [27], two reported statements of statistical significance [21, 32] and the remainder reported mainly qualitative statements, such as ‘increase’, or ‘no difference’ to describe smoking cessation outcomes.

Quit rate/smoking cessation was the main outcome that was considered in approximately half of the reviews (*n* = 10). Six reviews considered knowledge and attitudes and five reviews were non-specific, i.e. ‘any’ outcomes were considered. A summary of the main outcomes are outlined in Table 1 and incorporated into the narrative synthesis under each of the CATs Framework domains.

### Summary of reviewer conclusions

The majority of reviewers findings highlighted the importance of tobacco control in improving Indigenous health and recommended multifaceted interventions [18–20, 25, 32, 33, 36] or ‘multi-component policies’ [18, 19, 32, 33] which included Indigenous leadership/partnership/engagement [18–20, 22, 30, 32, 36] and cultural tailoring [25, 29, 32, 33, 36, 37] when appropriate [21]. Several reviews cited as evidence an evaluation of ‘multi-component tobacco control activities’ in six Australian communities in the Northern Territory, which showed a decrease in tobacco consumption in Indigenous communities [18], but the differences were not statistically significant and there was high variation. For example, a review by Ivers [19] concludes (p. 2):

Programs likely to have greatest success in reducing smoking in Aboriginal communities are multi-component that address different aspects of tobacco, take whole-of-community approach, integrated across different activities within health services, and work across different sectors within communities. In effective multi component tobacco control programs activities reinforce and strengthen each other. It is also important to ensure tobacco control programs are linked to range of other relevant health priorities identified by community and integrated with other chronic disease prevention initiatives.

These recommendations appeared to be based on reasoned arguments about effectiveness in other populations and rationale to improve acceptability and implementation of interventions, rather than clear evidence of effectiveness of these strategies among Indigenous peoples. The insufficiency of Indigenous-specific evidence was also highlighted [23, 30, 31], particularly for addressing social and cultural barriers to smoking cessation [35], social media and mobile applications [24], and interventions for adolescents [27, 28] and pregnant women [29].

#### Review quality

We assessed six reviews as low risk of bias, eight as moderate risk of bias and seven as high risk of bias using the AMSTAR rating (Additional file 6). The most common issues were lack of information about search strategies and results, duplicate data extraction and quality appraisals.

See Table 1 for a summary of characteristics of included reviews.

#### Coverage of primary research in included reviews

Additional file 6 presents the matrix of the 199 unique references to primary studies included in the 21 reviews within this overview. The matrix provides an overview of the amount of evaluative research on tobacco control strategies for Indigenous peoples included and examined in reviews. It also shows the extent to which reviews overlap in the studies they have identified and included in their synthesis. The full references for individual studies as per matrix are listed in Additional file 7.

References to studies involving Indigenous people in Australia are listed first (*n* = 121 references; composed of 41 peer-reviewed publications and 80 grey literature reports), sorted in alphabetical order of the programme name (see Additional file 7). The remaining 78 references (67 peer-reviewed publications; 11 grey literature reports) reported studies involving Indigenous peoples from the USA (*n* = 44), New Zealand (*n* = 19), Canada (*n* = 13), Fiji (*n* = 1) and Taiwan (*n* = 1).

We identified 116 named programmes from 148 references, of which 94 programmes were in Australia. For 51 references, we were unable to identify a programme or project name from review level data, so could not determine if these included multiple reports for the same programme or study (see Additional file 7).

#### Extent of overlap in the primary research included across reviews

The number of references to included studies per review ranged from 0 [39] (no eligible primary studies) to 93 reports of 73 interventions (median per review = 7). This variation across reviews was largely explained by differences in the scope of review questions, with narrower reviews restricting their inclusion criteria to specific sub-groups (e.g. location, age, women who are pregnant), strategies (e.g. prevention focussed, cessation focussed, culturally tailored) or study designs (e.g. randomised trials, qualitative studies, any design). Most reviews included at least one reference not included in any other review; exceptions being reviews updated by their author (i.e. reviews by Carson and Ivers).

Collectively, three broad reviews [20, 32, 33] included 170 of the 199 references listed in the matrix. Overlap in the references from these reviews was less than expected based on their inclusion criteria; 26 of the 170 references were included in two of the three reviews, and only six appeared in all three reviews. In part, this is explained by the inclusion in Carson [33] of reports describing programmes for which evaluation findings were not available or where an evaluation was ongoing (*n* = 57/91 references of which 53 were grey literature reports). Of the 34 references in Carson [33] that reported evaluation results, 20 were included in Minichiello [32] and seven in Upton [20]. Minichiello [32] included 60 references that were not included by Carson [33], among which were papers postdating Carson (*n* = 11), programmes for which Carson included a different reference (*n* = 6), and grey literature reports for Canadian programmes (a focus of Minichiello [32], *n* = 9). References unique to Upton [20] (*n* = 18) were mainly grey literature reports from Australian programmes.

#### Participant subpopulations within included studies

A range of Indigenous subpopulations was included in studies within the reviews. Recognising that there is overlap of the same studies within reviews as outlined above, we were able to identify included studies within the reviews among the following specific subpopulations:

- Pregnant women [19, 20, 24–26, 29, 33, 37]
- Women (views) [25]
- Families of smokers [32, 33]
- Adolescents and school students [18, 20, 21, 23, 27, 31–33, 35, 37]
- Prisoners [18, 33]
- People living in rural and remote locations [18–20, 31, 32, 35, 37]
- Health service clients [18, 19, 22, 25, 32–34, 37]
- Aboriginal health workers [19, 20, 25, 29, 32, 35]
- Other health professionals/general practitioners [18, 20, 23, 25, 32, 35, 37]
- Community organisations [20, 22, 31–33, 35]
- Tobacco retailers [20, 32, 35]
- Government (strategic multilevel approaches) [20]

We were unable to identify any reports of studies among juveniles within the justice system or Indigenous people experiencing mental illness, explicitly considering Indigenous people with low socio-economic status, linguistically diverse Indigenous people, or ‘pubs, clubs and restaurants’ in Indigenous communities. Male participants in study populations were not specifically mentioned in reviews but included in mixed populations of men and women. Descriptions of subpopulations in some reviews were unclear. For example, youths could be included in adolescent and/or school student populations. Several reviews did not describe specific subpopulations [30, 36, 38].

#### Interventions within included studies

Consistent with the inclusion criteria of this overview, most reviews included interventions to stop, reduce or prevent tobacco use (*n* = 18). Of the 18 reviews, nine included any type of tobacco control strategy [18–20, 23, 31–35], two included any tobacco control interventions to reduce adolescent smoking [28], one included any strategies reporting outcomes among Indigenous and non-Indigenous people to assess the impact of cultural tailoring [21] and one included any culturally tailored cessation strategies [36]. Three of the 18 tobacco control reviews focussed on smoking cessation, including one review of all cessation strategies [22], one review of smoking cessation among pregnant women [26] and one looked for reviews of Indigenous knowledge for smoking cessation tobacco use [38]. Two of the tobacco control strategy reviews assessed communication and mass media (and whether it should be culturally tailored) [37] and social media/mobile technology [24]. Three reviews differed in that they examined attitudes, beliefs and knowledge about maternal smoking and barriers to cessation [25], the smoking status of Indigenous health workers and impact on their provision of smoking cessation support [29] and types of smoking dissemination strategies [30].

### National tobacco strategy priority areas: summary of reported review evidence

Data were extracted from the reviews against the nine key priority areas of the NTS. The summary of findings is displayed in Table 2 for seven of the priority areas. In this overview, information related to two priority areas from the NTS (‘Strengthen efforts to reduce smoking among populations with high smoking prevalence’ and ‘Bolster and build on existing programmes and partnerships to reduce smoking rates of Aboriginal and Torres Strait Islander people’) have been incorporated into the NATSIHP principles of ‘Equality’ and ‘Partnership’.

Across the 21 included reviews, the most frequently’ identified interventions were coded under ‘providing greater access to a range of evidence-based cessation services’ (in 17 reviews) and ‘strengthening mass media campaigns’ (in 12 reviews). Eliminating advertising, promotion and sponsorship of tobacco products was the least looked for and least found priority (in five reviews). Recognising there is a degree of overlap of studies between reviews, the types of interventions found within each review are listed under respective NTS priority areas in Table 2 and summarised narratively below.

### Priority 1: continue to reduce affordability of tobacco products

Four reviews [18–20, 32] identified studies and information about reducing the affordability of tobacco products. The main strategies mentioned were tax increases and pricing changes. The reviewers may have looked for pricing studies in six reviews, but it appeared that reviewers did not search for pricing interventions in 11 of the reviews.

Minichiello [32] included two studies of tax increases and reported one Australian study of a 25% tax increase on commercial tobacco that showed no change in smoking or purchasing behaviour among community residents. However, Ivers [19] concluded there was ‘strong evidence for increased taxation’ to reduce tobacco-related harm, prompt quit attempts and reduce tobacco consumption including among the most disadvantaged in the community. The review suggests Indigenous Australians have identified the cost of cigarettes as an important reason for quitting smoking; however, a tobacco website is cited as the source [19]. In 2011, Ivers [18] had previously reported that the effect of taxation and pricing changes not been evaluated for Indigenous Australians but had the potential to decrease consumption. Ivers [23] also noted that increases in price of tobacco products may result in hardship for smokers who do not reduce consumption. Upton [20] concluded that there is some evidence increasing taxes can reduce smoking in Indigenous communities. However, this review reports market research of community concerns about the financial impact of price increases on people who are unable to quit, whether all smokers may be responsive to pricing changes, and that some smokers may change to cigarettes with a higher nicotine content or smoke more intensively. The ‘key message’ concluded from the review by Upton [20] concerning pricing measures was that (p. 7):

Tax rises on tobacco products are generally viewed positively, however, the impact of increases in tobacco pricing on smoking behaviours in this [Aboriginal] population is not yet clear. Combining national policies with access to quit support services may help increase the effect of these policies on individual quit rates.

### Priority 2: protect public health policy including tobacco control policies, from tobacco industry interference

There were no explicit reports of interventions to protect Indigenous communities from tobacco industry interference. Interventions related to protecting public health policy did not appear to be included in 11 reviews and appeared to be looked for and not found in a further 10 reviews.

### Priority 3: consider further regulation of contents, product disclosure and supply of tobacco products and alternative nicotine delivery systems

Three reviews [20, 35, 36] identified studies about pack warnings and restrictions to tobacco sales via local community stores or to minors. Reviewers did not appear to search for legislative interventions in 10 reviews, and it appeared that reviewers searched and did not find any studies or information in eight reviews. In 2001, Ivers [18] suggested a need for continued support and enforcement of federal and state tobacco legislation in Indigenous communities. All three reviews, which identified legislative studies, reported that most remote area stores comply with legislation for display of anti-tobacco advertising and restricting tobacco sales to minors, except where vending machines were located. Gould [25, 37] included studies which assessed pack warnings [40] and different tobacco control interventions and recommended that legislative interventions would be most effective if staff were trained in enforcing legislation and provision of quit smoking information at point-of-sale. Upton [20] identified two studies evaluating restrictions of tobacco sales to minors and reiterated that restrictions on sales to minors may be effective, but only if enforced by retailers, and commented this could be difficult to enforce in remote areas. Upton [20] also cites research suggesting that cigarettes among minors are often sourced from non-commercial areas and recommended that sales restrictions need to be combined with other strategies to reduce consumption, such as controls in schools and media campaigns [41]. However, these reviewer recommendations appear to be based on expertise and limited descriptive data rather than evidence of effectiveness.

### Priority 4: strengthen mass media campaigns

Interventions related to strengthening mass media campaigns were identified in 11 reviews [18–20, 23, 27, 28, 31–33, 35, 37]. Components of interventions included mass media campaigns, community-based strategies, social marketing and social media, often conducted as part of a multi-component strategy. There were four reviews that appeared to look for, but did not identify any studies or information, and another six reviews where it appeared this priority was not looked for.

Mass media and social marketing campaigns aim to change attitudes, beliefs and intentions surrounding tobacco use and subsequently change behaviours [20]. Evidence from the general population suggests mass media campaigns can help prevent smoking uptake and promote smoking cessation, particularly where campaigns are combined with other tobacco control activities [20]. This is achieved through supporting relapse prevention, encouraging calls to Quitlines and denormalising smoking among young people [20]. Reviewers concluded there is limited evidence on the effectiveness of mass media campaigns in reducing smoking rates among Indigenous Australians [18–20, 23, 32, 35] and limited evidence of reduced smoking uptake among adolescents [27, 28]. Two reviews [18, 20] highlighted ‘The Tobacco Project’ in the Northern Territory [42], which demonstrated a small but not statistically significant reduction in tobacco use of 1.2% at the end of the project across all communities, with substantial variation between communities. Upton [20] suggested ‘success factors’ on this project include: tobacco control being identified as a priority within the community, local strategy development and communities having a dedicated local workforce to deliver services. However, the reviewer noted that these activities were not sustained due to a lack of resources and smoking rates rose after the intervention ended [20].

Despite limited evidence of an impact on smoking rates among Indigenous people, there is evidence from studies within included reviews that mass media campaigns have a significant effect on recall, knowledge, attitudes and beliefs related to smoking [18–20, 23, 32, 35, 37]. This includes a review of studies among adolescents [27] which pooled results from two randomised studies of multi-component community-based interventions and found no significant changes in tobacco use, attitudes or self-esteem, but there were increases in knowledge. Another review [20] described two programmes for school students in Queensland (Australia), ‘Smokin’ No Way’ which demonstrated an increase in self-esteem and ‘Deadly choices’ which demonstrated an increase in self-efficacy, as well as knowledge. An impact on knowledge is also outlined in the ‘Talking about the Smokes’ project in Australia [20]. Gould [25] conducted a review of cultural tailoring for Indigenous peoples in mass media campaigns and found that culturally tailored messages were preferred and appeared to be similarly effective to non-tailored messages. Two reviews [18, 19] describe findings from the National Tobacco Campaign in Victoria and remote Northern Territory (Australia), where Indigenous respondents preferred a specifically designed campaign and believed tobacco programmes need to be locally based, include local content, involve Elders and significant community members in design and delivery, and have a broad community focus. A review of qualitative studies emphasised that for maternal smokers, personal stories were likely to be more ‘trusted’ than data and statistics [25].

Gould [25] reported findings from a study among Māori people in New Zealand suggesting that a mainstream mass media campaign with strong personal, but negative emotive messages, was more effective than a Māori-specific ‘strengths-based’ campaign. This contrasts with the promotion of strength-based messages in the NATSIHP. However, Upton [20] reported market research in Australia suggesting a perception that many smokers may have become immune to graphic imagery and shock and that there was growing resentment among some smokers about the use of guilt in tobacco advertising. Reviewers also commented that increasing knowledge about smoking could be a barrier to engagement with messaging as smokers ‘know everything already’ [20]. While social media and mobile application use is growing in popularity, including in Indigenous Australian communities, there was limited evidence from a review of these applications [24].

Upton ([20], p. 10) asserted that social marketing and mass media campaigns ‘can have a powerful impact on attitudes and beliefs about smoking, but messages need to have personal and cultural relevance to be effective. Changes in attitude brought about by social marketing can act as a precursor to behaviour change and this will be most effective where communities are actively driving tobacco control activities, and a local workforce is available to support individuals to quit.’

However, these reviewer conclusions were based on expertise and limited Indigenous-specific evidence of preferences and an impact on attitudes and knowledge rather than evidence of an impact on smoking prevalence from mass media campaigns.

### Priority 5: provide greater access to a range of evidence-based cessation services to support smokers to quit

Smoking cessation interventions were looked for in all reviews and found in 18 reviews [18–26, 29–37]. Reviews encompassed interventions that covered pharmacological components (e.g. nicotine replacement therapy (NRT), bupropion), different types of counselling, training, Quitlines, understanding attitudes of Indigenous Health Workers to smoking cessation, dissemination and text messaging. While there is overlap between studies, there are clearly more studies of smoking cessation interventions (75 in one review [32]) than interventions categorised under all other NTS priorities combined. No smoking cessation studies were identified in two reviews of interventions for adolescents [27, 28] and a review of Indigenous knowledge for smoking cessation [38].

Despite the comparatively large volume of research on smoking cessation, the evidence of effectiveness among Indigenous people is modest and comes from few rigorous studies. Only two reviews presented weighted pooled results of smoking cessation interventions. Carson [27] reported evidence from four studies suggesting Indigenous people receiving smoking cessation interventions were 43% more likely to quit than Indigenous people in the control group (risk ratio (RR) 1.43, 95% confidence interval (CI) 1.03 to 1.98). A similar point estimate, with confidence intervals overlapping with those of Carson [27] was found in another review of culturally tailored smoking cessation interventions [36] from seven studies (RR 1.43, 95% CI 0.96 to 2.14), indicating a consistent, but not statistically significant result. Although these randomised controlled trial designs are rigorous, the sample sizes are small with low statistical power [20]. Reported challenges recruiting adequate numbers of participants to trials also raises questions about the intervention acceptability and uptake when applied at a population level [20].

Other narrative reviews reported relatively consistent results. Most reviews reported studies with positive effects on smoking cessation [18–20, 22, 24, 31–33, 35, 37]. The effect was unclear in a review examining whether cultural tailoring increased the effectiveness of smoking cessation interventions [21] and two studies among pregnant women [26].

Most reviews concluded that multifaceted smoking cessation strategies where brief interventions and/or counselling were combined with pharmacological approaches were more effective that single interventions [18–20, 22, 33, 35, 36], but not to the same extent as among non-Indigenous populations [20]. For example, counselling and NRT trials among Indigenous people have demonstrated quit rates of 6–10%, compared with 15–19% among non-Indigenous people [20]. One review [18] reported positive results from a study of the use of bupropion for smoking cessation among prison inmates, 50% of whom were Indigenous Australians. One review reported adverse effects from pharmacological interventions [34], such as insomnia and rash.

Despite apparent evidence of effectiveness, several reviews highlighted reported barriers to implementation of brief interventions [30], including unease of health providers giving advice and ‘alienating’ clients and inappropriately telling them how to behave [20]. Thus, reviewers suggested cultural sensitivity was important in improving acceptability [20]. Perceived barriers were higher when health workers themselves were smokers [29]. Hence, supporting and training health workers and increasing confidence in delivering smoking cessation support was considered to be an important component of multifaceted interventions [19, 29]. Upton [20] reported barriers to provision and ‘compliance’ with pharmacological interventions in remote areas implementation in remote areas, including time taken for NRT supplies to arrive in remote areas, individuals running out of NRT patches because they share with other family members, and cost. But these barriers were overcome with local support. Ensuring effective partnerships to improve the feasibility and acceptability (and hence effectiveness) of dissemination strategies was also recommended in a review outlining poor implementation of ‘Smoking, Nutrition, Alcohol and Physical Activity (SNAP)’ interventions in Indigenous healthcare settings, although no Indigenous-specific evidence of effectiveness to support this recommendation was reported [30].

There was limited evidence reported in reviews about quit groups and quit lines [19, 20, 35], but reviewers raised questions about accessibility of these support services for Indigenous people in Australia [18]. Ivers [19] reported data from a study suggesting that few Aboriginal Health Workers referred to quit lines, despite broad awareness of them, and confidentiality coupled with ongoing after-hours support was perceived as a strength in another review [35]. The main barriers reported were resistance to talking to someone unknown and costs of mobile phone calls [19]. Recent efforts to increase acceptability of quit lines in a study in Victoria (Australia) were reported to increase calls in one review [20]; however, no studies on the impact of these initiatives on quitting among Indigenous peoples were cited. Ivers [19] also asserted that face-to-face and local-level quitting support was still going to be an important component of support for many people. Despite the increased use of mobile phone technology and social media within Indigenous communities, a review of these technologies [24] found only one evaluation study [39], which reported evidence demonstrating a text-messaging intervention was as effective for Maori as non-Maori for increasing smoking cessation. Brusse [24] also found descriptions of four projects with significant social media components and three mobile phone applications specifically targeting smoking cessation for Indigenous Australians. Two reviewers suggested these technologies look promising [21, 24], based on limited evidence as described.

The modest but consistent evidence reported from smoking cessation interventions illustrates the importance of smoking cessation being considered as part of a multifaceted strategy, and this has been concluded in numerous reviews [18–20, 22, 25, 29, 32, 33, 35, 36]. One review suggests not all interventions need to be culturally adapted, based on evidence demonstrating little difference in effectiveness [21]. However, based on qualitative evidence showing preferences for culturally tailored materials, the reviewer also suggested cultural adaptation may improve acceptability and utility of the interventions [18], including accessibility to Quitlines. As reported in a review of qualitative views of women [25], ‘quitting is hard’, prompting reviewers’ suggestions that Indigenous people need a supportive environment to support and maintain quitting attempts, particularly for pregnant women [19]. The importance of quitting unaided and supporting self-efficacy was also discussed [19]. Gaps in evidence for effective strategies for smoking cessation were highlighted, specifically among adolescents [27, 28] and pregnant women [26].

### Priority 6: reduce exceptions to smoke-free workplaces, public places and other settings

Interventions to promote smoke-free environments were identified in five reviews [18, 20, 23, 32, 33]. It appeared as though these interventions were searched for, but not identified, in six reviews.

A primary aim of smoke-free environments is to reduce second-hand smoke exposure, and reviews reported evidence from studies suggesting high levels of acceptability in Indigenous communities for smoke-free public buildings [18, 23], workplaces [35] and homes [20, 25]. Gould [37] suggested these strategies could be particularly important for families to capitalise on the desire to reduce harm and ‘be a protector’.

Combining smoke-free strategies with smoking cessation strategies was asserted as important in three reviews [20], including workplace quit support for health workers [29] and the need for ‘multifaceted interventions to support quitting’ [35]. However, there is limited evidence about the effect of smoke-free environments (workplaces and homes) among Indigenous people [18, 23, 33] on quitting. In a review by Minichiello [32], none of the eight quantitative studies of smoke-free interventions reported a significant difference in tobacco use.

One review suggested that smoke-free regulations may be less likely to be strictly enforced in rural and remote areas in Australia [35]. Upton [20] described studies in remote areas in the Northern Territory and Queensland (Australia) where smoke-free communal spaces have been successfully maintained and changes in attitudes to smoking were reported. However, Upton [20] reported study data suggesting there were some negative effects on smokers feeling alienated by these policies, and ‘pushed away’ from non-smokers, and questioned whether smoke-free interventions may inadvertently increase solidarity between smokers as a group and create new barriers. Upton [20] also recommended that ‘genuine’ community participation, ownership and local leadership was integral to the success of smoke-free environments, particularly in remote areas, but it appeared that this recommendation was based on expertise rather than research evidence.

### Priority 7: eliminate remaining advertising, promotion and sponsorship of tobacco products

No Indigenous-specific studies were identified regarding elimination of advertising, promotion and sponsorship [18]. Studies were looked for in nine reviews, but none were identified. While there was no Indigenous-specific evidence on which to base recommendations, the importance of eliminating advertising and promotion in ‘denormalising’ smoking was highlighted in the introduction or discussion in several reviews [33, 35, 37]. Ivers [18] reported that the effect of restrictions on tobacco advertising had not been evaluated for Indigenous Australians, but suggested [23] advertising restrictions were likely to have similar impacts as among non-Indigenous people and thus likely to reduce tobacco consumption in Indigenous communities.

### National Aboriginal and Torres Strait Islander Health Plan (NATSIHP) principles and priorities: summary of reported review evidence

The reviews were examined for evidence of the nine principles and priorities (Health Enablers and Whole of Life strategies) of NATSIHP within included interventions. We have not reported information under the NATSIHP Priority of ‘*Health system effectiveness and clinically appropriate care*’ in this overview, as we felt aspects relevant to tobacco control are outlined under NTS Priority 5 (‘*Evidence-based smoking cessation services’*).

Our assessment was dependent on the degree of reporting of these elements by reviewers. The NATSIHP principles and priorities generally relate to ‘how’ interventions are provided and were often discussed in terms of strategies to overcome barriers to NTS interventions for Indigenous Australians, whereas the NTS Priority Areas generally outline ‘what’ interventions are provided. It is likely that in many instances, these more finely nuanced aspects of interventions were not well described, particularly in ‘secondary’ reviews, and it was difficult to determine the degree to which interventions aligned with these principles and priorities. Where described, information relevant to principles and priorities were more commonly identified in the introduction or discussion section of reviews and largely based on the reviewers’ opinions rather than the data presented. Thus, the number of relevant studies was not able to be specified. A summary of findings is displayed in Table 3.

### Principle 1: equality and human rights approach

While not directly a measurement of ‘equality’, one review examined whether there was a different effect of smoking cessation interventions for Indigenous people compared to non-Indigenous people [21]. Other issues related to equality or a human rights approach were mentioned in eight of the 21 included reviews, and no explicit mention could be identified in 12 of the reviews.

Johnston [21] included five studies conducted in New Zealand which reported outcomes among Maori and non-Maori and assessed the degree of cultural adaption in each intervention. They found no significant difference in outcomes by Indigenous status, noting that they were unable to assess if interventions would have been as effective for Maori as non-Maori if there was no cultural adaption. Based on evidence from these studies, they concluded that ‘not all tobacco control interventions can or necessarily need to be culturally adapted for indigenous populations although there are circumstances when this is important.’ [21].

Several reviews highlighted evidence of interventions among non-Indigenous people and discussed the applicability for Indigenous people, considering the type of intervention and whether there was any logical reason why the effect may be different [18–20, 23]. A review of qualitative studies among Indigenous Australian women [25] suggested the differences seen in effectiveness of cessation interventions in pregnancy [26] may be explained by the ‘Extended Parallel Process Model’. This occurs when low levels of self-efficacy are coupled with high levels of fear, leading to responses of denial, avoidance or reactance [43].

DiGiacomo [22] outlined elements of interventions to promote access for Indigenous people, including free or subsidised pharmacotherapy for a portion or the duration of the intervention, cultural tailoring and transport to the intervention site. Several reviews discussed the inequality of the burden of smoking and severe socio-economic deprivation experienced by Indigenous peoples [18, 27, 34] and raised concerns about the impact of price increases [20, 23], the relative paucity of evidence [27] and the importance of addressing the underlying social determinants of health to reduce tobacco use among Indigenous people [19].

### Principle 2: partnership

Reviews by Minichiello [32] and Clifford [31] explicitly explored how tobacco programmes reflected Indigenous self-determination. Partnerships were discussed in nine reviews, and there was no mention of partnerships identified in 10 reviews.

Two reviews examining partnerships within interventions concluded that partnership and enabling Indigenous leadership was crucial to intervention success [31, 32]. Minichiello [32] suggested that this was because interventions will have greater community relevance if programmes are supported and rooted in local community context and highlighted the growing demand from Indigenous communities for control over health services. However, while Minichiello [32] highlighted aspects of partnership in ‘successful’ programmes, including integration of cultural practice; elements of partnerships/community involvement were also identified in programmes which did not demonstrate an effect, and the evidence for an effect of partnerships on intervention success was unclear.

Only one review investigated differential effects of interventions between Indigenous and non-Indigenous people [21]. Based on evidence from included studies, it concluded that while it is ‘preferable’ that interventions were delivered in partnership, there was no evidence that this necessarily impacts on effectiveness [21]. Despite a lack of evidence of effectiveness, numerous reviewers [18–20, 22, 30, 31, 37] consistently emphasised the importance of partnerships with Indigenous communities and organisations. DiGiacomo [22] recommended that partnerships needed to be enabled at all stages of the research process to ensure interventions were culturally safe, with Gould [25] suggesting this could be achieved using community-based participatory research. Ivers [18] highlighted the important role of community health organisations. Furthermore, Ivers [18] reported qualitative research findings that Indigenous Australians preferred tobacco control campaigns that were specifically designed for them and involved Elders and significant community members in their design and delivery. Two reviews provided descriptions of interventions delivered in partnership with communities, involving skills enhancement for school students [27] and a quit support programme provided by a community organisation and mainstream health advocate [29]. Partnerships were suggested by reviewers as particularly critical for developing smoke-free environments [18, 20], reducing duplication of efforts between communities and other agencies [19], increasing the likelihood of dissemination and ensuring that programmes are feasible, acceptable and effective [30].

### Principle 3: engagement

Based on the NATSIHP Principles, ‘engagement’ is similar to ‘partnership’ with a greater emphasis on active involvement of community members, and these aspects were explicitly assessed within studies in two reviews [31, 32]. Engagement was mentioned in nine reviews and not identified in 10 reviews.

Minichiello [32] included qualitative studies which reported Indigenous people’s views on interventions and explored the extent of ‘self-determination’ evident in interventions. Based on findings from the qualitative synthesis, Minichiello [32] suggested there is an increasing ‘community interest’ to prioritise tobacco and a sense of greater self-determination among Indigenous people about developing health interventions. Clifford [31] described the level of Indigenous involvement for each study and found there was involvement in 18 out of 20 studies. Noting that only seven studies reported involving Indigenous people in evaluation, they assert that involvement through all stages of a study is required for ethical practice and this has a number of practical advantages, including achieving change [31].

One review identified engagement as a key element of taking a systems approach to tobacco control [20]. Several reviews highlighted the importance of engagement in improving effectiveness of campaigns [18–20, 26, 34] and planning and implementing interventions [22], particularly smoke-free environments [20]. Johnston [21] described two studies that had engaged Maori staff and communities members in content development and suggested that meaningful engagement and involvement at a formative stage may promote community ownership and acceptability and help to overcome challenges. DiGiacomo [22] suggested that holistic programmes that reflect and respect the values of culture are likely to foster engagement of community members in interventions.

While the concept differs from the intent of engagement in the context of the NATSIHP, a review of qualitative studies of women’s views [37] specifically looked for studies, which measured the level of ‘emotional engagement’ and identification with the messages, which is considered important for addressing nicotine dependence. In this review, Gould [25] found one prevention study using drama in Hawaii, which measured emotional engagement, and reported significant improvements in intention to avoid smoking.

### Principle 4: accountability

There were no explicit assessments of programme accountability identified in any reviews, although we recognise this is hard to define. However, nine reviews discussed the importance of monitoring and evaluation of tobacco interventions.

While few assessments of programme accountability were identified, Minichiello [32] assessed the quality of 85 programme evaluations, of which only 14 were scored ‘strong’, 44 as ‘moderate’ and 27 as ‘weak’. Several reviews in which the inclusion criteria were restricted to randomised controlled trials were only able to identify few eligible studies [21, 34]. Several reviews highlighted the limitations of available evaluations in the literature [23, 31, 33, 36]. A number of reviewers called for greater government accountability in ensuring rigorous implementation [23, 34], analysis of cost-effectiveness and ongoing monitoring [18], and specifically monitoring of compliance with tobacco sales legislation [20].

### Priority/health enabler: social and emotional wellbeing

No reviews were identified that assessed the degree to which interventions addressed ‘social and emotional wellbeing’ of Indigenous people. However, issues related to social and emotional wellbeing, including ‘strengths-based’ and ‘holistic’ approaches, were identified in nine reviews.

Several reviewers highlighted the importance of ‘holistic’ approaches [19, 27], suggesting these approaches are more likely to be acceptable to Indigenous people and promote engagement [22] and be more effective [26, 37]. However, these suggestions were based on expertise and qualitative reports of Indigenous preferences rather than any evidence of an effect on smoking. Ivers [18, 19, 23] emphasised the importance of recognising self-efficacy and strengths of Indigenous Australian people, demonstrated in an Australian study showing that most smokers ‘quit by themselves’. Upton [20] outlined a ‘complex myriad of factors impacting on smoking’, suggesting that socio-ecological models may provide a useful guide for tobacco programmes and that individual-level interventions are likely to be less effective than those which incorporate broader strategies. Gould [37] recommended a community and family approach to support cessation but described qualitative research suggesting family influences could be both a benefit and a hindrance to quitting. Indigenous health worker models of care were identified as models that could support holistic approaches to tobacco control [29].

In contrast, Gould [37] reported findings from a study among Maori people in New Zealand where a mainstream fear-based campaign was more effective than a holistic ‘strength-based’ Maori campaign [37]. However, another review [20] reported market research in Australia suggesting that many smokers may have become desensitised or immune to graphic imagery and shock and that there was growing resentment among some smokers about the use of guilt in tobacco advertising.

### Priority/health enabler: cultural respect

Cultural respect or tailoring was the most commonly reported NATSIHP principle or priority within included reviews. Five reviewers identified aspects of cultural competence or assessed or described tailoring for each study [21, 22, 25, 36, 37], and cultural adaptation of interventions was discussed in 12 reviews.

Johnston [21] examined differential effects in five studies reporting Indigenous and non-Indigenous outcomes and found no significant difference in smoking outcomes between Maori and non-Maori. The reviewers suggest that not all interventions need to be culturally adapted but recognise that they did not assess the effect of interventions without cultural adaptation and that there are circumstances where cultural adaptation is important [21]. Carson [36] also reported no statistically significant difference in pooled effects between culturally tailored and non-tailored cessation strategies. Minichiello [32] suggested cultural tailoring was a ‘critical element of success’, based on synthesised qualitative information, but it was unclear if this was associated with effective interventions.

Gould [37] assessed culturally tailored mass media campaigns and found there was good recall of generic messages but that people preferred culturally targeted messages and these resulted in changes in knowledge and attitudes. A qualitative review of women’s views [25] considered that although it was not possible to assess ‘cultural appropriateness’, studies which underwent formal ethics review were likely to use culturally appropriate methods as this is an essential ethical criterion. DiGiacomo [22] identified cultural tailoring in seven out of nine included studies as an ‘access promoting element’, including the importance of community input and ownership, engagement in planning and implementation, conducting interventions in culturally safe settings and development of culturally tailored resources. There was no evidence identified in a review of the use of traditional Indigenous knowledge for promoting smoking cessation [38]; however, the appendices provided references for ‘culturally adapted programmes’.

Many reviews noted the importance of cultural tailoring interventions where appropriate [20, 22, 23, 26–28, 32, 33, 36], and Ivers reported qualitative evidence that Indigenous people preferred culturally tailored interventions more than interventions that were not culturally tailored [18, 19, 23]. Some reviews suggested involving Elders [18, 19, 33] and Indigenous Health Workers [18, 34] to help develop culturally appropriate non-coercive programmes [18, 19, 23]. The success of one study within an included review attributed success to the creation of a ‘culturally safe space’ [35]. Upton [20] suggested that tobacco messages need to have both personal and cultural relevance to be effective and discusses the importance of using culturally sensitive resources and that interventions should avoid inappropriately ‘telling people how to behave’.

### Priority/health enabler: evidence-based

Only one review [30] appeared to assess if evidence was used to inform the development of included interventions or establish access to data for quality improvement purposes; however, there was some discussion about the use of evidence in 12 reviews.

Clifford [30] reported that ‘encouragingly, all but one included study explicitly reported using evidence-based resources or guidelines, suggesting that Indigenous-specific dissemination studies are primarily implementing best-evidence health interventions.’ The reviewers noted that dissemination strategies used to implement the interventions were generally evidence-based; however, there was a lack of Indigenous-specific evidence [30]. This was reinforced in several reviews noting the importance of using evidence in developing interventions [26, 27, 33, 35] but highlighting the lack of Indigenous-specific available research [24, 28, 34, 37]. Despite this lack of research and the limitations of using research from other populations which may not be generalizable, Ivers [23] suggests that an evidence-based approach ‘may nevertheless ensure a starting point from which Indigenous organisations can make decisions on programme delivery and plan further research and evaluation.’

### Priority/health enabler: human capability

Three reviews appeared to assess the degree of human capability or workforce development within included studies [22, 29, 32], and workforce issues were discussed in 10 reviews.

Minichiello [32] assessed whether interventions included components or focussed on workforce development either directly (e.g. through training) or indirectly (e.g. through involvement in delivery of intervention components) and concluded lack of workforce capacity was a challenge. DiGiacomo [22] described workforce involvement in seven of the nine included interventions and identified it as an important ‘access promoting element’. The high prevalence of smoking among Aboriginal Health Workers was suggested to be a barrier to provision of smoking interventions in several reviews [20, 29]. In a review of the effect of smoking among Aboriginal Health Worker’s on the provision of tobacco control interventions [29], eight studies highlighted a need for workforce development, including training for health workers to deliver interventions, and support to quit smoking for health workers who smoked themselves.

The importance of building Indigenous workforce capability was highlighted in many reviews [20, 21, 26, 31–33], and the ‘challenges of the Aboriginal Health Worker role’ was identified as a key construct in a qualitative review [25]. Passey [26] highlighted a lack of a protocol and a lack of smoking cessation support skills as barrier’s to supporting pregnant Indigenous smokers to quit, and Johnston [21] suggested workforce development could help to address implementation challenges for smoking cessation. Several reviews reported the effects of studies to improve health professional delivery of brief interventions, which found improvements in skills and confidence after the interventions compared to before the interventions [19, 23, 35].

### Priority: whole of life approaches

No reviews that explicitly reported ‘whole of life’ approaches to tobacco control. However, specific ‘life stages’ (e.g. adolescence, pregnancy) were discussed in seven reviews.

Several reviews discuss interventions that are provided for Indigenous people at specific life stages, such as adolescence and pregnancy [18, 20, 32–34, 37]. There were also targeted reviews evaluating interventions for adolescents [27, 28] and pregnant women [26], which highlighted a lack of evidence of effective interventions during these two important life stages in relation to smoking initiation and impact on infant health.

## Discussion

In this overview of reviews, numerous reviewers concluded that multifaceted interventions which incorporate Indigenous leadership, partnership and engagement [18–20, 22, 30, 32, 36] and cultural tailoring [25, 29, 32, 33, 36, 37] where appropriate [21] are necessary to reduce the burden of tobacco-related disease among Indigenous peoples [18–20, 25, 32, 33, 36]. Evidence synthesised under the NTS priorities related to specific intervention strategies and was generally well described (i.e. ‘the what’), while evidence under the NATSIHP principles and priorities tended to be more related to process or implementation aspects of interventions and were less well described (i.e. ‘the how’). Despite some consensus among reviewer conclusions as summarised above, we found there was generally limited evidence regarding effectiveness of strategies among Indigenous populations, for each of the NTS and NATSIHP principles and priorities, on which to base these assertions [23, 30, 31]. The review quality was variable and there was varying assessment of risk of bias of included studies, with no GRADE assessments conducted within the included reviews, so the basis of confidence in these reviewer conclusions was unclear.

Our findings are consistent with those reported by other authors demonstrating a lack of tobacco research among ‘special’ [44], minority [45, 46], and other populations [47], including Indigenous pregnant women [48] and adolescents [49]. A review of tobacco research outputs among Indigenous people was consistent with our findings that the majority of peer-reviewed publications focussed on cessation [50]. The conclusions of the majority of reviewers in our overview are also consistent with those reported in reviews in the general population [51–54].

### Strengths and limitations

This overview has a number of strengths, including a systematic approach to searching, extracting and appraising the literature in relation to the NTS and NATSIHP principles and priorities. This study also includes Indigenous governance and leadership and thus provides a novel insight into Indigenous tobacco control. While an overview approach is appropriate to address the aims of this project, there are limitations inherent in an overview approach. First, the findings rely largely on secondary interpretations of primary studies reported in reviews, and some aspects looked for in this overview may not have been considered in included reviews or there was inconsistent reporting of study characteristics across reviews, which limits information available to interpret the evidence. To deal with this, we have assessed where domains appeared to be looked for and have specified where reviewer suggestions were based on reported evidence or if it was unclear. A second limitation is that studies that are more recent may not yet be included in reviews, such as the ‘Talking about the Smokes’ project in Australia [55]. Third, there was some overlap of studies within reviews, and there may have been an emphasis placed on the same findings reported between reviews. However, the reviews incorporated in this overview included different subsets of studies and referenced different sources for the same studies, and the limited overlap may account for differences in some findings. These differences in coverage of the evidence on tobacco control strategies for Indigenous peoples are not fully explained by difference in the scope or inclusion criteria of reviews. As such, no single review provides complete coverage of all available evidence, supporting the overview approach. There were also different synthesis methods used, including vote counting and summing ‘statistically significant results’, with few reviews weighting and pooling results. Fourth, we may have missed general reviews that include studies with Indigenous people, and these may not have been identified and included unless Indigenous people were explicitly mentioned in the abstract. Finally, the majority of ‘grey literature’ is from Australia and was only accessible by Australian authors, collected through word-of-mouth rather than extensive google and website searching. It is possible that there is grey literature from other countries that has not been included in this overview, although one included review [32] had a significantly larger proportion ‘grey literature’ from Canada. However, as this overview aims to synthesis review evidence for tobacco control for Indigenous Australians, and therefore, the benefits of including this grey literature outweigh any limitations.

### Implications of overview findings

There is limited Indigenous-specific evidence to support many of the NTS and NATSIHP principles and priorities. However, the continuation, implementation and evaluation of effective interventions are urgently needed to continue to reduce tobacco use among Indigenous Australians. It is highly likely that evidence from other populations, both what has and has not been effective, may be applicable for Indigenous populations. For example, evidence within this overview suggests smoking cessation interventions can be effective for Indigenous people. A study of attitudes to smoking in Australia [56] showed a similar proportion of Indigenous people compared to non-Indigenous people want to quit (70%), have attempted to quit (69%) and live in smoke-free homes (53%) and workplaces (88%). Fewer Indigenous Australian people sustain quitting or ‘agree’ with social norms that disapprove of smoking [56]. However, these findings were similar to a review of smoking interventions among ethnic minority populations in the USA reporting that although people in minority populations were more likely to smoke and wanted to stop smoking, they were less likely to receive quitting advice and less likely to be able to quit [57]. The lower quit rates are thought to reflect social difficulties which provide less support for quit maintenance [20].

However, evidence from other populations may not always be applicable for Indigenous peoples [58]. For example, while reducing the affordability of tobacco products and regulation are important tobacco control strategies encourage the de-normalisation of smoking, there is limited Indigenous-specific evidence regarding whether increasing tax on tobacco products are seen as acceptable [59] and there are concerns about the potential financial effects on Indigenous people [56]. A review of high-level policies (tax and price, smoking location restrictions and sales restrictions) found that few studies assessed the financial impact [57] and recommended research was needed to evaluate the unintended impact of these interventions on vulnerable subpopulations [60]. Another example is the evidence suggesting that mass media campaigns are effective in changing knowledge and attitudes among Indigenous people in this overview. There is debate about positive ‘strengths-based’ versus negative narrative messages, and how these are processed [61]. Personalized negative messages, which arouse strong emotions, are likely to be more effective and have been very effective at arousing strong emotional responses [61, 62]. However, an included review in Australia reporting market research suggested ‘many smokers have become immune to shock’ and that there was growing resentment about the use of guilt in tobacco advertising [20] and experts query whether messages that elicit high levels of positive emotion might be equally effective [61]. Concerns about negative messages were raised in consultation for the NATSIHP, as theoretical evidence suggests deficit discourse can potentially contribute to ‘internalised racism’ where Indigenous people themselves start to believe negative stereotypes and messages about their own abilities and intrinsic worth [63]. This may be particularly relevant for tobacco control as it may contribute to low levels of self-efficacy. The Extended Parallel Process Model proposes a mechanism where high ‘perceptions of risk’ (elicited by strong negative messages) coupled with low levels of ‘self-efficacy’ may be associated with avoidance behaviour [43, 64]. One reviewer in this overview suggests this could be a possible factor affecting low rates of smoking cessation among Indigenous women [25], highlighting the importance of understanding message processing. Further examples where evidence from other populations may not be applicable include tailoring to specific developmental stages and cultural values, which may be important for addressing tobacco use among adolescents [65]. It is also likely that tobacco control strategies will be cost-effective and have a significant impact on improving health equality for Indigenous peoples [66]. However, the parameters to assess the cost-effectiveness of tobacco control interventions are significantly different for Indigenous people, due to differences in disease burden, cost of interventions and lack of certainty regarding effectiveness [66].

The lack of Indigenous-specific evidence raises questions about how to develop and design interventions when action is urgently needed and the degree of generalisability of evidence from other populations is not clear [67]. Evidence-based public health decisions are made on the best available evidence, but also involve using data and information systems systematically, applying programme-planning frameworks, engaging the community in decision-making, and conducting sound evaluation to share learning [68]. Where there is limited Indigenous-specific evidence, these additional components of public health decision-making are particularly critical to improve the acceptability and effectiveness of interventions and mitigate risks. This includes careful planning which considers the context, rationale and ‘logic’ of interventions [69], and Indigenous leadership, partnership and engagement as concluded by many reviewers in this overview [18–20, 22, 30, 32, 36]. Incorporating flexible evaluation plans with short-term reflective cycles, such as action research, can also help to mitigate risks of uncertainty in the evidence by enabling early detection and response to unforeseen consequences. There have also been calls for greater Indigenous involvement in evaluation and research [31, 70], with facilitators including relationship and partnership building, employing Indigenous staff, drawing on Indigenous knowledge models, targeted recruitment techniques and adapting study material. Greater uniformity of outcome measures in evaluations has also been suggested [71], particularly in Indigenous settings, to facilitate evidence synthesis and redress limitations of studies with low power due to small sample sizes.

## Conclusions

Reducing tobacco-related inequity requires a comprehensive approach. This overview outlines existing review evidence and its alignment with a ‘comprehensive framework for Aboriginal and Torres Strait Islander Tobacco control’ (CATs), combining the NTS and the NATSIHP principles and priorities. While review quality is variable, there is generally limited Indigenous-specific evidence of impacts on smoking rates; however, most reviewers recommended multifaceted interventions incorporating all NTS priorities, including pricing and regulation, mass media campaigns, smoke-free spaces and policies and smoking cessation (‘the what’). Reviewers also described rationale for how elements of the NATSHIP principles and priorities (‘the how’) may improve acceptability, effectiveness, and implementation of the NTS priorities, including partnerships and engagement, cultural tailoring, and a focus on social determinants and social and emotional wellbeing, and workforce development, using evidence and increasing accountability. The risks of adapting evidence from other settings are likely to be mitigated by incorporating all components of evidence-based public health decision-making, including programme planning and logic, active community involvement and flexible responsive evaluation plans. There is a need for Indigenous-specific research regarding the impact of pricing measures; interventions to reduce tobacco use among adolescents, pregnant women, adolescents and adults experiencing mental illness or imprisonment; and linguistically diverse Indigenous people. There is also a need for Indigenous-specific evidence regarding interventions using social media and mobile applications, electronic cigarettes, ‘strengths-based’ holistic approaches and how to culturally tailor interventions, protecting communities from industry interference, and interventions in ‘pubs, clubs and restaurants’ in Indigenous communities.

## Additional files

Additional file 1: PRISMA checklist.

Additional file 2: Data extraction guidance.

Additional file 3: Databases searched.

Additional file 4: Detailed list of search terms.

Additional file 5: Table of reviews excluded after assessment for eligibility in full text review and reasons for exclusion.

Additional file 6: Detailed AMSTAR rating for each included review.

Additional file 7: Matrix of references to studies included in reviews included in the Overview.

## Abbreviations

AMSTAR: Assessing the Methodological Quality of Systematic Reviews
CATs: A comprehensive approach to Aboriginal and Torres Strait Islander tobacco control
GRADE: Grading of Recommendations Assessment Development and Evaluation
NATSIHP: National Aboriginal and Torres Strait Islander Health Plan
NTS: National Tobacco Strategy
WHO: World Health Organization

## Footnotes

1 In this overview, domains related to NTS key priorities of *6.4 Bolster and build on existing programs and partnerships to reduce smoking rates among Aboriginal and Torres Strait Islander people* and *6.5 strengthen efforts to reduce smoking among populations with high smoking prevalence* are not included as relevant information is synthesized under NATSIHP principles.

2 The NATSIHP domain for the health enabler of *Health system effectiveness and clinically appropriate care* was not included in this overview as information related to tobacco control interventions was synthesized under NTS priorities.
