# Supplementary material for: Evidence for a comprehensive approach to Aboriginal tobacco control to maintain the decline in smoking: an overview of reviews among Indigenous peoples
Source: Syst Rev. 2017 Jul 10;6:135. doi: 10.1186/s13643-017-0520-9 (PMC5504765; doi:10.1186/s13643-017-0520-9)
Supplement: Supplementary file 2 — Data extraction guidance. [file 13643_2017_520_MOESM2_ESM.pdf]

Section no.	Section name	Field header (including instructions) - copied from DE form	Codes/additional guidance
1.0a	General information	1.0a General information	
1.0a	General information	1.1 Review ID<br>[first author family name and year]	
1.0a	General information	1.2 Reference	
1.0a	General information	1.3 Date form completed (dd/mm/yyyy)	
1.0a	General information	1.4 Name/ID of person extracting data	List SP, SB, CC
1.0a	General information	1.5 Associated references<br><br>[Insert: Review ID if included OR ref if other]	
1.0a	General information	1.6 Author contact details [email]	
1.0a	General information	1.7 Publication type [select]	Systematic review<br>Literature review (no evidence of any methods or search strategy)<br>Meta-analysis<br>Other [specify]
1.0a	General information	1.8 Start search and End date	
1.0a	General information	1.9 Inclusion decision	Included<br>Excluded<br>Unclear<br>Other
1.0b	Scope and aims	1.0b Scope and aims	
1.0b	Scope and aims	1.9a Aims of the review - verbatim extract	
1.0b	Scope and aims	1.9b Briefly summarise the aims of the review	
1.0b	Scope and aims	1.10 Scope: Did the review focus on studies involving Indigenous populations exclusively or were studies in other populations eligible?<br><br>[Select: Indigenous studies only or broader?]	Studies involving Indigenous populations (studies involving other populations were excluded)<br>Studies involving Indigenous populations and other populations<br>Other [describe]
1.0b	Scope and aims	1.11 Scope: Did the review focus on tobacco programs only?<br><br>[Select: Tobacco or broader]	Specific targeted tobacco programs/interventions<br>Broader review which includes a tobacco program component<br>Other [describe]
1.0b	Scope and aims	1.12 Were there any potential conflicts of interest?<br><br>[YES if Tobacco industry funding or declared COIs; UNCLEAR if funding not specified]	Yes - Authors received tobacco industry funding or had other potential conflicts<br>No - Authors did not receive tobacco industry funding or have other potential conflicts<br>Unclear - Funding sources and interests not reported<br>Other [specify]
1.0b	Scope and aims	1.13 Study funding source - describe<br><br>[If Tobacco industry, extract name and role of funders.]	
1.0b	Scope and aims	1.14 Notes	
2.00	Included studies	2.0 Included studies<br>[One author to COMPLETE MATRIX IN<br>SEP WORKSHEET]	Also separate worksheet with matrix of studies by review

Section no.	Section name	Field header (including instructions) - copied from DE form	Codes/additional guidance
2.00	Included studies	2.1 Type of included studies<br><br>[Delete designs that were not eligible for inclusion; note number found of each type]	Randomised Controlled Trials and Cluster RCT's [number]<br>Non-randomised [number]<br>Qualitative [number]
2.00	Included studies	2.2 Total number of included studies	
2.00	Included studies	2.3 Type of included studies<br><br>[extract or notes if needed]<br><br>[Should we include info about study quality in this sxn or somewhere??]	
3.0a	Population & setting - DESCRIPTION	3.0a Population & setting - DESCRIPTION	
3.0a	Population & setting - DESCRIPTION	3.1 Population - Indigenous/ethnicity - what the reviewers looked for<br><br>[select, based on PICO]	All Aboriginal<br>All Torres Strait Islander<br>All Aboriginal and Torres Strait Islander<br>Indigenous, including Aboriginal and Torres Strait Islander<br>Other Indigenous: (specify)<br>Indigenous and non-indigenous<br>Specific ethnic group: (specify)
3.0a	Population & setting - DESCRIPTION	3.2 Population & setting - brief description of what the reviewers looked for as stated in report<br><br>[Capture any sub-groups or particular settings, and definitions of these, as specified in PICO; or 'no restrictions' if nothing specified beyond 3.1. Note page no. or location in report]	
3.0a	Population & setting - DESCRIPTION	3.3 Population & setting - brief description of what the reviewers found as stated in report<br><br>[Any description that helps characterise and quantify the included studies, not picked up in the coding. Note page no. or location in report]	
3.0a	Population & setting - DESCRIPTION	3.4 Population - number of studies involving Aboriginal or Torres Strait Islander populations	
3.0a	Population & setting - DESCRIPTION	3.5 Type of smoking characteristics<br><br>[extract; Note page no. or location in report]	
3.0a	Population & setting - DESCRIPTION	3.6 Population & setting - notes or additional extracts	
3.0b	Population & setting - CODING	3.0b Population & setting - CODING	

Section no.	Section name	Field header (including instructions) - copied from DE form	Codes/additional guidance
3.0b	Population & setting - CODING	3.6a Pop - Men - PICO	1=Focus of the review PICO/question<br>2=Not mentioned in the PICO/ review question but eligible<br>3=Not looked for (ineligible)<br>4=Unclear/other (describe)
3.0b	Population & setting - CODING	3.6b Pop - Men - Were studies found?	1=Found and included studies involving this population (? No of studies ?)<br>2=Did not find eligible studies involving this population<br>3=Found but excluded studies involving this population<br>4=Not reported<br>5=Unclear/other (describe)<br>6=N/A (if pop ineligible)
3.0b	Population & setting - CODING	3.6c Pop - Women - PICO	as per 3.6a
3.0b	Population & setting - CODING	3.6d Pop - Women - Were studies found?	as per 3.6b
3.0b	Population & setting - CODING	3.6e Pop - Pregnant women - PICO	as per 3.6a
3.0b	Population & setting - CODING	3.6f Pop - Pregnant women - Were studies found?	as per 3.6b
3.0b	Population & setting - CODING	3.6g Pop - Families of smokers - PICO	as per 3.6a
3.0b	Population & setting - CODING	3.6h Pop - Families of smokers - Were studies found?	as per 3.6b
3.0b	Population & setting - CODING	3.6i Pop - Adolescents - PICO	as per 3.6a
3.0b	Population & setting - CODING	3.6j Pop - Adolescents -Were studies found?	as per 3.6a
3.0b	Population & setting - CODING	3.6k Pop - School students - PICO	as per 3.6b
3.0b	Population & setting - CODING	3.6l Pop - School students - Were studies found?	as per 3.6a
3.0b	Population & setting - CODING	3.6m Pop - Prisoners - PICO	as per 3.6b
3.0b	Population & setting - CODING	3.6n Pop - Prisoners - Were studies found?	as per 3.6a
3.0b	Population & setting - CODING	3.6o Pop - Juvenile justice - PICO	as per 3.6b
3.0b	Population & setting - CODING	3.6p Pop - Juvenile justice - Were studies found?	as per 3.6a
3.0b	Population & setting - CODING	3.6q Pop - Low SES - PICO	as per 3.6a
3.0b	Population & setting - CODING	3.6rPop - Low SES - Were studies found?	as per 3.6b
3.0b	Population & setting - CODING	3.6sPop - CALD (culturally and linguisticaly diverse) - PICO<br><br>[select and indicate which communities]	as per 3.6a

Section no.	Section name	Field header (including instructions) - copied from DE form	Codes/additional guidance
3.0b	Population & setting - CODING	3.6t Pop - CALD (indicate which) - Were studies found?	as per 3.6b
3.0b	Population & setting - CODING	3.6u Pop - Rural and remote - PICO	as per 3.6a
3.0b	Population & setting - CODING	3.6v Pop - Rural and remote - Were studies found?	as per 3.6b
3.0b	Population & setting - CODING	3.6w Pop - Health service clients - PICO	as per 3.6a
3.0b	Population & setting - CODING	3.6xPop - Health service clients - Were studies found?	as per 3.6a
3.0b	Population & setting - CODING	3.6y Pop - Health service clients - PICO	as per 3.6b
3.0b	Population & setting - CODING	3.6z Pop - Health service clients - Were studies found?	as per 3.6a
3.0b	Population & setting - CODING	3.6aa Pop - Mental health clients - PICO	as per 3.6b
3.0b	Population & setting - CODING	3.6ab Pop - Mental health clients - Were studies found?	as per 3.6a
3.0b	Population & setting - CODING	3.6ac Pop - Aboriginal health workers - PICO	as per 3.6b
3.0b	Population & setting - CODING	3.6ad Pop - Aboriginal health workers - Were studies found?	as per 3.6a
3.0b	Population & setting - CODING	3.6ae Pop - GPs - PICO	as per 3.6a
3.0b	Population & setting - CODING	3.6af Pop - GPs - Were studies found?	as per 3.6b
3.0b	Population & setting - CODING	3.6ag Pop - Health professionals - PICO	as per 3.6a
3.0b	Population & setting - CODING	3.6ah Pop - Health professionals - Were studies found?	as per 3.6b
3.0b	Population & setting - CODING	3.6ai Pop - Community organisations - PICO	as per 3.6a
3.0b	Population & setting - CODING	3.6aj Pop - Community organisations - Were studies found?	as per 3.6b
3.0b	Population & setting - CODING	3.6ak Pop - Local government - PICO	as per 3.6a
3.0b	Population & setting - CODING	3.6al Pop - Local government -Were studies found?	as per 3.6a
3.0b	Population & setting - CODING	3.6am Pop - Pubs, clubs, restaurants - PICO	as per 3.6b
3.0b	Population & setting - CODING	3.6an Pop - Pubs, clubs, restaurants - Were studies found?	as per 3.6a
3.0b	Population & setting - CODING	3.6ao Pop - Tobacco retailers - PICO	as per 3.6b
3.0b	Population & setting - CODING	3.6ao Pop - Tobacco retailers - Were studies found?	as per 3.6a

Section no.	Section name	Field header (including instructions) - copied from DE form	Codes/additional guidance
3.0b	Population & setting - CODING	3.6a Pop - Other (specify)	as per 3.6b
3.0b	Population & setting - CODING	3.3 Other relevant socio-demographics not covered in coding [extract]	
4.00	Intervention & comparisons - DESCRIPTION	4.0 Intervention & comparisons - DESCRIPTION	
4.00	Intervention & comparisons - DESCRIPTION	4.0a Intervention(s) - brief description of what the reviewers looked for as stated in report<br><br>[Notes any categorisation of types strategies, intervention elements or content as specified in PICO; Note page no. or location in report]	
4.00	Intervention & comparisons - DESCRIPTION	4.0b Intervention(s) - brief description of what the reviewers found as stated in report	
4.00	Intervention & comparisons - DESCRIPTION	4.0c Comparisons - brief description of what the reviewers looked for as stated in report	
4.00	Intervention & comparisons - DESCRIPTION	4.0d Comparison - brief description of what the reviewers found as stated in report	
4.00	Intervention & comparisons - DESCRIPTION	4.0e Intervention & comparison - notes or additional extracts	
4.10	NTS priorities: REDUCE AFFORDABILITY	4.1 Intervention - NTS priorities:<br>REDUCE AFFORDABILITY	
4.10	NTS priorities: REDUCE AFFORDABILITY	4.1a Did the reviewers consider strategies to 'Reduce affordability' ?<br><br>[select from list]	Yes<br>No<br>Not reported/insufficient detail to tell<br>Other/unclear [specify]
4.10	NTS priorities: REDUCE AFFORDABILITY	4.1b No. studies evaluating strategies to 'Reduce affordability'.<br><br>[include page number and location of information in report]	
4.10	NTS priorities: REDUCE AFFORDABILITY	4.1c Description of strategies to 'Reduce affordability'<br><br>[extract or precis, include page number and location of information in report]	
4.10	NTS priorities: REDUCE AFFORDABILITY	4.1d Level of strategies to 'Reduce affordability'<br><br>[select from list]	Population/legislation level<br>Community level<br>Individual level<br>Multi-level: (specify)<br>Varied across studies: (specify)<br>Not reported/insufficient detail to tell<br>Unsure - discussion required

Section no.	Section name	Field header (including instructions) - copied from DE form	Codes/additional guidance
4.10	NTS priorities: REDUCE AFFORDABILITY	4.1e Program aims for strategies to 'Reduce affordability'<br><br>[Select list; delete those that DON'T apply. This info isn't critical, so give an indication only OR note if can't tell.]	• To prevent smoking uptake (N=?)<br>• To reduce exposure to second-hand smoke (N=?)<br>• To increase the capacity of health/community organisations in tobacco control (N=?)<br>• To promote smoke free health services (N=?)<br>• To reduce smoking in the workplace (N=?)<br>• To reduce tobacco sales and availability (N=?)<br>• Other (please specify) (N=?)<br>• Not reported/insuffient detail to tell<br>• Unsure - discussion required
4.10	NTS priorities: REDUCE AFFORDABILITY	4.1f What did the initiatives to 'Reduce affordability' involve?<br><br>[Select list; delete those that DON'T apply. This info isn't critical, so give an indication only OR note if can't tell.]	Social marketing (television)<br>Social marketing (radio)<br>Social marketing (print media)<br>Social marketing (online)<br>Social marketing (social media)<br>Signage / billboards<br>Printed resources<br>Media promotion / PR<br>Smoking cessation brief interventions<br>Free or subsidised NRT (nicotine replacement therapy)<br>Counselling and support (face-to-face)<br>Counselling and support (online)<br>Counselling and support (telephone)<br>Referral to quit smoking services<br>Policy development<br>Training/support of policy or projects within community organisations<br>Training/support for local government<br>Training/support for tobacco control volunteers<br>Community or NGO grant schemes<br>Tobacco control advocacy<br>Tobacco research and evaluation<br>Support for the implementation of tobacco control policies<br>Monitoring and/or enforcement of compliance with regulations<br>Other (please specify)
4.20	NTS priorities: TOBACCO CONTROL POLICIES	4.2 Intervention - NTS priorities:<br>TOBACCO CONTROL POLICIES	
4.20	NTS priorities: TOBACCO CONTROL POLICIES	4.2a Did the reviewers consider 'tobacco control policies'?<br><br>[select from list]	As per 4.1a-f
4.20	NTS priorities: TOBACCO CONTROL POLICIES	4.2b No. studies evaluating 'tobacco control policies'.	As per 4.1a-f
4.20	NTS priorities: TOBACCO CONTROL POLICIES	4.2c Description of the 'tobacco control policies' evaluated<br><br>[extract or precis, include page number and location of information in report]	As per 4.1a-f

Section no.	Section name	Field header (including instructions) - copied from DE form	Codes/additional guidance
4.20	NTS priorities: TOBACCO CONTROL POLICIES	4.2d Level of 'tobacco control policies'<br><br>[select from list]	As per 4.1a-f
4.20	NTS priorities: TOBACCO CONTROL POLICIES	4.2e Program aims for 'tobacco control policies'<br><br>[Select list; delete those that DON'T apply. This info isn't critical, so give an indication only OR note if can't tell.]	As per 4.1a-f
4.20	NTS priorities: TOBACCO CONTROL POLICIES	4.2f What did the 'tobacco control policies' involve?<br><br>[Select list; delete those that DON'T apply. This info isn't critical, so give an indication only OR note if can't tell.]	As per 4.1a-f
4.30	NTS priorities: REGULATION OF CONTENTS, PRODUCT DISCLOSURE, SUPPLY	4.3 Intervention - NTS priorities:<br>REGULATION OF CONTENTS, PRODUCT DISCLOSURE, SUPPLY	
4.30	NTS priorities: REGULATION OF CONTENTS, PRODUCT DISCLOSURE, SUPPLY	4.3a Did the reviewers consider 'regulation of contents, product disclosure and supply' ?<br><br>[select from list]	As per 4.1a-f
4.30	NTS priorities: REGULATION OF CONTENTS, PRODUCT DISCLOSURE, SUPPLY	4.3b No. studies evaluating strategies to 'regulate contents, product disclosure and supply'.	As per 4.1a-f
4.30	NTS priorities: REGULATION OF CONTENTS, PRODUCT DISCLOSURE, SUPPLY	4.3c Description of the strategies involving 'regulation of contents, product disclosure and supply' evaluated<br><br>[extract or precis, include page number and location of information in report]	As per 4.1a-f
4.30	NTS priorities: REGULATION OF CONTENTS, PRODUCT DISCLOSURE, SUPPLY	4.3d Level of 'regulation of contents, product disclosure and supply'<br><br>[select from list]	As per 4.1a-f
4.30	NTS priorities: REGULATION OF CONTENTS, PRODUCT DISCLOSURE, SUPPLY	4.3e Program aims for 'regulation of contents, product disclosure and supply'<br><br>[Select list; delete those that DON'T apply. This info isn't critical, so give an indication only OR note if can't tell.]	As per 4.1a-f
4.30	NTS priorities: REGULATION OF CONTENTS, PRODUCT DISCLOSURE, SUPPLY	4.3f What did the 'regulation of contents, product disclosure and supply' involve?<br><br>[Select list; delete those that DON'T apply. This info isn't critical, so give an indication only OR note if can't tell.]	As per 4.1a-f
4.40	NTS priorities: MASS MEDIA	4.4 Intervention - NTS priorities: MASS MEDIA	

Section no.	Section name	Field header (including instructions) - copied from DE form	Codes/additional guidance
4.40	NTS priorities: MASS MEDIA	4.4a Did the reviewers consider 'mass media' strategies?<br><br>[select from list]	As per 4.1a-f
4.40	NTS priorities: MASS MEDIA	4.4b No. studies evaluating 'mass media' strategies.	As per 4.1a-f
4.40	NTS priorities: MASS MEDIA	4.4c Description of 'mass media' strategies evaluated<br><br>[extract or precis, include page number and location of information in report]	As per 4.1a-f
4.40	NTS priorities: MASS MEDIA	4.4d Level of 'mass media' strategies<br><br>[select from list]	As per 4.1a-f<br><br>• To prevent smoking uptake (N=?)<br>• To reduce exposure to second-hand smoke (N=?)<br>• To increase the capacity of health/community organisations in tobacco control (N=?)<br>• To promote smoke free health services (N=?)<br>• To reduce smoking in the workplace (N=?)<br>• To reduce tobacco sales and availability (N=?)<br>• Other (please specify) (N=?)<br>• Not reported/insuffient detail to tell<br>• Unsure - discussion required
4.40	NTS priorities: MASS MEDIA	4.4e Program aims for 'mass media' strategies<br><br>[Select list; delete those that DON'T apply. This info isn't critical, so give an indication only OR note if can't tell.]	As per 4.1a-f
4.40	NTS priorities: MASS MEDIA	4.4f What did the 'mass media' strategies involve?<br><br>[Select list; delete those that DON'T apply. This info isn't critical, so give an indication only OR note if can't tell.]	As per 4.1a-f
4.50	NTS priorities: CESSATION SERVICES	4.5 Intervention - NTS priorities:<br>CESSATION SERVICES	
4.50	NTS priorities: CESSATION SERVICES	4.5a Did the reviewers consider 'cessation services' ?<br><br>[select from list]	As per 4.1a-f
4.50	NTS priorities: CESSATION SERVICES	4.5b No. studies evaluating 'cessation services'.	As per 4.1a-f
4.50	NTS priorities: CESSATION SERVICES	4.5c Description of 'cessation services' evaluated<br><br>[extract or precis, include page number and location of information in report]	As per 4.1a-f
4.50	NTS priorities: CESSATION SERVICES	4.5d Level of intervention for 'cessation services'<br><br>[select from list]	As per 4.1a-f
4.50	NTS priorities: CESSATION SERVICES	4.5e Program aims for 'cessation services'<br><br>[Select list; delete those that DON'T apply. This info isn't critical, so give an indication only OR note if can't tell.]	As per 4.1a-f

Section no.	Section name	Field header (including instructions) - copied from DE form	Codes/additional guidance
4.50	NTS priorities: CESSATION SERVICES	4.5f What did the 'cessation services' involve?<br><br>[Select list; delete those that DON'T apply. This info isn't critical, so give an indication only OR note if can't tell.]	As per 4.1a-f
4.60	NTS priorities: SMOKE FREE WORKPLACES ETC	4.6 Intervention - NTS priorities:<br>SMOKE FREE WORKPLACES ETC	
4.60	NTS priorities: SMOKE FREE WORKPLACES ETC	4.6a Did the reviewers consider strategies involving 'smoke-free workplaces, public places etc' ?<br><br>[select from list]	As per 4.1a-f
4.60	NTS priorities: SMOKE FREE WORKPLACES ETC	4.6b No. studies evaluating strategies involving 'smoke-free workplaces, public places etc' .	As per 4.1a-f
4.60	NTS priorities: SMOKE FREE WORKPLACES ETC	4.6c Description of the strategies involving 'smoke-free workplaces, public places etc' evaluated<br><br>[extract or precis, include page number and location of information in report]	As per 4.1a-f
4.60	NTS priorities: SMOKE FREE WORKPLACES ETC	4.6d Level of strategies involving 'smoke-free workplaces, public places etc'<br><br>[select from list]	As per 4.1a-f
4.60	NTS priorities: SMOKE FREE WORKPLACES ETC	4.6e Program aims for strategies involving 'smoke-free workplaces, public places etc'<br><br>[Select list; delete those that DON'T apply. This info isn't critical, so give an indication only OR note if can't tell.]	As per 4.1a-f
4.60	NTS priorities: SMOKE FREE WORKPLACES ETC	4.6f What did the 'smoke-free workplaces, public places etc' strategies involve?<br><br>[Select list; delete those that DON'T apply. This info isn't critical, so give an indication only OR note if can't tell.]	As per 4.1a-f
4.7-4.15		4.7-4.15 Intervention - 'the how'<br>SEE GUIDANCE WORKSHEET FOR CRITERIA	
4.70	Equity	4.7 EQUITY	
4.70	Equity	4.7a Did the reviewers look for evidence of a human rights or equity focussed approach in the design/implementation/evaluation of strategies?<br><br>[Select from list. 'Yes' if reviewers explicitly looked for or mentioned whether studies (1) considered differential effects in any sub pops (e.g. through use of a logic model), (2) specifically addressed equity questions in their research design (e.g. process evaluation to address equity considerations or access), (3) examined applicability - generalisation to different sub-populations.]	Y - tool/criteria used to assess each study<br>Y - described for each study<br>Y - overall summary, but not study-by-study<br>Y - mentioned but no detail<br>N - not mentioned<br>other/unclear: (specify)

Section no.	Section name	Field header (including instructions) - copied from DE form	Codes/additional guidance
4.70	Equity	4.7b If 'yes' to previous, what did the reviewers look for?<br><br>[Note any explicit mention of a human rights or equity focussed approach such as examining (1) differential effects in any sub pops (e.g. through use of a logic model), (2) any aspects of research design specifically addressing equity questions (e.g. process evaluation to address equity considerations or access), (3) examination of applicability - generalisation to different sub-populations.]	
4.70	Equity	4.7c Brief description of findings, as stated in review.<br><br>[extract or notes; page no / location in report]	
4.80	Partnership	4.8 PARTNERSHIP	
4.80	Partnership	4.8a Did the reviewers look for evidence of formal partnership and shared ownership in the design and implementation of strategies?<br><br>[Select from list. 'Yes' if the reviewers looked for evidence of working in formal partnership (Meteor indicator), such as working with Indigenous community organisations to provide a mechanism for engagement (national health data dictionary, NATSHP)]	as per 4.7
4.80	Partnership	4.8b If 'yes' to previous, what did the reviewers look for?<br><br>[Note any explicit mention of formal partnerships such as working with Indigenous community organisations to design and implement strategies (national health data dictionary) as a mechanism for engagement (NATSHP)]	as per 4.7
4.80	Partnership	4.8c Brief description of findings, as stated in review.<br><br>[extract or notes; page no / location in report]	
4.90	Engagement	4.9 ENGAGEMENT	

Section no.	Section name	Field header (including instructions) - copied from DE form	Codes/additional guidance
4.90	Engagement	4.9a Did the reviewers look for evidence of engagement (informal partnership and participation) with Indigenous people and organisations?<br><br>[Select from list. 'Yes' if the reviewers looked for evidence of whether: (1) studies reported on people's views on content, context or delivery of the intervention, (2) took account of people's views in the design of the intervention, (3) the interventions were designed in consultation or collaboration with communities, (4) there an explicit plan for collective action [adapted from Oliver].<br><br>Definition from NATSIHP "There is a full and ongoing participation by Aboriginal and Torres Strait Islander people and organisations in all levels of decision-making affecting their health needs"	as per 4.7
4.90	Engagement	4.9b If 'yes' to previous, what did the reviewers look for?<br><br>[Note any explicit mention of looking for evidence of whether: (1) studies reported on people's views on content, context or delivery of the intervention, (2) took account of people's views in the design of the intervention, (3) the interventions were designed in consultation or collaboration with communities, (4) there an explicit plan for collective action [adapted from Oliver].	as per 4.7
4.90	Engagement	4.9c Brief description of findings, as stated in review.<br><br>[extract or notes; page no / location in report]	
4.10	Accountability/monitoring	4.10 ACCOUNTABILITY/MONITORING	
4.10	Accountability/monitoring	4.10a Did the reviewers examine whether structures were in place for regular monitoring and review of implementation of the intervention?<br><br>[Select from list. 'Yes' if mentioned looking for evidence of structures for regular monitoring/review of strategies as measured by indicators of success (NATSIHP) def)]	as per 4.7
4.10	Accountability/monitoring	4.10b If 'yes' to previous, what did the reviewers look for?<br><br>[Note any explicit mention of looking for evidence of structures or plans for regular monitoring/review of strategies, such as measured by indicators of success. (NATSIHP) def)]	as per 4.7
4.10	Accountability/monitoring	4.10c Brief description of findings, as stated in review.<br><br>[extract or notes; page no / location in report]	

Section no.	Section name	Field header (including instructions) - copied from DE form	Codes/additional guidance
4.11	Strength-based approach	4.11 STRENGTH-BASED APPROACH	
4.11	Strength-based approach	4.11a Did the reviewers look for evidence of whether the studies took a strength based approach in the design/implementation of strategies?<br><br>[Select from list. 'Yes' if there is an explicit mention of 'strength-based' or social and emotional wellbeing (holistic) approaches. Includes explicit mention of connection to country, culture, spirituality, ancestry, family and community, connection to body and mind (NATSIP definition)]	as per 4.7
4.11	Strength-based approach	4.11b If 'yes' to previous, what did the reviewers look for?<br><br>[Note any explicit mention of 'strength-based' or social and emotional wellbeing (holistic) approaches; connection to country, culture, spirituality, ancestry, family and coommunity, connection to body and mind (NATSIP definition)]	as per 4.7
4.11	Strength-based approach	4.11c Brief description of findings, as stated in review.<br><br>[extract or notes; page no / location in report]	
4.12	Cultural respect	4.12 CULTURAL RESPECT	
4.12	Cultural respect	4.12a Did the reviewers look for evidence of whether cultural respect/competence was considered in the design/ implemention of strategies?<br><br>[Select from list; 'Yes' if (1) explicit mention plus description of culturally-tailored resources/messaging in signage etc, (2) measure of cultural competence training, policies, number of Indigenous staff, (3) interventions to improve cultural competence, behaviours, skills and practices in organisation]	as per 4.7
4.12	Cultural respect	4.12b If 'yes' to previous, what did the reviewers look for?<br><br>[Note any of the following: 1) explicit mention plus description of culturally- tailored resources/messaging in signage etc, (2) measure of cultural competence training, policies, number of Indigenous staff, (3) interventions to improve cultural competence, behaviours, skills and practices in organisation]	as per 4.7
4.12	Cultural respect	4.12c Brief description of findings, as stated in review.<br><br>[extract or notes; page no / location in report]	
4.13	Evidence-based	4.13 EVIDENCE-BASED	

Section no.	Section name	Field header (including instructions) - copied from DE form	Codes/additional guidance
4.13	Evidence-based	4.13a Did the reviewers look for evidence of whether existing research was used to inform the design/implementation of the interventions?<br><br>[Select from list. 'Yes' if explicit mention of using existing research to inform design]	as per 4.7
4.13	Evidence-based	4.13b If 'yes' to previous, what did the reviewers look for?<br><br>[Note any explicit mention of using existing research/evidence in designing / implementing the interventions.]	as per 4.7
4.13	Evidence-based	4.13c Brief description of findings, as stated in review.<br><br>[extract or notes; page no / location in report]	
4.14	Human capability/workforce development	4.14 HUMAN CAPABILITY/WORKFORCE DEVELOPMENT	
4.14	Human capability/workforce development	4.14a Did the reviewers look for evidence of investment in human capability/workforce development in the design/implementation of strategies?<br><br>[select from list; 'Yes' if explict mention of investment in human and community capability and supporting choices/workforce development and community governance (NATSIHP)]	as per 4.7
4.14	Human capability/workforce development	4.14b If 'yes' to previous, what did the reviewers look for?<br><br>[Note any mention of investment in human and community capability and supporting choices/workforce development and community governance (NATSIHP)]	as per 4.7
4.14	Human capability/workforce development	4.14c Brief description of findings, as stated in review.<br><br>[extract or notes; page no / location in report]	
4.15	Whole of life	4.15 WHOLE OF LIFE	
4.15	Whole of life	4.15a Did the reviewers look for evidence of whether a whole of life approach was taken when designing/ implementing the strategies?<br><br>[select from list; 'Yes' if explicit mention of lifecourse approaches (specific) [NATSIHP] or the interventions addressing specific life stages (eg early life, adolescents, pregnancy/parenting, midlife, later life)]	as per 4.7

Section no.	Section name	Field header (including instructions) - copied from DE form	Codes/additional guidance
4.15	Whole of life	4.15b If 'yes' to previous, what did the reviewers look for?<br><br>[Note any mention of lifecourse approaches (specific) [NATSIHP] or the interventions addressing specific life stages (eg early life, adolescents, pregnancy/parenting, midlife, later life))	as per 4.7
4.15	Whole of life	4.15c Brief description of findings, as stated in review.<br><br>[extract or notes; page no / location in report]	
4.15	Whole of life	4.7 Notes about the 'how' of the intervention	
5.00	Outcomes	5.0 Outcomes	
5.00	Outcomes	5.0 Description of outcomes considered in the review<br><br>[Brief extract from PICO, aims, etc.]	
5.10	Primary - Smoking cessation	5.1 Primary - Smoking cessation	
5.10	Primary - Smoking cessation	5.1a Smoking cessation - does the review consider this outcome?<br><br>[Select and record details if prompted.	Yes - [outcome name (N=?)]<br>Yes - But no data or studies reported this outcome<br>Unclear - insufficient detail to tell<br>No - Reviewers did not look for this outcome<br>Other [specify]
5.10	Primary - Smoking cessation	5.1b Smoking cessation - how did the reviewers report results for this outcome?<br><br>[select from list; results could be in text, tables, figures]	mainly effect estimates from meta-analysis (or other quant synthesis using explicit methods)<br>mainly effect estimates for single studies (with or w/o CIs)<br>mainly qualitative statements (e.g. increase, no difference)<br>mainly statements about statistical significance/p values (e.g. statistically sig improvement, 'non-significant')<br>a mix: (specify using existing codes)
5.10	Primary - Smoking cessation	5.1c Smoking cessation - what were the results for this outcome?<br><br>[If available, record synthesised data or text summary as stated in review. If no quant synthesis across studies, 'no synthesis' and note main findings. Note details for outcome measure as below OR 'not reported'.]<br>- Time-points reported<br>- Tools used to measure outcomes and whether validated<br>- Population and any subgroup analysis<br>- Important concerns about the quality of the analysis	
5.20	Primary - Prevention of initiation	5.2 Primary - Prevention of initiation	

Section no.	Section name	Field header (including instructions) - copied from DE form	Codes/additional guidance
5.20	Primary - Prevention of initiation	5.2a Prevention of initiation - does the review consider this outcome?<br><br>[Select and record details if prompted.	As per 5.1a
5.20	Primary - Prevention of initiation	5.2b Prevention initiation - how did the reviewers report results for this outcome?<br><br>[select from list; results could be in text, tables, figures]	As per 5.1b
5.20	Primary - Prevention of initiation	5.2c Primary - prevention of initiation - what were the results for this outcome?<br><br>[If available, record synthesised data or text summary as stated in review. If no quant synthesis across studies, 'no synthesis' and note main findings. Note details for outcome measure as below OR 'not reported'.]<br>- Time-points reported<br>- Tools used to measure outcomes and whether validated<br>- Population and any subgroup analysis<br>- Important concerns about the quality of the analysis	
5.30	Primary - Prevention of initiation	5.3 Primary - prevelance reduction	
5.30	Primary - Prevention of initiation	5.3a Prevelance reduction - does the review consider this outcome?<br><br>[Select and record details if prompted.	As per 5.1a
5.30	Primary - Prevention of initiation	5.3b Prevelance reduction - how did the reviewers report results for this outcome?<br><br>[select from list; results could be in text, tables, figures]	As per 5.1b
5.30	Primary - Prevention of initiation	5.3c Prevelance reduction - what were the results for this outcome?<br><br>[If available, record synthesised data or text summary as stated in review. If no quant synthesis across studies, 'no synthesis' and note main findings. Note details for outcome measure as below OR 'not reported'.]<br>- Time-points reported<br>- Tools used to measure outcomes and whether validated<br>- Population and any subgroup analysis<br>- Important concerns about the quality of the analysis	

Section no.	Section name	Field header (including instructions) - copied from DE form	Codes/additional guidance
5.40	Primary - tobacco sales reduction	5.4 Primary - tobacco sales reduction	
5.40	Primary - tobacco sales reduction	5.4a Tobacco sales reduction - does the review consider this outcome?<br><br>[Select and record details if prompted.	As per 5.1a
5.40	Primary - tobacco sales reduction	5.4b Tobacco sales reduction - how did the reviewers report results for this outcome?<br><br>[select from list; results could be in text, tables, figures]	As per 5.1b
5.40	Primary - tobacco sales reduction	5.4c Tobacco sales reduction - what were the results for this outcome?<br><br>[If available, record synthesised data or text summary as stated in review. If no quant synthesis across studies, 'no synthesis' and note main findings. Note details for outcome measure as below OR 'not reported'.]<br>- Time-points reported<br>- Tools used to measure outcomes and whether validated<br>- Population and any subgroup analysis<br>- Important concerns about the quality of the analysis	
5.50	Primary - morbidity/mortality	5.5 Primary - morbidity/mortality	
5.50	Primary - morbidity/mortality	5.5a Morbidity/mortality - does the review consider this outcome?<br><br>[Select and record details if prompted.	As per 5.1a
5.50	Primary - morbidity/mortality	5.5b Morbidity/mortality - how did the reviewers report results for this outcome?<br><br>[select from list; results could be in text, tables, figures]	As per 5.1b
5.50	Primary - morbidity/mortality	5.5c Morbidity/mortality - what were the results for this outcome?<br><br>[If available, record synthesised data or text summary as stated in review. If no quant synthesis across studies, 'no synthesis' and note main findings. Note details for outcome measure as below OR 'not reported'.]<br>- Time-points reported<br>- Tools used to measure outcomes and whether validated<br>- Population and any subgroup analysis<br>- Important concerns about the quality of the analysis	

Section no.	Section name	Field header (including instructions) - copied from DE form	Codes/additional guidance
5.50	Primary - morbidity/mortality	5.6 Notes re. primary outcomes<br><br>[e.g. note if review specifies a primary outcome not included here]	
5.7-5.17	Secondary outcomes	5.7-5.17 Secondary outcomes	
5.7-5.17	Secondary outcomes	5.7 Relapse prevention - does the review consider this outcome?<br><br>[Select and record details if prompted.]	As per 5.1a
5.7-5.17	Secondary outcomes	5.7 Quit attempts - does the review consider this outcome?<br><br>[Select and record details if prompted.]	As per 5.1a
5.7-5.17	Secondary outcomes	5.7 Smoke free homes/workplaces - does the review consider this outcome?<br><br>[Select and record details if prompted.]	As per 5.1a
5.7-5.17	Secondary outcomes	5.7 Cost effectiveness/cost - does the review consider this outcome?<br><br>[Select and record details if prompted.]	As per 5.1a
5.7-5.17	Secondary outcomes	5.8 Knowledge/norms (people, service providers) - does the review consider this outcome?<br><br>[Select and record details if prompted.]	As per 5.1a
5.7-5.17	Secondary outcomes	5.9 Practice change - does the review consider this outcome?<br><br>[Select and record details if prompted.]	As per 5.1a
5.7-5.17	Secondary outcomes	5.10 Human and community capability/workforce development - does the review consider this outcome?<br><br>[Select and record details if prompted.]	As per 5.1a

Section no.	Section name	Field header (including instructions) - copied from DE form	Codes/additional guidance
5.7-5.17	Secondary outcomes	5.11 Adverse effects - does the review consider this outcome?<br><br>[Select and record details if prompted.]	As per 5.1a
5.7-5.17	Secondary outcomes	5.12 Self efficacy/ empowerment/strengths - does the review consider this outcome?<br><br>[Select and record details if prompted.]	As per 5.1a
5.7-5.17	Secondary outcomes	5.13 Equality - does the review consider this outcome?<br><br>[Select and record details if prompted.]	As per 5.1a
5.7-5.17	Secondary outcomes	5.14 Partnership - does the review consider this outcome?<br><br>[Select and record details if prompted.]	As per 5.1a
5.7-5.17	Secondary outcomes	5.15 Engagement - does the review consider this outcome?<br><br>[Select and record details if prompted.]	As per 5.1a
5.7-5.17	Secondary outcomes	5.16 Cultural respect - does the review consider this outcome?<br><br>[Select and record details if prompted.]	As per 5.1a
5.7-5.17	Secondary outcomes	5.17 Notes - re. secondary outcomes<br><br>[e.g. note if review has any of these outcomes as primary outcomes]	As per 5.1a
6.00	AMSTAR & METHODS	6.0 AMSTAR & METHODS	
6.00	AMSTAR & METHODS	6.1 Was an 'a priori' design provided?<br><br>[The research question and inclusion criteria should be established before the conduct of the review. Note: Need to refer to a protocol, ethics approval, or pre-determined/a priori published research objectives to score a “yes.”]	Yes<br>No<br>Can't answer (no information)<br>Can't answer (reviewer uncertain)<br>Not applicable

Section no.	Section name	Field header (including instructions) - copied from DE form	Codes/additional guidance
6.00	AMSTAR & METHODS	6.2 Was there duplicate study selection and data extraction?<br><br>[There should be at least two independent data extractors and a consensus procedure for disagreements should be in place. Note: 2 people do study selection, 2 people do data extraction, consensus process or one person checks the other's work.]	As per 6.1
6.00	AMSTAR & METHODS	6.3 Was a comprehensive literature search performed?<br><br>[At least two electronic sources should be searched. The report must include years and databases used (e.g., Central, EMBASE, and MEDLINE). Key words and/or MESH terms must be stated and where feasible the search strategy should be provided. All searches should be supplemented by consulting current contents, reviews, textbooks, specialized registers, or experts in the particular field of study, and by reviewing the references in the studies found.<br>Note: If at least 2 sources + one supplementary strategy used, select "yes" (Cochrane register/Central counts as 2 sources; a grey literature search counts as supplementary).]	As per 6.1
6.00	AMSTAR & METHODS	6.4 Was the status of publication (i.e. grey literature) used as an inclusion criterion?<br><br>[The authors should state that they searched for reports regardless of their publication type. The authors should state whether or not they excluded any reports (from the systematic review), based on their publication status, language etc.<br>Note: If review indicates that there was a search for "grey literature" or "unpublished literature," indicate "yes." SINGLE database, dissertations, conference proceedings, and trial registries are all considered grey for this purpose. If searching a source that contains both grey and non-grey, must specify that they were searching for grey/unpublished lit.]	As per 6.1
6.00	AMSTAR & METHODS	6.5 Was a list of studies (included and excluded) provided?<br><br>[A list of included and excluded studies should be provided.<br>Note: Acceptable if the excluded studies are referenced. If there is an electronic link to the list but the link is dead, select "no."]	As per 6.1

Section no.	Section name	Field header (including instructions) - copied from DE form	Codes/additional guidance
6.00	AMSTAR & METHODS	6.6 Were the characteristics of the included studies provided?<br><br>[In an aggregated form such as a table, data from the original studies should be provided on the participants, interventions and outcomes. The ranges of characteristics in all the studies analyzed e.g., age, race, sex, relevant socioeconomic data, disease status, duration, severity, or other diseases should be reported.<br>Note: Acceptable if not in table format as long as they are described as above.]	As per 6.1
6.00	AMSTAR & METHODS	6.7 Was the scientific quality of the included studies assessed and documented?<br><br>['A priori' methods of assessment should be provided (e.g., for effectiveness studies if the author(s) chose to include only randomized, double-blind, placebo controlled studies, or allocation concealment as inclusion criteria); for other types of studies alternative items will be relevant.<br>Note: Can include use of a quality scoring tool or checklist, e.g., Jadad scale, risk of bias, sensitivity analysis, etc., or a description of quality items, with some kind of result for EACH study ("low" or "high" is fine, as long as it is clear which studies scored "low" and which scored "high"; a summary score/range for all studies is not acceptable).]	As per 6.1
6.00	AMSTAR & METHODS	6.8 Was the scientific quality of the included studies used appropriately in formulating conclusions?<br><br>[The results of the methodological rigor and scientific quality should be considered in the analysis and the conclusions of the review, and explicitly stated in formulating recommendations.<br>Note: Might say something such as "the results should be interpreted with caution due to poor quality of included studies." Cannot score "yes" for this question if scored "no" for question 7.]	As per 6.1

Section no.	Section name	Field header (including instructions) - copied from DE form	Codes/additional guidance
6.00	AMSTAR & METHODS	6.9 Were the methods used to combine the findings of studies appropriate?<br><br>[For the pooled results, a test should be done to ensure the studies were combinable, to assess their homogeneity (i.e., Chi-squared test for homogeneity, I2). If heterogeneity exists a random effects model should be used and/or the clinical appropriateness of combining should be taken into consideration (i.e., is it sensible to combine?).<br>Note: Indicate "yes" if they mention or describe heterogeneity, i.e., if they explain that they cannot pool because of heterogeneity/variability between interventions.]	As per 6.1
6.00	AMSTAR & METHODS	6.10 Was the likelihood of publication bias assessed?<br><br>[An assessment of publication bias should include a combination of graphical aids (e.g., funnel plot, other available tests) and/or statistical tests (e.g., Egger regression test, Hedges-Olken).<br>Note: If no test values or funnel plot included, score "no". Score "yes" if mentions that publication bias could not be assessed because there were fewer than 10 included studies.]	As per 6.1
6.00	AMSTAR & METHODS	6.11 Was the conflict of interest included?<br><br>[Potential sources of support should be clearly acknowledged in both the systematic review and the included studies.<br>Note: To get a "yes," must indicate source of funding or support for the systematic review AND for each of the included studies.]	As per 6.1
6.00	AMSTAR & METHODS	6.12 Note any important concerns relating to the AMSTAR items	

Section no.	Section name	Field header (including instructions) - copied from DE form	Codes/additional guidance
6.00	AMSTAR & METHODS	6.13 Overall judgement - Low, Moderate or High risk of bias	Score LOW if:<br>**(A)** no important concerns about the following (A1-4) OR the interpretation addressed all concerns:<br>A1. the specification of study eligibility criteria (6.1);<br>A2. methods used to identify and/or select studies (6.2 - study selection, 6.3, 6.4, OR review published prior to ?year?);<br>A3. methods used to collect data and appraise studies (6.2 - data extraction, 6.5, 6.6, 6.7);<br>A4. the synthesis (6.8, 6.9, 6.10, 6.11).<br>**AND**,<br>(B) the relevance of studies to the question was considered (e.g. noting that the evidence came only from non-indigenous populations so different effects may be seen among Indigenous peoples), and<br>(C) the review avoided emphasising results based on statistical significance (i.e. where effects are mixed across important outcomes, the reviewers should mention outcomes for which there was no improvement or a statistically non-significant improvement, in addition to outcomes showing 'statistically signficant' improvement). It is particularly important that this is addressed in any summary of findings (e.g. in the conclusions or abstract) not just the results section.<br><br>Score HIGH if:<br>(A) important concerns about domains 1-4 **WERE NOT** addressed, and/or<br>(B) the relevance of studies to the question **WAS NOT** considered, and/or<br>(C) the review inappropriately emphasised results based on statistical significance.<br><br>ROBIS only rates RoB as LOW or HIGH, so it's tricky to provide an algorithm for MODERATE. I would probably use MODERATE if I wasn't convinced the review was at LOW RoB, but felt a HIGH judgement fails to distinguish 'reasonable reviews' from those where we have major concerns (e.g. reviews that we'd struggle to describe as systematic or that seemed to misrepresent the evidence).
6.00	AMSTAR & METHODS	6.14 Notes re. quality of the review or potential biases	The rating of RoB is VERY judgement based, so important to note why you've made your decision (column 6.14) as we may not be consistent.<br><br>In recording your reasons for a HIGH or MODERATE RoB judgement (column 6.14), you could use the shorthand (A1, A2, A3, and/or A4), (B) and (C) as defined in 6.13.
7.00	Other information	7.0 Other information	
7.00	Other information	7.1 Key conclusions of review authors from abstract or conclusions<br><br>[Extract/summarise main conclusion of the review]	

Section no.	Section name	Field header (including instructions) - copied from DE form	Codes/additional guidance
7.00	Other information	7.2 Correspondence required for further study information<br><br>[what and from whom]<br><br>[My understanding is we won't be doing this now??? Exception might be to write to ask if authors have a protocol or more info re. their methods?? This would enable fair assessment of quality/potential biases.]	
7.00	Other information	7.3 Further study information requested<br><br>[from whom, what and when]	
