# Supplementary material for: Evidence for a comprehensive approach to Aboriginal tobacco control to maintain the decline in smoking: an overview of reviews among Indigenous peoples
Source: Syst Rev. 2017 Jul 10;6:135. doi: 10.1186/s13643-017-0520-9 (PMC5504765; doi:10.1186/s13643-017-0520-9)
Supplement: Supplementary file 3 — Databases searched. [file 13643_2017_520_MOESM3_ESM.doc]

**Additional file 3**: Databases searched

| **Name of database** | **Search strategy** | **Limits** |
| --- | --- | --- |
| 4.0 Medline | As per Supplementary material 3 | ≥2000 |
| 4.1 EMBASE | As per Supplementary material 3 | ≥2000 |
| 4.2 PubMed | As per Supplementary material 3 | ≥2000 |
| 4.3 Turning Research into Practice (TRIPs) | (Tobacco OR smok*) AND (review OR evidence synthesis OR systematic* OR overview OR meta-analysis) AND (Indigen* OR Aborig* OR Torres Strait*) | ≥2000 |
| 4.4 Epistemonikos | (tobacco* OR smok*) AND (review OR evidence synthesis OR systematic* OR overview OR meta-analysis) AND (Indigen* OR Aborig* OR Torres strait*)  Searched title or abstract | ≥2000 |
| 4.5 Centre for Reviews and Dissemination (CRD) | (Tobacco* or smok*) AND (review OR evidence synthesis OR systematic* OR overview OR meta-analysis) AND (Indigen* OR Aborig* OR Torres Strait*) | ≥2000 |
|  | (Tobacco* or smok*) AND (review OR evidence synthesis OR systematic* OR overview OR meta-analysis) AND (Indigen* OR Aborig* OR Torres Strait*) | ≥2000 |
| 4.7 Google Scholar. | Tobacco Indigenous Review  Searched titles until saturation was reached i.e. when additional retrieved results were consistently of no direct relevance. | ≥2000 |
| 4.8 ATSIhealth | (Tobacco* or smok*) AND (review OR evidence synthesis OR systematic* OR overview OR meta-analysis) AND (Indigen* OR Aborig* OR Torres Strait*) | ≥2000 |
| 4.9 PDQ evidence | (title:((tobacco* OR smok*) AND (review OR evidence synthesis OR systematic* OR overview OR meta-analysis) AND (Indigen* OR Aborig* OR Torres strait*)) OR abstract:((tobacco* OR smok*) AND (review OR evidence synthesis OR systematic* OR overview OR meta-analysis) AND (Indigen* OR Aborig* OR Torres strait*))) | ≥2000 |
| 4.10 Healthinfonet | Searched Tobacco and keyword Review and terms in Pubmed =  (((Smoking[mh:noexp] OR tobacco use cessation[mh] OR tobacco use disorder[mh] OR nicotine[mh] OR tobacco smoke pollution[mh]) AND (((australia[mh] OR australia*[tiab]) AND (oceanic ancestry group[mh] OR aborigin*[tiab] OR indigenous[tw])) OR (torres strait* islander*[tiab])) AND medline[sb]) OR ((Smok*[tiab] OR tobacco[tiab] OR nicotine[tiab]) AND (((au[ad] OR australia*[ad] OR australia*[tiab] OR northern territory[tiab] OR northern territory[ad] OR tasmania[tiab] OR tasmania[ad] OR new south wales[tiab] OR new south wales[ad] OR victoria[tiab] OR victoria[ad] OR queensland[tiab] OR queensland[ad]) AND (aborigin*[tiab] OR indigenous[tiab])) OR (torres strait* islander*[tiab])) NOT medline[sb])) AND ( review) | ≥2000 |
| 4.11 AIHW Closing the Gap clearinghouse | Tobacco | ≥2000 |
