# Supplementary material for: Evidence for a comprehensive approach to Aboriginal tobacco control to maintain the decline in smoking: an overview of reviews among Indigenous peoples
Source: Syst Rev. 2017 Jul 10;6:135. doi: 10.1186/s13643-017-0520-9 (PMC5504765; doi:10.1186/s13643-017-0520-9)
Supplement: Supplementary file 4 — Detailed list of search terms. [file 13643_2017_520_MOESM4_ESM.doc]

**Additional file 4**: Detailed list of search terms

| **Search concepts (and terms for website search)** | **MeSH and free text terms for Medline, Embase, PubMed** |
| --- | --- |
| 1. Tobacco AND | exp “Tobacco Use Cessation”/ or exp “Tobacco Use Cessation Products”/  “Tobacco Use Disorder”/  (quit* or abstinen* or abstain or abstent* or cease* or cessation)  (tobacco or nicotine or (smok* and (pipe*1 or cigarette* or e-cigarette* or cigar*1)))  Smoking cessation/, abstinence/ tobacco dependence/ |
| 1. Indigenous AND | Indigen* OR Aborig* OR Torres Strait* OR Islander* OR Oceanic Ancestry Group OR Oceanic OR Inuit OR Maori* OR Saami* OR ((trib* or first) people*) OR ethnic group* OR adivasti OR janajati or Eskimo* or Inuit* or Inuk* or Metis or (First Nation*) or Native* Canadian* or Native* American* or Maori* or Pacific Islander* or American Indian* or Native* Alaska* or Native Hawaiian* or Yupik or Aleut  exp Indigenous people/((tribe* OR tribal OR first) AND people*) |
| 1. Systematic review | Systematic Review search filter was applied (systematic review, meta-analysis, systematic literature review, this systematic review, pooling project, meta synthesis, integrative review, integrative research review, rapid review, consensus development conference, practice guideline, drug class reviews, (cochrane database syst* rev* or acp journal club or health technology assess* or evid* rep* technol* assess* sum* or jbi database system rev* implement rep*)  (clinical guideline and management)  Evidence based  exp Evidence-based Medicine/  review OR evidence synthesis OR systematic* OR overview OR meta-analysis  (NOTNLM OR publisher[sb] OR inprocess[sb] OR pubmednotmedline[sb] OR indatareview[sb] OR pubstatusaheadofprint) AND (systematic[sb])-Pubmed |
