# Supplementary material for: Evidence for a comprehensive approach to Aboriginal tobacco control to maintain the decline in smoking: an overview of reviews among Indigenous peoples
Source: Syst Rev. 2017 Jul 10;6:135. doi: 10.1186/s13643-017-0520-9 (PMC5504765; doi:10.1186/s13643-017-0520-9)
Supplement: Supplementary file 5 — Table of reviews excluded after assessment for eligibility in full text review and reasons for exclusion. [file 13643_2017_520_MOESM5_ESM.doc]

**Additional file 5: Table of reviews excluded after assessment for eligibility in full text review and reasons for exclusion**

| **Excluded review** | **Reason for exclusion** |
| --- | --- |
| Aboriginal Health & Medical Research Council of NSW and NSW Ministry of Health. (2014). The ATRAC Framework: A strategic framework for Aboriginal tobacco resistance and control in NSW. Sydney. | Not a review. |
| Baker, A., Ivers, R. G., Bowman, J., Butler, T., Kay-Lambkin, F. J., Wye, P., . . . Wodak, A. (2006). Where there's smoke, there's fire: high prevalence of smoking among some sub-populations and recommendations for intervention. Drug Alcohol Rev, 25(1), 85-96. doi:10.1080/09595230500459552 | Not specifically Indigenous populations and no description of methods. |
| Bryant, J., Bonevski, B., Paul, C., McElduff, P., & Attia, J. (2011). A systematic review and meta-analysis of the effectiveness of behavioural smoking cessation interventions in selected disadvantaged groups. Addiction, 106(9), 1568-1585. | Not specifically indigenous populations. |
| Carr, S. M., Lhussier, M., Forster, N., Geddes, L., Deane, K., Pennington, M., . . . Hildreth, A. (2011). An evidence synthesis of qualitative and quantitative research on component intervention techniques, effectiveness, cost-effectiveness, equity and acceptability of different versions of health-related lifestyle advisor role in improving health. Health technology assessment (Winchester, England), 15(9), iii-iv, 1-284. doi:10.3310/hta15090 | Not specifically indigenous populations. |
| Carson, K. V., Brinn, M. P., Robertson, T. A., To, A. N. R., Esterman, A. J., Peters, M., & Smith, B. J. (2013). Current and emerging pharmacotherapeutic options for smoking cessation. Substance Abuse: Research and Treatment, 7, 85-105. | Not specifically indigenous populations. |
| Chamberlain, C., O'Mara-Eves, A., Oliver, S., Caird, J. R., Perlen, S. M., Eades, S. J., & Thomas, J. (2013). Psychosocial interventions for supporting women to stop smoking in pregnancy. Cochrane Database of Systematic Reviews, 10, CD001055. | Not specifically indigenous populations. |
| Courtney, R. J., Naicker, S., Shakeshaft, A., Clare, P., Martire, K. A., & Mattick, R. P. (2015). Smoking cessation among low-socioeconomic status and disadvantaged population groups: A systematic review of research output. International Journal of Environmental Research and Public Health, 12(6), 6403-6422. | Not specifically indigenous populations and review focus on study design. |
| Cox, L. S., Okuyemi, K., Choi, W. S., & Ahluwalia, J. S. (2011). A review of tobacco use treatments in U.S. ethnic minority populations. American Journal of Health Promotion, 25(5 Suppl), S11-30. | Not specifically indigenous populations. |
| Doolan, D. M., & Froelicher, E. S. (2006). Efficacy of smoking cessation intervention among special populations: review of the literature from 2000 to 2005. Nursing Research, 55(4 Suppl), S29-37 | Not specifically indigenous populations. |
| Forsetlund, L., Eike, M. C., & Vist, G. E. (2010). Effect of interventions to improve health care services for ethnic minority populations. Norsk Epidemiologi, 20(1), 41-52. | Not specifically indigenous populations. |
| Greaves, L., Johnson, J., Bottorff, J., Kirkland, S., Jategaonkar, N., McGowan, M., . . . Battersby, L. (2006). What are the effects of tobacco policies on vulnerable populations? A better practices review. Canadian journal of public health | Not specifically indigenous populations. |
| Gutmann, L. B., Sobell, L. C., Prevo, M. H., Toll, B. A., Gutwein, C. L., Sobell, M. B., & Hyman, S. M. (2004). Outcome research methodology of smoking cessation trials (1994-1998). Addictive Behaviors, 29(3), 441-463. | Not specifically indigenous populations and studies all prior to 2000. |
| Ivers, R. G. (2004). An evidence-based approach to planning tobacco interventions for Aboriginal people. Drug & Alcohol Review, 23(1), 5-9. | A review of interventions from the general population to inform interventions for Aboriginal people. |
| Ivers, R. G. (2008). Tobacco and aboriginal people in NSW. NSW Public Health Bull, 19(3-4), 65-67. | Not a review. |
| Jeyashree, K., Kathirvel, S., Shewade, H. D., Kaur, H., & Goel, S. (2016). Smoking cessation interventions for pulmonary tuberculosis treatment outcomes. Cochrane Database Syst Rev, 1, Cd011125. doi:10.1002/14651858.CD011125.pub2 | Not indigenous populations |
| Kong, G., Singh, N., & Krishnan-Sarin, S. (2012). A review of culturally targeted/tailored tobacco prevention and cessation interventions for minority adolescents. Nicotine and Tobacco Research, 14(12), 1394-1406. | Not specifically indigenous populations. |
| Lawrence, D., Graber, J. E., Mills, S. L., Meissner, H. I., & Warnecke, R. (2003). Smoking cessation interventions in U.S. racial/ethnic minority populations: an assessment of the literature. Preventive Medicine, 36(2), 204-216. | Not specifically indigenous populations. |
| Liu, J. J., Davidson, E., Bhopal, R. S., White, M., Johnson, M. R. D., Netto, G., . . . Sheikh, A. (2012). Adapting health promotion interventions to meet the needs of ethnic minority groups: Mixed-methods evidence synthesis. Health Technology Assessment, 16(44), 1-469. | Not specifically indigenous populations. |
| Liu, J. J., Wabnitz, C., Davidson, E., Bhopal, R. S., White, M., Johnson, M. R., . . . Sheikh, A. (2013). Smoking cessation interventions for ethnic minority groups--a systematic review of adapted interventions. Preventive Medicine, 57(6), 765-775. | Not specifically indigenous populations. |
| McRobbie, H., Bullen, C., Glover, M., Whittaker, R., Wallace-Bell, M., Fraser, T., & New Zealand Guidelines, G. (2008). New Zealand smoking cessation guidelines. New Zealand Medical Journal, 121(1276), 57-70. | Not specifically indigenous populations. |
| Piper, M. E., Fox, B. J., Welsch, S. K., Fiore, M. C., & Baker, T. B. (2001). Gender and racial/ethnic differences in tobacco-dependence treatment: A commentary and research recommendations. Nicotine and Tobacco Research, 3(4), 291-297 | Not specifically indigenous populations and not a review. |
| Purcell, K. (2015). Evidence review: Addressing the social determinants of inequities in tobacco use. Victoria, Australia: Victorian Health Promotion Foundation. | Not specifically indigenous populations. |
| Ratsch, A., Steadman, K. J., & Bogossian, F. (2010). The pituri story: a review of the historical literature surrounding traditional Australian Aboriginal use of nicotine in Central Australia. J Ethnobiol Ethnomed, 6, 26. | Not a review of interventions. |
| Robertson, J., Stevenson, L., Usher, K., Devine, S., & Clough, A. (2015). A Review of Trends in Indigenous Australian Tobacco Research (From 2004 to 2013), its Associated Outputs and Evidence of Research Translation. Nicotine & Tobacco Research, 17(8), 1039-1048. | Only maps types of smoking research, not study findings. |
| Sherman, E. J., & Primack, B. A. (2009). What works to prevent adolescent smoking? A systematic review of the National Cancer Institute's Research-Tested Intervention Programs. Journal of School Health, 79(9), 391-399. | Not specifically indigenous populations. |
| Small, S., Porr, C., Swab, M., & Murray, C. (2015). The experience and cessation needs of Indigenous women who smoke during pregnancy: A systematic review of qualitative evidence protocol. JBI Database System Rev Implement Rep, 13(4), 51-64. | Protocol only (ongoing review). |
| Stoner, L., Stoner, K. R., Young, J. M., & Fryer, S. (2012). Preventing a Cardiovascular Disease Epidemic among Indigenous Populations through Lifestyle Changes. International Journal of Preventive Medicine, 3(4), 230-240. | Not specifically tobacco review. |
| Thomas, D. P., Davey, M. E., Briggs, V. L., & Borland, R. (2015). Talking About The Smokes: Transforming the evidence to guide Aboriginal and Torres Strait Islander tobacco control. Medical Journal of Australia, 202(10 (Suppl): S3-S4), 92. doi:10.5694/mja15.00464 | Not a review. |
| Twyman, L., Bonevski, B., Paul, C., & Bryant, J. (2014). Perceived barriers to smoking cessation in selected vulnerable groups: A systematic review of the qualitative and quantitative literature. BMJ Open, 4(12), 1-15. | Not specifically indigenous populations. |
| Vidrine, J. I., Cofta-Woerpel, L., Daza, P., Wright, K. L., & Wetter, D. W. (2006). Smoking cessation 2: Behavioral treatments. Behavioral Medicine, 32(3), 99-109. | Not specifically indigenous populations. |
| Vos T., Carter R., Barendregt J., Mihalopoulos C., Veerman J.L., Magnus A., . . . ACE–Prevention Team. (2010). Assessing Cost-Effectiveness in Prevention: (ACE-Prevention): Final report. Brisbane: Centre for Burden of Disease and Cost Effectiveness, School of Population Health, University of Queensland. | Not specifically indigenous populations. |
| Young, C. F., & Skorga, P. (2013). Interventions for tobacco use prevention in indigenous youth. Public Health Nursing, 30(5), 448-450. | Summary of Carson 2012. |
