# Supplementary material for: Evidence for a comprehensive approach to Aboriginal tobacco control to maintain the decline in smoking: an overview of reviews among Indigenous peoples
Source: Syst Rev. 2017 Jul 10;6:135. doi: 10.1186/s13643-017-0520-9 (PMC5504765; doi:10.1186/s13643-017-0520-9)
Supplement: Supplementary file 6 — Detailed AMSTAR rating for each included review. [file 13643_2017_520_MOESM6_ESM.doc]

**Additional file 6**: Detailed AMSTAR rating for each included review

| **Review ID** | **Was an 'a priori' design provided** | **Was there duplicate study selection and data extraction?** | **Was a comprehensive literature search performed?** | **Was the status of publication (i.e. grey literature) used as an inclusion criterion?** | **Was a list of studies (included and excluded) provided?** | **Were the characteristics of the included studies provided?** | **Was the scientific quality of the included studies assessed and documented?** | **Was the scientific quality of the included studies used appropriately in formulating conclusions?** | **Were the methods used to combine the findings of studies appropriate?** | **Was the likelihood of publication bias assessed?** | **Was the conflict of interest included?** | **Overall judgement - Low, Moderate or High risk of bias** |
| --- | --- | --- | --- | --- | --- | --- | --- | --- | --- | --- | --- | --- |
| **Minichiello 2016** | Can't answer (no information) | No - duplicate study selection, but single data extraction and risk of bias assessment (p21) | yes | No - grey literature was included | yes | yes | Yes [overall rating only, not by individual criteria] | Yes [only high and moderate quality studies included in synthesis, although implications of specific designs could have been better incorporated in reported results] | Can't answer (no information) [it is unclear how the authors interpreted finding (appears to be statistical significance along, not direction of effect) or aggregated results (e.g. where there were multiple outcomes within a category or multiple time points) | no | Yes [not explicit but details of funders reported] | Mod |
| **Carson 2014** | Can't answer (no information) | Yes | Yes | No | Yes (the authors listed all relevant included studies (tables), but did not list excluded studies. ) | Yes | Yes | Partial - the risk of bias (quality) of included studies was reported, and some elements of study design were mentioned in reporting results. It is not clear that this was integrated in the conclusions statements about the authors' confidence in the evidence for particular strategies. | Partial - the authors report conducting a 'narrative synthesis'. While the reporting of results study-by-study is appropriate, and includes caveats about limitations of the data reported in some studies (e.g. p4-5), is not clear how the authors aggregated findings across studies or outcomes to draw conclusions about categories of interventions (e.g. mean quit rate reported for 25 cessation studies p8). | No | No | Low |
| **Johnston 2013** | No | No - single screening of titles/abstracts; likely single data extraction | Yes | Yes - grey literature not reviewed (p1330) | Partial - list of included studies, but no list of excluded studies | Yes | Yes | No - the authors assessed quality, but did not integrate this assessment when reporting the results of individual trial and only scant mention was made in drawing conclusions | No - The authors report findings study-by study; they do not described planned methods of analysis or a rationale for not synthesising. It may have been possible to meta-analyse, and this would likely have been a more appropriate synthesis method than study by study reporting, then drawing an overall conclusion across studies without any explicit basis for aggregating findings. | No | Yes-pharmaceutical | Moderate |
| **Carson 2012a (Indigenous populations)** | Yes | No - single person screened/extracted data, data checked by a second | Yes | No - ''enquiries regarding other ... unpublished studies ... were made." | Yes | Yes | Yes | Yes | Yes - meta-analysis and narrative synthesis methods were used. Although there were differences in intervention content, the authors considered clinical and statistical heterogeneity in interpreting their results (discussion p15). | Yes (noted on p 4) "It is possible that due to the nature of these studies some publication bias is occurring with a failure to publish studies that produce no effect".“Providing the inclusion of greater than ten included studies, potential reporting biases would have been assessed using a funnel plot. Asymmetry in the plot could have been attributed to publication bias, but may well be due to true heterogeneity, poor methodological design or artefact. In case of asymmetry, we could have included contour lines corresponding to perceived milestones of statistical significance (p=0.01, 0.05, 0.1etc.) to funnel plots, which may help to differentiate between asymmetry due to publication bias from that due to other factors (Higgins 2009). In instances of fewer than ten studies, the reporting biases were extrapolated within the ’other bias’ section in the risk of bias tables”. (p 8) | Yes | Low |
| **Carson 2015** | Can't answer (no information) | Can't answer (no information) | Can't answer (no information on years searched) | Can't answer (no information) | No | No | Can't answer (no information) | Can't answer (no information) | Can't answer (no information) | Can't answer (no information) | No | high - abstract only |
| **DiGiacomo 2011** | Can't answer (no information) | Yes | Yes | Yes -0nly peer reviewed articles included | Partial - the authors listed all included studies, but did not list excluded studies. | Yes | No, but noted on p 401 "In this review, comparisons of cessation rates and assessments of intervention efficacy were complicated by different study designs, measurement intervals, cessation criteria, and multi-component programs." | No | Yes | No | No | Moderate |
| **CADTH 2013** | Can't answer (no information) | Can't answer (no information) | No - multiple databases (only one for primary studies), but limited to 10 years and no search terms reported. | No | Partial - the authors listed all relevant included literature (although no primary studies were identified), but did not list excluded studies. | Yes - the authors reported on one review, which did not identify any relevant primary studies | No - the authors did not assess the RoB of the included systematic review | n/a - there was no evidence from which to draw conclusions | n/a - there was no evidence to synthesise | No | No | Moderate |
| **Gould 2013a** | Can't answer (no information) | Partial - two authors screened independently, one data extracted and a second checked | Yes | No | Partial - all included studies are listed, but excluded are not | Yes | Yes | Yes - quality was tabulated and reported in a separate section, and not integrated with reporting of effects, but the conclusions appropriately reflect study quality | Yes | No | Yes | Low |
| **Passey 2013** | No | Can't answer (no information) | Yes | Yes only peer-reviewed papers were included. | Partial - list of included studies, but excluded studies not listed | No quality rating only | Yes | Yes - but there is very limited reporting of the results of the trials (no effect estimates, not reporting of outcomes measured) | No - the authors do not provide adequate data for the reader to intepret the effects of the interventions in either of the studies (no effect estimates or any quantitative data, no mention of outcomes measured) | No | Yes | High |
| **Carson 2012b** | Yes | No - single person screened/extracted data, data checked by a second | Yes | No - ''enquiries regarding other ... unpublished studies ... were made." | Yes | Yes | Yes | Yes | Yes - narrative synthesis seemed appropriate | Can't answer (no information) Limited studies. | Yes | low |
| **Carson 2013** | Can't answer (no information) | Can't answer (no information) | Can't answer (no information) | Can't answer (no information) | No | No | Can't answer (no information) | Can't answer (no information) | Can't answer (no information) | Can't answer (no information) | Yes | high - abstract only |
| **Ivers 2003** | Can't answer (no information) | No | Yes | Yes | Can't answer (no information) | Yes | Yes but NHMRC levels only | Yes | Not applicable | Not applicable | Yes | Moderate |
| **Ivers 2011** | No | Can't answer (no information) | Can't answer (no information) | Yes | No | No | Can't answer (no information) | Can't answer (no information) | Yes | No | No | High |
| **Ivers 2014** | Can't answer (no information) | Can't answer (no information) | Can't answer (no information) | Can't answer (no information) | No | No | Can't answer (no information) | Can't answer (no information) | Can't answer (no information) | No | No | High |
| **Power 2009** | Yes | No | Yes | No - grey literature was eligible for inclusion | Partial - included studies were listed, but excluded studies were not | Yes | No - study design was described and categorised according to NHMRC levels of evidence, but the study methods were not appraised to assess potential risk of bias | Yes - the authors reported NHMRC levels of evidence for each of the results they presented, however the risk of bias was not assessed beyond categorising studies by study design | Yes - The authors do not report their planned methods for synthesising across studies. Results are reported study-by-study except for the one intervention where there is more than two studies. It is not clear how the authors aggregated results across these two studies for formulate their conclusion. | No | No | Moderate |
| **Upton 2014** | Can't answer (no information) | Can't answer (no information) | Yes | No | No | No | No - study design was categorised according to NHMRC levels of evidence (although not reported), but study methods were not appraised to assess potential risk of bias | No - information about study quality was not integrated into the reporting of results or conclusions | No - the authors did not describe their methods for synthesis, and it is unclear how they aggregated results across outcomes and studies to formulate their conclusions. | No | No | High |
| **Clifford 2011** | Can't answer (no information) | Yes | Yes | Yes | Can't answer (reviewer uncertain) Reasons for exclusion noted in flowchart only. | Yes | Can't answer (reviewer uncertain) - methodological characteristics only | Can't answer (reviewer uncertain) Noted discusses limitations of research. | Yes | No | No | Moderate |
| **Brusse 2014** | Can't answer (no information) | Can't answer (no information) | No - One database; search 2011 to Nov 2013 p4 | No | Partial - tables reporting the included studies are provided on p13-17 of full pdf but not list of excluded studies | Yes - table of included studies is provided on p13-17 of full pdf | No | No - the scientific quality of studies (risk of bias) was not assessed, therefore was not used in formulating conclusions | Yes - the authors tabulate results study-by-study, coding overall findings from each study as positive, mixed or negative. They include a brief description of the results. They do not report the method used to code, but based their coding and description of results, it is likely they have vote counted results across outcomes based on statistical significance (not the recommended approach. It is unclear whether the text describes selected outcomes, so there is risk of selective reporting. While the synthesis is imperfect, the conclusions are cautious and overall, the methods are adequate.] | No | Yes | Moderate |
| **Gould 2013b** | No | Yes | Yes | No | Yes - all included studies are listed, but excluded are not | Yes | Yes | Yes | Yes | Not applicable-qualitative | Yes | Low ROB |
| **Thompson 2011** | Can't answer (no information) | Can't answer - two authors 'examined' papers, but unclear if independent screening and/or data extraction | Partial - multiple databases, search terms reported, but no dates | No - grey literature was included | Partial - included studies were listed, but excluded studies were not | Yes | No - no methods reported for systematic assessment of risk of bias or quality, but limitations of each study are noted in Table 1. | Yes - although not reported systematically, the authors incorporate information about the limitations of study quality in reporting results and formulating conclusions | Yes - not synthesis methods are reported, but results are mainly reported study by study | No | Yes | moderate |
| **Clifford 2009** | Can't answer (no information) | Can't answer (no information) | Yes | No | Yes (flowchart only with reasons for exclusion) | Yes | No | Can't answer (reviewer uncertain) | Can't answer (reviewer uncertain) | Not applicable | Can't answer (no information) | Moderate risk of bias |
