# Supplementary material for: Evidence for a comprehensive approach to Aboriginal tobacco control to maintain the decline in smoking: an overview of reviews among Indigenous peoples
Source: Syst Rev. 2017 Jul 10;6:135. doi: 10.1186/s13643-017-0520-9 (PMC5504765; doi:10.1186/s13643-017-0520-9)
Supplement: Supplementary file 7 — Matrix of references to studies included in reviews included in the Overview. [file 13643_2017_520_MOESM7_ESM.pdf]

Additional file 7: Matrix of references to studies included in reviews included in the Overview

ID for reference		Program name (A-Z)	**Brusse 2014** JMIR (4)	**CADTH 2013** Rapid Review (0)	**Carson 2012a** CDSR (prevention) (2)	**Carson 2012b** CDSR (cessation) (4)	**Carson 2013** Respirology (6)*	**Carson 2015** Respirology (9)*	**Carson 2014** Evidence Base (91)	**Clifford 2009** Hlth Prom Int (2)	**Clifford 2011** ANZ J Pub Hlth (4)	**DiGiacomo 2011** Int J Environ Res Pub Hlth (9)	**Gould 2013a** Tobacco Control (21)	**Gould 2013b** Nicotine & Tobacco Cont (7)	**Ivers 2003** ANZ J Pub Hlth (4)	**Ivers 2011** Closing the Gap (11)**	**Ivers 2014** NSW framework (11)**	**Johnston 2013** Nicotine & Tobacco Res (5)	**Minichiello 2016** BMC Public Health (85)***	**Passey 2013** MJA (2)	**Power 2009** Hlth Prom J Aus (14)	**Thompson 2011** ANZ J Pub Hlth (11)	**Upton 2014** Rapid Review DoH (31)**	No. of reviews including this study
**Studies in Australia: Aboriginal and Torres Strait Islander peoples**																								
1	Finlay 2013	Aboriginal tobacco resistance kit							1															1
2	Maddox 2013	ACT Aboriginal and Torres Strait Islander Tobacco Control Strategy							1															1
3	Tasmanian Aboriginal Centre Inc 2014	Alcohol, tobacco and other drugs program							1															1
4	Shah 2014	Asthma and smoking prevention project							1															1
5	Lewis 2006	Beyond the big smoke							1															1
6	Australian Government Department of Health 2011	Break the Chain mass media campaign							1										1					2
7	Boyle 2010	Bubblewrap campaign											1						1					2
8	Cancer Council Western Australia 2009	Bubblewrap campaign															1							1
9	Davison 2014	Butt busters							1															1
10	Young 2007	BUTT OUT							1										1		1			3
11	Drug and Alcohol Services South Australia 2014	Ceduna Day Centre							1															1
12	Sarin 2014	Clean air dreaming							1															1
13	Clifford 2014	Deadly choices																					1	1
14†	Institute for Urban Indigenous Health 2014	Deadly choices																	1				1	2
15	Malseed 2014a	Deadly choices																	1					1
16	Malseed 2014b	Deadly choices																					1	1
17	Malseed 2013	Deadly choices																	1					1

			**Reviews** (number of references to included studies)																					
**ID for reference**		**Program name (A-Z)**	**Brusse 2014** JMIR (4)	**CADTH 2013** Rapid Review (0)	**Carson 2012a** CDSR (prevention) (2)	**Carson 2012b** CDSR (cessation) (4)	**Carson 2013** Respirology (6)*	**Carson 2015** Respirology (9)*	**Carson 2014** Evidence Base (91)	**Clifford 2009** Hlth Prom Int (2)	**Clifford 2011** ANZ J Pub Hlth (4)	**DiGiacomo 2011** Int J Environ Res Pub Hlth (9)	**Gould 2013a** Tobacco Control (21)	**Gould 2013b** Nicotine & Tobacco Cont (7)	**Ivers 2003** ANZ J Pub Hlth (4)	**Ivers 2011** Closing the Gap (11)**	**Ivers 2014** NSW framework (11)**	**Johnston 2013** Nicotine & Tobacco Res (5)	**Minicheiello 2016** BMC Public Health (85)***	**Passey 2013** MJA (2)	**Power 2009** Hlth Prom J Aus (14)	**Thompson 2011** ANZ J Pub Hlth (11)	**Upton 2014** Rapid Review DoH (31)**	No. of reviews including this study
18	Day 2007	Deadly nungas say not to puiya!							1															1
19	Kickett 2009	Drug and Alcohol Awarness							1															1
20	Minniecon 2005	Engaging an Aboriginal Elder in promoting tobacco control messages to the Aboriginal & Torres Strait Islander community project							1															1
21	Cancer Council South Australia 2014	Enhancement campaign: South Australian and Northern Territory Quitlines							1															1
22	Gould 2009	Give up the smokes (GUTS)							1															1
23	Gould 2006	Give up the smokes (GUTS) program							1															1
24	Dean 2010	Gnumaries hurt program							1															1
25	Carson 2012	Improving health for Aboriginal people through tobacco related research							1															1
26	Bailie 2013	Indigenous Chronic Disease Package																					1	1
27	KPMG 2013b	Indigenous Chronic Disease Package																					1	1
28	KPMG 2013a	Indigenous Chronic Disease Package																					1	1
29	Cargo 2011	Indigenous Healthy Lifestyles Project (IHLP)																					1	1

ID for reference		Program name (A-Z)	Reviews (number of references to included studies)																					No. of reviews including this study
			**Brusse 2014** JMIR (4)	**CADTH 2013** Rapid Review (0)	**Carson 2012a** CDSR (prevention) (2)	**Carson 2012b** CDSR (cessation) (4)	**Carson 2013** Respirology (6)*	**Carson 2015** Respirology (9)*	**Carson 2014** Evidence Base (91)	**Clifford 2009** Hlth Prom Int (2)	**Clifford 2011** ANZ J Pub Hlth (4)	**DiGiacomo 2011** Int J Environ Res Pub Hlth (9)	**Gould 2013a** Tobacco Control (21)	**Gould 2013b** Nicotine & Tobacco Cont (7)	**Ivers 2003** ANZ J Pub Hlth (4)	**Ivers 2011** Closing the Gap (11)**	**Ivers 2014** NSW framework (11)**	**Johnston 2013** Nicotine & Tobacco Res (5)	**Minicheiello 2016** BMC Public Health (85)***	**Passey 2013** MJA (2)	**Power 2009** Hlth Prom J Aus (14)	**Thompson 2011** ANZ J Pub Hlth (11)	**Upton 2014** Rapid Review DoH (31)**	
30	Larson 2010	Indigenous Healthy Lifestyles Project (IHLP)																					1	1
31	Harvey 2002	Indigenous Smoke Free Project								1					1	1	1		1		1	1		7
32	Murphy 2009	Indigenous women's project							1															1
33	Hippi 2009	I-Quitt							1															1
34	Griffiths 2009	Justice health quit smoking project							1															1
35	Martinez 2013	Keep Koori kids smoke free							1															1
36	Aboriginal Health and Medical Research Council of New South Wales 2010	Kick the habit social marketing campaign							1															1
37†	Adams 2005	Koori Tobacco Cessation Project																	1					1
38	Mark 2005	Koori Tobacco Cessation Project																				1		1
39	Mark 2004	Koori Tobacco Cessation Project									1	1				1					1	1	1	6
40	Chapman 2000	Make smoking history							1															1
41	Burgess 2008	Maningrida 'Smoke-busters' campaign																	1					1
42	Johnston 1998	Maningrida 'Be Smoke Free' Project				1		1	1		1		1		1				1				1	8
43	Thomas 2007	Monitoring and evaluating Aboriginal tobacco control							1															1
44†	Institute for Urban Indigenous Health 2014	Murri Places Smoke-free Spaces							1										1					2
45	Dimer 2004	My heart my family our culture							1															1

ID for reference		Program name (A-Z)	**Reviews** (number of references to included studies)																					
			**Brusse 2014** JMIR (4)	**CADTH 2013** Rapid Review (0)	**Carson 2012a** CDSR (prevention) (2)	**Carson 2012b** CDSR (cessation) (4)	**Carson 2013** Respirology (6)*	**Carson 2015** Respirology (9)*	**Carson 2014** Evidence Base (91)	**Clifford 2009** Hlth Prom Int (2)	**Clifford 2011** ANZ J Pub Hlth (4)	**DiGiacomo 2011** Int J Environ Res Pub Hlth (9)	**Gould 2013a** Tobacco Control (21)	**Gould 2013b** Nicotine & Tobacco Cont (7)	**Ivers 2003** ANZ J Pub Hlth (4)	**Ivers 2011** Closing the Gap (11)**	**Ivers 2014** NSW framework (11)**	**Johnston 2013** Nicotine & Tobacco Res (5)	**Minicheiello 2016** BMC Public Health (85)***	**Passey 2013** MJA (2)	**Power 2009** Hlth Prom J Aus (14)	**Thompson 2011** ANZ J Pub Hlth (11)	**Upton 2014** Rapid Review DoH (31)**	No. of reviews including this study
46	Ell 2013	National Tobacco Campaign																					1	1
47	National Tobacco Campaign 1999	National Tobacco Campaign													1									1
48	ORIMA 2013	National Tobacco Campaign (Quit for You, Quit for Two)																					1	1
49†	Adams 2005	No more Bundah, Winnunga Nimmityjah,																	1					1
50	Webb 2014	No more Bundah, Winnunga Nimmityjah,							1															1
51	Bell 2012	No Smokes Project																	1					1
52	Gould 2010	No Smokes Project, North coast							1															1
53	Adams 2006	Not reported in review																				1		1
54	Andrews 1997	Not reported in review																				1		1
55	Bardsley 1999	Not reported in review														1								1
56	DiGiacomo 2007	Not reported in review							1			1							1		1	1	1	6
57	Douglas 2001	Not reported in review												1										1
58	Eades 2012	Not reported in review							1								1		1	1			1	5
59	Fairer Health Victoria 2009	Not reported in review																					1	1
60	Fletcher 2011	Not reported in review																					1	1
61	Gilligan 2009	Not reported in review												1										1
62	Gilligan 2008	Not reported in review																	1					1
63	Gray 2002	Not reported in review																			1		1	2
64	Heath 2006	Not reported in review												1										1
65	Ivers 2003	Not reported in review				1		1	1		1	1				1	1		1		1		1	10
66	Johnston 2008	Not reported in review												1		1								2
67	Johnston 2010	Not reported in review											1			1							1	3
68	Lindorff 2002	Not reported in review																				1		1
69	Panaretto 2010	Not reported in review																					1	1

ID for reference		Program name (A-Z)	**Reviews** (number of references to included studies)																						
			**Brusse 2014** JMIR (4)	**CADTH 2013** Rapid Review (0)	**Carson 2012a** CDSR (prevention) (2)	**Carson 2012b** CDSR (cessation) (4)	**Carson 2013** Respirology (6)*	**Carson 2015** Respirology (9)*	**Carson 2014** Evidence Base (91)	**Clifford 2009** Hlth Prom Int (2)	**Clifford 2011** ANZ J Pub Hlth (4)	**DiGiacomo 2011** Int J Environ Res Pub Hlth (9)	**Gould 2013a** Tobacco Control (21)	**Gould 2013b** Nicotine & Tobacco Cont (7)	**Ivers 2003** ANZ J Pub Hlth (4)	**Ivers 2011** Closing the Gap (11)**	**Ivers 2014** NSW framework (11)**	**Johnston 2013** Nicotine & Tobacco Res (5)	**Minicheiello 2016** BMC Public Health (85)***	**Passey 2013** MJA (2)	**Power 2009** Hlth Prom J Aus (14)	**Thompson 2011** ANZ J Pub Hlth (11)	**Upton 2014** Rapid Review DoH (31)**	No. of reviews including this study
70	Pilkington 2009	Not reported in review																				1		1
71	Stewart 2011	Not reported in review											1											1
72	Thomas 2013	Not reported in review											0						1				1	2
73	Wakefield 2008	Not reported in review															1							1
74	West 1998	Not reported in review																				1		1
75	Wilson 2009	Not reported in review												1										1
76	Wood 2008	Not reported in review												1								1		2
77	Gussy 2010	Our space smoke free							1															1
78	Gould 2012	Pregnant Aboriginal and Torres Strait Islander RCT																	1					1
79	Aboriginal Health and Medical Research Council of New South Wales 2011	Quitline enhancement project NSW															1							1
80	Ford 2005	Reducing risk of SIDS in Aboriginal communities							1															1
81	Chamberlain 2008	Reducing smoking amongst pregnant Aboriginal women in Victoria: an holistic approach							1															1
82	Coole 2010	Regional tackling smoking and healthy lifestyle workforce and activities							1															1
83	Gentle 2008	Remote Aboriginal tobacco project							1															1
84	Nunkuwarrin Yunti of South Australia Inc 2013	Rewrite your story campaign							1															1
85	Yarran 2010	Rockingham and Kwinana tobacco control project							1															1

ID for reference		Program name (A-Z)	**Reviews** (number of references to included studies)																					
			**Brusse 2014** JMIR (4)	**CADTH 2013** Rapid Review (0)	**Carson 2012a** CDSR (prevention) (2)	**Carson 2012b** CDSR (cessation) (4)	**Carson 2013** Respirology (6)*	**Carson 2015** Respirology (9)*	**Carson 2014** Evidence Base (91)	**Clifford 2009** Hlth Prom Int (2)	**Clifford 2011** ANZ J Pub Hlth (4)	**DiGiacomo 2011** Int J Environ Res Pub Hlth (9)	**Gould 2013a** Tobacco Control (21)	**Gould 2013b** Nicotine & Tobacco Cont (7)	**Ivers 2003** ANZ J Pub Hlth (4)	**Ivers 2011** Closing the Gap (11)**	**Ivers 2014** NSW framework (11)**	**Johnston 2013** Nicotine & Tobacco Res (5)	**Minicheiello 2016** BMC Public Health (85)***	**Passey 2013** MJA (2)	**Power 2009** Hlth Prom J Aus (14)	**Thompson 2011** ANZ J Pub Hlth (11)	**Upton 2014** Rapid Review DoH (31)**	No. of reviews including this study
86†	Adams 2005	Rumbalara Football Netball Club, Tobacco Project																	1					1
87	Adams 2006	Rumbalara Football Netball Club, Tobacco Project										1				1	1				1		1	5
88	Rumbalara Aboriginal Co-operative 2012	Rumbalara Football Netball Club, Tobacco Project							1															1
89	Healthway Western Australian State Government 2002	Say No to smokes project							1															1
90	Bond 2012	Smoke free life							1															1
91	Carroll 2006	SmokeCheck NSW							1							1								2
92	Hearn 2011	SmokeCheck NSW							1								1						1	3
93	Jenkinson 2007	SmokeCheck NT							1															1
94	Queensland Health 2007	SmokeCheck QLD																			1	1	1	3
95	Campbell 2014	SmokeCheck QLD [Northern Queensland Indigenous Tobacco Project]							1										1				1	3
96	Aboriginal Health Council of SA 2002	SmokeCheck SA (Puyu Wiya)																			1			1
97	Stewart 2011	SmokeCheck SA (Puyu Wiya)							1				0											1
98	Quit SA 2011	Smoke-free pregnancy project - Aboriginal women and their families							1															1
99	Lynch 2014	Smokers program							1															1
100	Ryan 2010	Smoking no good aye							1															1

ID for reference		Program name (A-Z)	Reviews (number of references to included studies)																						No. of reviews including this study
			**Brusse 2014** JMIR (4)	**CADTH 2013** Rapid Review (0)	**Carson 2012a** CDSR (prevention) (2)	**Carson 2012b** CDSR (cessation) (4)	**Carson 2013** Respirology (6)*	**Carson 2015** Respirology (9)*	**Carson 2014** Evidence Base (91)	**Clifford 2009** Hlth Prom Int (2)	**Clifford 2011** ANZ J Pub Hlth (4)	**DiGiacomo 2011** Int J Environ Res Pub Hlth (9)	**Gould 2013a** Tobacco Control (21)	**Gould 2013b** Nicotine & Tobacco Cont (7)	**Ivers 2003** ANZ J Pub Hlth (4)	**Ivers 2011** Closing the Gap (11)**	**Ivers 2014** NSW framework (11)**	**Johnston 2013** Nicotine & Tobacco Res (5)	**Minicheiello 2016** BMC Public Health (85)***	**Passey 2013** MJA (2)	**Power 2009** Hlth Prom J Aus (14)	**Thompson 2011** ANZ J Pub Hlth (11)	**Upton 2014** Rapid Review DoH (31)**	
101	Daniel 2008	Smoking reduction strategy development and intervention among Aboriginal health workers							1															1
102	Cosh 2013	South Australian Quitline							1										1					2
103	Johnston 2013	Starting to smoke							1															1
104	Passey 2009a	Stop smoking in its tracks							1					1										2
105	Passey 2009b	Stop smoking in its tracks							1															1
106	Thomas 2014	Talking about the smokes																					1	1
107	Boadle 2006	Tasmanian Aboriginal tobacco control project							1															1
108	Marley 2014a	The Be Our Ally Beat Smoking (BOABS) study							1								1		1				1	4
109	Marley 2014b	The Be Our Ally Beat Smoking (BOABS) study																	1					1
110	Ivers 2005a	The Tobacco Action Project							1										1		1			3
111	Ivers 2006a	The Tobacco Action Project														1	1		1		1		1	5
112	Ivers 2005b	The Tobacco Project									1		1				1		1		1		1	6
113	Ivers 2006b	The Tobacco Project														1			1		1			3
114	Thomas 2010	The Tobacco Project											0						1				1	2
1115	Walley 1998	The Western Australian Aboriginal smoking project								1														1
116	Seibold 2000	Tobacco Control Pilot Project													1	1								2

ID for reference		Program name (A-Z)	Reviews (number of references to included studies)																					
			**Brusse 2014** JMIR (4)	**CADTH 2013** Rapid Review (0)	**Carson 2012a** CDSR (prevention) (2)	**Carson 2012b** CDSR (cessation) (4)	**Carson 2013** Respirology (6)*	**Carson 2015** Respirology (9)*	**Carson 2014** Evidence Base (91)	**Clifford 2009** Hlth Prom Int (2)	**Clifford 2011** ANZ J Pub Hlth (4)	**DiGiacomo 2011** Int J Environ Res Pub Hlth (9)	**Gould 2013a** Tobacco Control (21)	**Gould 2013b** Nicotine & Tobacco Cont (7)	**Ivers 2003** ANZ J Pub Hlth (4)	**Ivers 2011** Closing the Gap (11)**	**Ivers 2014** NSW framework (11)**	**Johnston 2013** Nicotine & Tobacco Res (5)	**Minicheiello 2016** BMC Public Health (85)***	**Passey 2013** MJA (2)	**Power 2009** Hlth Prom J Aus (14)	**Thompson 2011** ANZ J Pub Hlth (11)	**Upton 2014** Rapid Review DoH (31)**	No. of reviews including this study
117	Robertson 2013	Top End Tobacco Project																	1				1	2
118†	Robertson 2007a	Top End Tobacco Project							1															1
119	Robertson 2007b	Top End Tobacco Project																			1			1
120	Government of Victoria 2014	Victorian Closing the Gap in Health Outcomes Initiative																	1					1
121	Read 2012	WA prisons smoking reducation plan							1															1
122	Davis 2013	Yarning it up							1															1
123	Ramamoorthi 2009	Healthy starts (Te Piripohotanga)							1															1
124	Walker 2015	Not reported in review																	1					1
**Studies in Canada: Aboriginal/First Nations people, Métis people, Inuit people, "non-status people"**																								
1	Mashford-Pringle 2012	Aboriginal Head Start Urban and Northern Communities Program																	1					1
2	Mashford-Pringle 2008	Aboriginal Head Start Urban and Northern Communities Program																	1					1
3	Mitchell 2007	Aniqsaattiarniq – Breathing Easy																	1					1
4†	Aboriginal Cancer Care Unit 2008	Anishnawbe Mushkiki – Sema Kenjigewin Aboriginal Tobacco Misuse Program																	1					1
5	Inuit Tapiriit Kanatami 2011	Blue Light Campaign																	1					1
6	Hayward 2007	Canadian Quitlines							1			1							1					3

			**Reviews** (number of references to included studies)																					
**ID for reference**		**Program name (A-Z)**	**Brusse 2014** JMIR (4)	**CADTH 2013** Rapid Review (0)	**Carson 2012a** CDSR (prevention) (2)	**Carson 2012b** CDSR (cessation) (4)	**Carson 2013** Respirology (6)*	**Carson 2015** Respirology (9)*	**Carson 2014** Evidence Base (91)	**Clifford 2009** Hlth Prom Int (2)	**Clifford 2011** ANZ J Pub Hlth (4)	**DiGiacomo 2011** Int J Environ Res Pub Hlth (9)	**Gould 2013a** Tobacco Control (21)	**Gould 2013b** Nicotine & Tobacco Cont (7)	**Ivers 2003** ANZ J Pub Hlth (4)	**Ivers 2011** Closing the Gap (11)**	**Ivers 2014** NSW framework (11)**	**Johnston 2013** Nicotine & Tobacco Res (5)	**Minicheiello 2016** BMC Public Health (85)***	**Passey 2013** MJA (2)	**Power 2009** Hlth Prom J Aus (14)	**Thompson 2011** ANZ J Pub Hlth (11)	**Upton 2014** Rapid Review DoH (31)**	No. of reviews including this study
7†	Wesche 2011	Changing the Culture of Smoking – A Community-Based Participatory Research Project, Inuvialuit Region																	1					1
8	Baydala 2009	Life skills training (LST) program							1															1
9	Irfan 2012	Making Aboriginal Kids Walk Away (From Tobacco Abuse) (MAKWA)																	1					1
10†	Wesche 2011	Métis Nation British Columbia's Aboriginal ActNow BC Program																	1					1
11	McKennitt 2012	Not reported in review							1										1					2
12†	Wesche 2011	Peterborough County-City Health's "Choose to be SmokeFree" Tobacco Cessation Program																	1					1
13†	Aboriginal Cancer Care Unit 2008	Sacred smoke																	1					1
14	Chansonneuve 2007	Sacred smoke																	1					1
15	Inuit Tobacco Free Network 2011	Smoke Stories: Quit Clips by Inuit Youth — Video Screening Contest																	1					1
16†	Wesche 2011	Stay Quit to Win Challenge – Tobacco Control, Nunavik																	1					1
17	Irfan 2012	Youth Action Alliance of Manitoulin Island																	1					1

			**Reviews** (number of references to included studies)																					
**ID for reference**		**Program name (A-Z)**	**Brusse 2014** JMIR (4)	**CADTH 2013** Rapid Review (0)	**Carson 2012a** CDSR (prevention) (2)	**Carson 2012b** CDSR (cessation) (4)	**Carson 2013** Respirology (6)*	**Carson 2015** Respirology (9)*	**Carson 2014** Evidence Base (91)	**Clifford 2009** Hlth Prom Int (2)	**Clifford 2011** ANZ J Pub Hlth (4)	**DiGiacomo 2011** Int J Environ Res Pub Hlth (9)	**Gould 2013a** Tobacco Control (21)	**Gould 2013b** Nicotine & Tobacco Cont (7)	**Ivers 2003** ANZ J Pub Hlth (4)	**Ivers 2011** Closing the Gap (11)**	**Ivers 2014** NSW framework (11)**	**Johnston 2013** Nicotine & Tobacco Res (5)	**Minicheiello 2016** BMC Public Health (85)***	**Passey 2013** MJA (2)	**Power 2009** Hlth Prom J Aus (14)	**Thompson 2011** ANZ J Pub Hlth (11)	**Upton 2014** Rapid Review DoH (31)**	No. of reviews including this study
**Studies in Fiji: ethnic Fijians**																								
1	Groth-Marnat 1996	Not reported in review																	1					1
**Studies in New Zealand: Māori people, Pacific Islanders (10 of the 19 studies included non-Indigenous people)**																								
1	Maddison 2014	Fit2Quit intervention							1															1
2	Fernandez 2008	It's about whānau (IAW) mass media campaign, plus New Zealand Quitline referral											1											1
3	Grigg 2008	It's about whānau (IAW) mass media campaign, plus New Zealand Quitline referral							1				1						1					3
4	Wilson 2005	It's about whānau (IAW) mass media campaign, plus New Zealand Quitline referral											1						1					2
5	Glover 2009	Keeping Kids Smokefree							1															1
6	Wilson 2010	New Zealand Quitline											1											1
7	Bullen 2010	New Zealand Quitline plus nicotine patches							1									1						2
8	Watson 2011	New Zealand's Smoke-free Environments Amendment Act 2003 (SFEAA)																	1					1
9	Cowie 2014	Not reported in review																	1					1
10	Glover 2014	Not reported in review																	1					1
11	Holt 2005	Not reported in review				1		1	1			1							1					5
12	Walker 2011	Not reported in review																1						1
13	Whittaker 2011	Not reported in review	1						1				1					1						4
14	Whittaker 2008	Not reported in review	1										1											2

ID for reference		Program name (A-Z)	Reviews (number of references to included studies)																					
			**Brusse 2014** JMIR (4)	**CADTH 2013** Rapid Review (0)	**Carson 2012a** CDSR (prevention) (2)	**Carson 2012b** CDSR (cessation) (4)	**Carson 2013** Respirology (6)*	**Carson 2015** Respirology (9)*	**Carson 2014** Evidence Base (91)	**Clifford 2009** Hlth Prom Int (2)	**Clifford 2011** ANZ J Pub Hlth (4)	**DiGiacomo 2011** Int J Environ Res Pub Hlth (9)	**Gould 2013a** Tobacco Control (21)	**Gould 2013b** Nicotine & Tobacco Cont (7)	**Ivers 2003** ANZ J Pub Hlth (4)	**Ivers 2011** Closing the Gap (11)**	**Ivers 2014** NSW framework (11)**	**Johnston 2013** Nicotine & Tobacco Res (5)	**Minicheiello 2016** BMC Public Health (85)***	**Passey 2013** MJA (2)	**Power 2009** Hlth Prom J Aus (14)	**Thompson 2011** ANZ J Pub Hlth (11)	**Upton 2014** Rapid Review DoH (31)**	No. of reviews including this study
15	Hiscock 2009	PEGS: Preparation, Education, Giving up and Staying Smoke Free Programme																	1					1
16	Walker 012	Quitline NZ plus very low nicotine cigarettes							1									1						2
17	Bramley 2005	STOMP: Stop Smoking by Mobile Phone	1			1		1	1				1					1	1					7
18	Rogers 2005	STOMP: Stop Smoking by Mobile Phone	1																					1
19	Glover 2014	WERO study							1															1
**Studies in Taiwan**																								
1	Lin 2013	Not reported in review																	1					1
**Studies in the USA: Alaska Native, American Indian people, Native American people, Yup'ik people, Cup'ik people, Native Hawaiian, Pacific Islanders**																								
1	Patten 2010	Alaska Native Pregnant Women intervention							1				1						1	1				4
2	Patten 2012	Alaska Native Pregnant Women intervention											0						1					1
3	Boles 2009	Alaska Quitline							1			1							1					3
4	Choi 2011	All Nations Breath of Life							1				0											1
5	Daley 2009	All Nations Breath of Life											1											1
6	Makosky 2010	All Nations Breath of Life							1										1					2
7	Horn 2005	American Indian Not on Tobacco (N-O-T) program							1										1					2
8	Nadeau 2012	Circles of Tobacco Wisdom																	1					1
9	Schinke 1996	FACETS curriculum			1		1												1					3

ID for reference		Program name (A-Z)	**Brusse 2014** JMIR (4)	**CADTH 2013** Rapid Review (0)	**Carson 2012a** CDSR (prevention) (2)	**Carson 2012b** CDSR (cessation) (4)	**Carson 2013** Respirology (6)*	**Carson 2015** Respirology (9)*	**Carson 2014** Evidence Base (91)	**Clifford 2009** Hlth Prom Int (2)	**Clifford 2011** ANZ J Pub Hlth (4)	**DiGiacomo 2011** Int J Environ Res Pub Hlth (9)	**Gould 2013a** Tobacco Control (21)	**Gould 2013b** Nicotine & Tobacco Cont (7)	**Ivers 2003** ANZ J Pub Hlth (4)	**Ivers 2011** Closing the Gap (11)**	**Ivers 2014** NSW framework (11)**	**Johnston 2013** Nicotine & Tobacco Res (5)	**Minicheiello 2016** BMC Public Health (85)***	**Passey 2013** MJA (2)	**Power 2009** Hlth Prom J Aus (14)	**Thompson 2011** ANZ J Pub Hlth (11)	**Upton 2014** Rapid Review DoH (31)**	No. of reviews including this study
			**Reviews** (number of references to included studies)																					
10	Johnson 1997	GAINS: Giving American Indians No-smoking Strategies Project							1										1					2
11	Weaver 2010	Healthy Living in Two Worlds																	1					1
12	Dixon 2007	Keepin it R.E.A.L. (Refuse, Explain, Avoid and Leave)							1															1
13	Montgomery 2012	Native Comic Book Project																	1					1
14	Beckham 2007	Not reported in review																	1					1
15	Bonevski 2011	Not reported in review							1															1
16	Doorenbos 2011	Not reported in review											1											1
17	Gilchrist 1987	Not reported in review			1		1		1															3
18	Hensel 1995	Not reported in review							1			1							1					3
19	Mitschke 2010	Not reported in review											1											1
20	Moncher 1994	Not reported in review							1										1					2
21	Patten 2013	Not reported in review																	1					1
22	Patten 2014	Not reported in review																	1					1
23	Schinke 2000	Not reported in review																	1					1
24	Smith 2014	Not reported in review							1															1
25	Swartz 2006	Not reported in review											1											1
26	Taualii 2010	Not reported in review											1											1
27	Vogeltanz-Holm 2009	Not reported in review											1											1
28	Weaver 1999	Not reported in review																	1					1
29	Davis 1999	Pathways to Health							1															1
30	Santos 2008	PAU protocol																	1					1
31	Maher 2007	Quitline Washington State										1												1
32	Richards 2012	Sacred Beginnings Project																	1					1
33	Choi 2006	Second wind											1											1

			**Reviews** (number of references to included studies)																					
**ID for reference**		**Program name (A-Z)**	**Brusse 2014** JMIR (4)	**CADTH 2013** Rapid Review (0)	**Carson 2012a** CDSR (prevention) (2)	**Carson 2012b** CDSR (cessation) (4)	**Carson 2013** Respirology (6)*	**Carson 2015** Respirology (9)*	**Carson 2014** Evidence Base (91)	**Clifford 2009** Hlth Prom Int (2)	**Clifford 2011** ANZ J Pub Hlth (4)	**DiGiacomo 2011** Int J Environ Res Pub Hlth (9)	**Gould 2013a** Tobacco Control (21)	**Gould 2013b** Nicotine & Tobacco Cont (7)	**Ivers 2003** ANZ J Pub Hlth (4)	**Ivers 2011** Closing the Gap (11)**	**Ivers 2014** NSW framework (11)**	**Johnston 2013** Nicotine & Tobacco Res (5)	**Minicheiello 2016** BMC Public Health (85)***	**Passey 2013** MJA (2)	**Power 2009** Hlth Prom J Aus (14)	**Thompson 2011** ANZ J Pub Hlth (11)	**Upton 2014** Rapid Review DoH (31)**	No. of reviews including this study
34	Daley 2006	Second wind											1											1
35	Bowen2012	SmokingZine website																	1					1
36	Farmer 2014	Sonoma County Indian Health Project (SCIHP)																	1					1
37	Fenn 2007	Southcentral Foundation Tobacco Cessation Initiative																	1					1
38	Davis 1995	Southwest Cardiovascular Curriculum							1															1
39	Schinke 1994	The Boy and Woman Bear																	1					1
40	Johnson 2009	Think Smart																	1					1
41	Witmer 2004	Traditions of the heart																	1					1
42	Pacheco 2014	Web based smoking cessation program (including All Nations Breath of Life)							1															1
43	Bosma 2014	Wiidookowishin (Help Me) program																	1					1
44	D'Silva 2011	Wiidookowishin (Help Me) program							1										1					2

*The two Cochrane reviews by Carson on smoking prevention [27, 28]; on smoking cessation [33, 34] appear to have been updated but, at the time of the search for this overview, were reported as conference abstracts only. Additional studies were included in these reviews (taking the total number of studies to N=6 and N=9 respectively) but no information was reported in the abstract to enable the studies to be identified. Hence the matrix shows only the original number of studies and these reviews were not included when calculating the mean number of references to included studies per review.

** Number based on references in text; unable to confirm this covers all references for included studies. [18,19; 20]

*** Includes all references to included studies, irrespective of quality (only moderate and strong quality studies were included in the synthesis; weak studies were excluded) [32]

† This reference reports evaluations for multiple programs, hence has more than one entry in this table.

References to studies included in reviews included in this Overview

Studies in Australia

1. Finlay S: Aboriginal tobacco resistance tool kit, CEITC Indigenous Projects Register, viewed 29 September 2014, <www.ceitc.org.au/aboriginal-tobacco-resistance-tool-kit>. 2013.
2. Maddox R, Davey R, Cochrane T, Lovett R, van der Sterren A: Study protocol--Indigenous Australian social networks and the impact on smoking policy and programs in Australia: protocol for a mixed-method prospective study. BMC public health 2013, 13:879.
3. Tasmanian Aboriginal Centre Inc: Alcohol, tobacco and other drugs program (Tasmanian Aboriginal Centre), Programs and Projects, viewed 29 September 2014, <www.healthinfonet.ecu.edu.au/key-resources/programs-projects?pid=2352>. 2014.
4. Shah S, Saunders J, Chang A, Gibson P, McGee J, McCallum G: Asthma and smoking prevention project, viewed 29 September 2014, <www.menzies.edu.au/page/Research/Projects /Lungs/ASAP_-_Anti-Smoking_Asthma_Program_study>. 2013.
5. Lewis M: Beyond the big smoke, CEITC Indigenous Projects Register, viewed 29 September 2014, www.ceitc.org.au/beyond-big-smoke. 2006.
6. Australian Government Department of Health: Indigenous Anti-smoking campaign: Break the Chain. <www.quitnow.gov.au/internet/quitnow/publishing.nsf/Content/btc-indsurvey-execsumm. 2011.
7. Boyle T, Shepherd CCJ, Pearson G, Monteiro H, McAullay D, Economo K: Awareness and impact of the ‘Bubblewrap’ advertising campaign among Aboriginal smokers in Western Australia. Tob Control 2010, 19.
8. Cancer Council Western Australia: Bubblewrap campaign summary. http://www.cancerwa.asn.au/resources/2009-MSH-Wave-19-2008-Bubblewrap-Campaign-Summary.pdf. 2008.
9. Davison J: ‘Butt busters’ program, CEITC Indigenous Projects Register, viewed 29 September 2014, <www.ceitc.org.au/butt-busters-program>. 2005.
10. "Young D, Campbell S: Butt Out: Nicotine replacement therapy trial. In: Centre for Excellence in Indigenous Tobacco Control, National Indigenous Tobacco Control Research Workshop Report. Melbourne, Australia: Koorie Heritage Trust Inc; 2007.
11. Drug and Alcohol Services South Australia: Ceduna Day Centre, viewed 29 September 2014, <www.healthinfonet.ecu.edu.au/key-resources/programs-projects?pid=557>. 2014.
12. Sarin J, Graham F, Walker M: Clean air dreaming, CEITC Indigenous Projects Register, viewed 29 September 2014, <www.ceitc.org.au/clean-air-dreaming>. 2007.
13. Clifford A, Parker R: Deadly Choices campaign evaluation: Final Report. Independent Evaluation undertaken by ‘Pollinate’ on behalf of IUIH. 2014.
14. Institute for Urban Indigenous Health: Deadly Choices Research and Evaluation. Bowen Hills; 2014.
15. Malseed C, Nelson A, Ware R, Lacey I, Lander K: Deadly Choices™ community health events: a health promotion initiative for urban Aboriginal and Torres Strait Islander people. Aust J Prim Health 2014, 20.
16. Malseed C, Nelson, A. and Ware, R.: Evaluation of a School-Based Health Education Program for Urban Indigenous Young People in Australia. Health 2014, 6:587-597.
17. Malseed C: Deadly choices health promotion initiative evaluation report January 1 -December 31, 2013: Institute for Urban Indigenous Health 2013.
18. Day G: Deadly Nungas say no to puiya! project. In: Oceania Tobacco Control Conference. Auckland, New Zealand; 2007.
19. Kickett D: Drug & alcohol awareness, CEITC Indigenous Projects Register, viewed 29 September 2014, <www.ceitc.org.au/drug-alcohol-awareness>. 2009.
20. Minniecon S: Engaging an Aboriginal Elder in promoting tobacco control messages to the Aboriginal & Torres Strait Islander community project, CEITC Indigenous Projects Register,

viewed 29 September 2014, <www.ceitc.org.au/engaging-aboriginal-elder-promoting-tobacco-control-messages-aboriginal-torres-strait-islander-commu>. 2005.
21. Cancer Council South Australia: Greater Aboriginal support from the NT Quitline, viewed 29 September 2014, <www.cancersa.org.au/stay-up-to-date/press-releases/details/greater-aboriginal-support-from-the-nt-quitline>. 2014.
22. Gould G, McGechan A, van der Zwan R: Give up the smokes: a smoking cessation program for Indigenous Australians, viewed 25 August 2014, <www.ruralhealth.org.au/10thNRHC/10thnrhc.ruralhealth.org.au/papers/docs/Gould_Gillian_D9.pdf>. In: 10th National Rural Health Conference. 2009.
23. Gould G: Give up the smokes, CEITC Indigenous Projects Register, viewed 29 September 2014, <www.ceitc.org.au/give-smokes>. 2006.
24. Dean K: Gnumaries hurt program, CEITC Indigenous Projects Register, viewed 29 September 2014, <www.ceitc.org.au/gnumaries-hurt-program>. 2010.
25. Carson K: Improving health for Aboriginal people through tobacco related research, (NCT01735448), viewed 30 September 2014, <clinicaltrials.gov/show/NCT01735448>. 2012.
26. Bailie R, Griffin J, Kelaher M, McNeair T, Percival N, Laycock A, Schierhout G: Sentinel Sites Evaluation: Final Report prepared for the Australian Government Department of Health and Ageing. In. Canberra: Menzies School of Health Research; 2013.
27. KPMG: Monitoring and Evaluation of the Indigenous Chronic Disease Package Local Community Campaign (A3) measure. Final report prepared for the Australian Government Department of Health and Ageing. In.: KPMG; 2013.
28. KPMG: Monitoring and Evaluation of the Indigenous Chronic Disease Package. Final Report prepared for the Australian Government Department of Health and Ageing. In., vol. 1: KPMG; 2013.
29. Cargo M, Marks E, Brimblecombe J, Scarlett M, Maypilama E, Dhurrkay JG, Daniel M: Integrating an ecological approach into an Aboriginal community-based chronic disease prevention program: a longitudinal process evaluation. BMC public health 2011, 11:299.
30. Larson A: Indigenous Healthy Lifestyle Program evaluation final report. Office of Aboriginal Health. In. Geraldton, W.A Combined Universities Centre for Rural Health; 2010.
31. Harvey D, Tsey K, Cadet-James Y, Minniecon D, Ivers R, McCalman J: An evaluation of tobacco brief intervention training in three indigenous health care settings in north Queensland. Aust N Z J Public Health 2002, 26.
32. Murphy A: Indigenous women's project, CEITC Indigenous Projects Register, viewed 29 September 2014, <www.ceitc.org.au/indigenous-womens-project>. 2009.
33. Hippi W: I-Quitt, CEITC Indigenous Projects Register, viewed 29 September 2014, <www.ceitc.org.au/i-quitt>. 2009.
34. Griffiths M: Justice health quit smoking project, CEITC Indigenous Projects Register, viewed 29 September 2014, <www.ceitc.org.au/justice-health-quit-smoking-project>. 2009.
35. Martinez A: Keep Koori kids smoke free, CEITC Indigenous Projects Register, viewed 29 September 2014, <www.ceitc.org.au/keep-koori-kids-smoke-free>. 2013.
36. Aboriginal Health and Medical Research Council of New South Wales: Kick the habit social marketing campaign, Programs, viewed 29 September 2014 <www.ahmrc.org.au/index.php?option=com_content& view=article&id=240:tobacco-resistance-and-control&catid=14:programs&Itemid=14>. 2010.
37. Adams K, Briggs V: Galnya Angin (good air) Partnerships in Indigenous Tobacco Control: Centre for Excellence in Indigenous Tobacco Control; 2005.
38. Mark A, McLeod I, Booker J, Ardler C: Aboriginal Health Worker Smoking: A Barrier to Lower Community Smoking Rates? Aboriginal and Islander Health Worker Journal 2005, 29(5):[22]-[26].
39. Mark A, McLeod I, Booker J, Ardler C: The Koori Tobacco Cessation Project. Health Promotion Journal of Australia 2004, 15(3):200-204.

40. Chapman L: Make smoking history, CEITC Indigenous Projects Register, viewed 29 September 2014, <www.ceitc.org.au/make-smoking-history>. 2000.
41. Burgess P, McDonald J, Djabibba S, Namunurki S, Magaldagi L, Connors C, Matthews H, Thomas D: Smokebusters: Maningrida's experience implementing a tobacco control program. The Chronicle 2008, 11(4):8-10.
42. Johnston F, Beecham R, Dalgleish P, Malpraburr T, Gamarania G: The Maningrida "Be Smoke Free" project. Health promotion journal of Australia : official journal of Australian Association of Health Promotion Professionals 1998, 8.
43. Thomas D: Monitoring and evaluating Aboriginal tobacco control CEITC Indigenous Projects Register, viewed 29 September 2014, <www.ceitc.org.au/monitoring-evaluating-aboriginal-tobacco-control>. 2007.
44. Institute for Urban Indigenous Health: Murri places, smoke-free spaces, CEITC Indigenous Projects Register, viewed 29 September 2014, www.ceitc.org.au/murri-places-smoke-free-spaces. 2011.
45. Dimer L: My heart my family our culture, CEITC Indigenous Projects Register, viewed 29 September 2014, <www.ceitc.org.au/my-heart-my-family-our-culture>. 2004.
46. Ell P, Abel M, Pedic F: National Tobacco Campaign Formative Research. In.: GfK Australia; 2013.
47. National Tobacco Campaign: Australia's National Tobacco Campaign: Evaluation Report, Volume 1. In. Canberra: Commonwealth Department of Health and Aged Care; 1999: 252.
48. ORIMA: Evaluation of Burst 2 of the Quit for You Quit for Two campaign. Retrieved from the Quitline website: http://quitnow.gov.au/internet/quitnow/publishing.nsf/Content/5235148D288A12FDCA257AA800149446/$File/NTC-MTA_Evaluation%20of%20Burst%202%20of%20QFYQF2%20-%20Final%20Report.docx. 2013.
49. Adams K, Briggs V: Galnya Angin (good air) Partnerships in Indigenous Tobacco Control: Centre for Excellence in Indigenous Tobacco Control; 2005.
50. Webb C: No more boondah, CEITC Indigenous Projects Register, viewed 29 September 2014, <www.ceitc.org.au/no-more-boondah>. 2012.
51. Bell J: No smokes project - summative evaluation report: findings from qualitative focus groups: Menzies School of Health Research; 2012.
52. Gould G: No smokes north coast, CEITC Indigenous Projects Register, viewed 29 September 2014, <www.ceitc.org.au/no-smokes-north-coast>. 2010.
53. Adams K, Walker H: Smokes and Aboriginal Health Worker Training. Aboriginal and Islander Health Worker Journal 2006, 30(2):15-16, 21.
54. Andrews B, Oates F, Naden P: Smoking among aboriginal health workers: findings of a 1995 survey in western New South Wales. Aust N Z J Public Health 1997, 21(7):789-791.
55. Bardsley P, Olekalns N: The impact of anti-smoking policies on tobacco consumption in Australia. Health Promotion Journal of Australia 1999, 9(3):202-205.
56. DiGiacomo M, Davidson PM, Davison J, Moore L, Abbott P: Stressful life events, resources, and access: key considerations in quitting smoking at an Aboriginal Medical Service. Aust New Zeal J Publ Health 2007, 31.
57. Douglas TA, Buettner PG, Whitehall J: Maternal awareness of sudden infant death syndrome in North Queensland, Australia: an analysis of infant care practices. J Paediatr Child Health 2001, 37(5):441-445.
58. Eades SJ, Sanson-Fisher RW, Wenitong M, Panaretto K, D'este C, Gilligan C: An intensive smoking intervention for pregnant Aboriginal and Torres Strait Islander women: a randomised controlled trial. Med J Aust 2012, 197.
59. Fairer Health Victoria: Case studies on improving health for all. Women's Health Goulburn North East website: http://www.whealth.com.au/documents/health/kwhd_fairer_health.pdf. 2009.

60. Fletcher G, Fredericks B, Adams K, Finlay S, Andy S, Briggs L, Hall R: Having a yarn about smoking: using action research to develop a 'no smoking' policy within an Aboriginal Health Organisation. Health Policy 2011, 103(1):92-97.
61. Gilligan C, Sanson-Fisher RW, D'Este C, Eades S, Wenitong M: Knowledge and attitudes regarding smoking during pregnancy among Aboriginal and Torres Strait Islander women. Med J Aust 2009, 190(10):557-561.
62. Gilligan C: Aboriginal and Torres Strait Islander women: An examination of smoking during pregnancy: University of Newcastle; 2008.
63. Gray D, Sputore B, Walker J: Evaluation of an Aboriginal Health Promotion Program: A Case Study from Karalundi. In: Indigenous Australian Alcohol and Other Drug Issues: Research from the National Drug Research Institute. Edited by Gray D, Saggers S. Perth: National Drug Research Institute, Curtin University of Technology; 2002.
64. Heath DL, Panaretto K, Manessis V, Larkins S, Malouf P, Reilly E, Elston J: Factors to Consider in Smoking Interventions for Indigenous Women. Australian Journal of Primary Health 2006, 12(2):131-136.
65. Ivers RG, Farrington M, Burns CB, Bailie RS, D'Abbs PH, Richmond RL: A study of the use of free nicotine patches by Indigenous people. Aust N Z J Public Health 2003, 27.
66. Johnston V, Thomas DP: Smoking behaviours in a remote Australian Indigenous community: the influence of family and other factors. Soc Sci Med 2008, 67(11):1708-1716.
67. Johnston V, Thomas DP: What works in Indigenous tobacco control? The perceptions of remote Indigenous community members and health staff. Health promotion journal of Australia : official journal of Australian Association of Health Promotion Professionals 2010, 21(1):45-50.
68. Lindorff K: Tobacco: Time for Action. In. Canberra (AUST): National Aboriginal Community Controlled Health Organisation; 2002.
69. Panaretto K, Coutts J, Johnson L, Morgan A, Leon D, Hayman N: Evaluating performance of and organisational capacity to deliver brief interventions in Aboriginal and Torres Strait Islander medical services. Aust N Z J Public Health 2010, 34(1):38-44.
70. Pilkington AAG, Carter OBJ, Cameron AS, Thompson SC: Tobacco control practices among Aboriginal health professionals in Western Australia. Australian Journal of Primary Health 2009, 15(2):152-158.
71. Stewart HS, Bowden JA, Bayly MC, Sharplin GR, Durkin SJ, Miller CL, Givans SE, Warne CD, Wakefield MA: Potential effectiveness of specific anti-smoking mass media advertisements among Australian Indigenous smokers. Health Education Research 2011.
72. Thomas DP, Ferguson M, Johnston V, Brimblecombe J: Impact and perceptions of tobacco tax increase in remote Australian Aboriginal communities. Nicotine & tobacco research : official journal of the Society for Research on Nicotine and Tobacco 2013, 15.
73. Wakefield MA, Durkin S, Spittal MJ, Siahpush M, Scollo M, Simpson JA, Chapman S, White V, Hill D: Impact of Tobacco Control Policies and Mass Media Campaigns on Monthly Adult Smoking Prevalence. American Journal of Public Health 2008, 98(8):1443-1450.
74. West L, Young D, Lloyd J: Far North Queensland Aboriginal and Torres Strait Islander Health Workers; Knowledge, Attitudes & Beliefs about Smoking Cessation and Prevention. In. Brisbane (AUST); 1998.
75. Wilson G: What do Aboriginal women think is good antenatal care? Consultation report. Alice Springs, NT: Cooperative Research Centre for Aboriginal Health. Retrieved from www.lowitja.org.au/lowitja-publishing/C015. 2009.
76. Wood L, France K, Hunt K, Eades S, Slack-Smith L: Indigenous women and smoking during pregnancy: knowledge, cultural contexts and barriers to cessation. Soc Sci Med 2008, 66(11):2378-2389.
77. Gussy K: Our space smoke free, CEITC Indigenous Projects Register, viewed 29 September 2014, <www.ceitc.org.au/our-space-smoke-free>. 2010.

78. Gould GS, McEwen A: An intensive smoking intervention for pregnant Aboriginal and Torres Strait Islander women: a randomized controlled trial. Med J Aust 2012, 197.
79. Aboriginal Health and Medical Research Council and Cancer Institute NSW: Qualitative Research Report- Quitline Enhancement Project. 2011.
80. Ford V: Reducing the risk of SIDS in Aboriginal communities, CEITC Indigenous Projects Register, viewed 29 September 2014, <www.ceitc.org.au/reducing-risk-sids-aboriginal-communities>. 2005.
81. Chamberlain C: Reducing smoking amongst pregnant Aboriginal women in Victoria: an holistic approach, CEITC Indigenous Projects Register, viewed 29 September 2014, <www.ceitc.org.au/reducing-smoking-amongst-pregnant-aboriginal-women-victoria-holistic-approach>. 2008.
82. Coole J, Schultz A: Regional tackling smoking and healthy lifestyle workforce and activities, CEITC Indigenous Projects Register, viewed 29 September 2014, <www.ceitc.org.au/regional-tackling-smoking-and-healthy-lifestyle-workforce-and-activities>. 2010.
83. Gentle I: Remote Aboriginal tobacco project, CEITC Indigenous Projects Register, viewed 29 September 2014, <www.ceitc.org.au/remote-aboriginal-tobacco-project>. 2008.
84. Nunkuwarrin Yunti of South Australia Inc: Rewrite your story, viewed 29 September 2014, <www.rewriteyourstory.com.au/about-us/the-campaign>. 2013.
85. Yarran C: Rockingham and Kwinana tobacco control project, CEITC Indigenous Projects Register, viewed 29 September 2014, <www.ceitc.org.au/rockingham-and-kwinana-tobacco-control-project>. 2010.
86. Adams K, Briggs V: Galnya Angin (good air) Partnerships in Indigenous Tobacco Control: Centre for Excellence in Indigenous Tobacco Control; 2005.
87. Adams K, Rumbiolo D, Charles S: Evaluation of Rumbalara's 'No More Dhonga' Short Course in Giving Up Smokes. Aboriginal and Islander Health Worker Journal 2006, 30(5):20-21.
88. Rumbalara Aboriginal Co-operative: Rumbalara quit program, CEITC Indigenous Projects Register, viewed 29 September 2014, <www.ceitc.org.au/rumbalara-quit-program>. 2012.
89. Healthway Western Australian State Government: The ‘Say No to Smokes’ project - Success stories campaign (WA), CEITC Indigenous Projects Register, viewed 29 September 2014, <www.ceitc.org.au/say-no-smokes-project-success-stories-campaign-wa>. 2002.
90. Bond C: Smoke free life research project, CEITC Indigenous Projects Register, viewed 29 September 2014, <www.ceitc.org.au/smoke-free-life-research-project>. 2012.
91. Carroll B: NSW SmokeCheck project, CEITC Indigenous Projects Register, viewed 29 September 2014, <www.ceitc.org.au/nsw-smokecheck-project>. 2006.
92. Hearn S, Nancarrow H, Rose M, Massi L, Wise M, Conigrave K, Barnes I, Bauman A: Evaluating NSW SmokeCheck: a culturally specific smoking cessation training program for health professionals working in Aboriginal health. Health promotion journal of Australia : official journal of Australian Association of Health Promotion Professionals 2011, 22(3):189-195.
93. Jenkinson K: Smoke Check NT, CEITC Indigenous Projects Register, viewed 29 September 2014, <www.ceitc.org.au/smoke-check-nt>. 2007.
94. Queensland Health: SmokeCheck Evaluation Report 2006. In. Brisbane (AUST): State Government of Queensland; 2007.
95. Campbell S, Bohanna I, McKeown-Young D, Esterman A, Cadet-James Y, McDermott R: Evaluation of a community-based tobacco control intervention in five remote north Queensland Indigenous communities. Int J Health Promot Educ 2014, 52.
96. Aboriginal Health Council of SA: 'Puya Wiya' [No Smoking] 'Be Wise, Give Up': South Australian Aboriginal Tobacco Control Project. In. Adelaide (AUST): Aboriginal Health Council of SA,; 2002.
97. Stewart H: Puyu Wiya Smokecheck, CEITC Indigenous Projects Register, viewed 29 September 2014, <www.ceitc.org.au/puyu-wiya-smokecheck>. 2011.

98. Quit SA: Smoke-free pregnancy project - Aboriginal women and their families, CEITC Indigenous Projects Register, viewed 29 September 2014, <www.ceitc.org.au/smoke-free-pregnancy-project-aboriginal-women-and-their-families>. 2011.
99. Lynch T: Smokers program, CEITC Indigenous Projects Register, viewed 29 September 2014, <www.ceitc.org.au/smokers-program>. 2005.
100. Ryan P: Smoking no good aye, CEITC Indigenous Projects Register, viewed 29 September 2014, <www.ceitc.org.au/smoking-no-good-aye>. 2010.
101. Daniel M: Smoking reduction strategy development and intervention among Aboriginal health workers, CEITC Indigenous Projects Register, viewed 29 September 2014, <www.ceitc.org.au/smoking-reduction-strategy-development-and-intervention-among-aboriginal-health-workers>. 2008.
102. Cosh S, Maksimovic L, Ettridge K, Copley D, Bowden JA: Aboriginal and Torres Strait Islander utilization of the Quitline service for smoking cessation in South Australia. Aust J Prim Health 2013, 19.
103. Johnston V, Thomas D, Westphal D, Earnshaw C: Starting to smoke: experiences of Indigenous youth, viewed 31 August 2014, <www.lowitja.org.au/sites/default/files/docs/Starting%20to%20Smoke-FINALweb.pdf>. 2013.
104. Passey M, Gale J, Holt B, Leatherday C, Roberts C, Kay D, Rogers L, Paden V: Stop smoking in its tracks: understanding smoking by rural Aboriginal women, viewed 29 September 2014, <www.ruralhealth.org.au/10thNRHC/10thnrhc.ruralhealth.org.au/papers/docs/Passey_Megan_D9.pdf>. In: 10th National Rural Health Conference. Cairns, Australia; 2009.
105. Passey M: Supporting mums to quit: smoking intervention research for pregnant rural Aboriginal women. CEITC Indigenous Projects Register, viewed 29 September 2014, <www.ceitc.org.au/supporting-mums-quit-smoking-intervention-research-pregnant-rural-aboriginal-women>. 2009.
106. Thomas D: Talking about the Smokes: preliminary findings from baseline survey. 2014.
107. Boadle M: Tasmanian Aboriginal tobacco control project, CEITC Indigenous Projects Register, viewed 29 September 2014, <www.ceitc.org.au/tasmanian-aboriginal-tobacco-control-project>. 2006.
108. Marley JV, Atkinson D, Kitaura T, Nelson C, Gray D, Metcalf S: The Be Our Ally Beat Smoking (BOABS) study, a randomised controlled trial of an intensive smoking cessation intervention in a remote Aboriginal Australian health care setting. BMC public health 2014, 14.
109. Marley JV, Kitaura T, Atkinson D, Metcalf S, Maguire GP, Gray D: Clinical trials in a remote Aboriginal setting: lessons from the BOABS smoking cessation study. BMC public health 2014, 14.
110. Ivers R, Castro A, Parfitt D, Bailie R, D'Abbs P, Richmond R: The tobacco project. Cooperative Research Centre for Aboriginal Health. https://www.lowitja.org.au/sites/default/files/docs/The_Tobacco_Project.pdf 2005.
111. Ivers RG, Castro A, Parfitt D, Bailie RS, D’Abbs PH, Richmond RL: Evaluation of a multi-component community tobacco intervention in three remote Australian Aboriginal communities. Aust N Z J Public Health 2006, 30.
112. Ivers R, Castro A, Parfitt D, Bailie RS, Richmond RL, D’Abbs PH: Television and delivery of health promotion programs to remote Aboriginal communities. Health promotion journal of Australia : official journal of Australian Association of Health Promotion Professionals 2005, 16.
113. Ivers RG, Castro A, Parfitt D, Bailie RS, Richmond RL, D’abbs PH: The role of remote community stores in reducing the harm resulting from tobacco to aboriginal people. Drug Alcohol Rev 2006, 25.
114. Thomas D, Johnston V, Fitz J: Lessons for Aboriginal tobacco control in remote communities: an evaluation of the Northern Territory ‘Tobacco Project’. Aust N Z J Public Health 2010, 34.

115. Walley C, Sullivan D: The Western Australian Aboriginal smoking project. Health Promotion Journal of Australia 1998, 8:55-58.
116. Seibold M: Indigenous Tobacco Control Pilot Project: Process Evaluation, Workplace Policy Development Pilot, Phase One. Brisbane: Queensland Health. 2000.
117. Robertson J, Pointing BS, Stevenson L, Clough AR: We made the rule, we have to stick to it: towards effective management of environmental tobacco smoke in remote Australian Aboriginal communities. Int J Environ Res Public Health 2013, 10.
118. Robertson J: Top end tobacco project, CEITC Indigenous Projects Register, viewed 29 September 2014, <www.ceitc.org.au/top-end-tobacco-project>. 2007.
119. Robertson J: Sustainable Interventions to Address High Rates of Smoking Among Indigenous People in the Northern Territory's 'Top End': Project summary. In. Cairns (AUST): James Cook University; 2007.
120. Government of Victoria: Closing the Gap in Aboriginal Health Outcomes Initiative: Final Evaluation Report: URBIS; 2014.
121. Read V: WA prisons smoking reduction plan, CEITC Indigenous Projects Register, viewed 29 September 2014, www.ceitc.org.au/wa-prisons-smoking-reduction-plan>. 2012.
122. Davis M: Yarning it up, CEITC Indigenous Projects Register, viewed 29 September 2014, <www.ceitc.org.au/yarning-it>. 2013.
123. Ramamoorthi R: Healthy Starts (Te Piripohotanga), CEITC Indigenous Projects Register, viewed 29 September 2014, <www.ceitc.org.au/healthy-starts-te-piripohotanga>. 2009.
124. Walker N, Johnston V, Glover M, Bullen C, Trenholme A, Chang A: Effect of a family-centered, secondhand smoke intervention to reduce respiratory illness in Indigenous infants in Australia and New Zealand: A randomized controlled trial. Nicotine & tobacco research : official journal of the Society for Research on Nicotine and Tobacco 2015, 17.

Studies in Canada

1. Mashford-Pringle A: Early Learning for Aboriginal Children: Past, Present and Future and an Exploration of the Aboriginal Head Start Urban and Northern Communities Program in Ontario. First Peoples Child & Family Review 2012, 7.
2. Mashford-Pringle A: The impacts on health and education for children and families enrolled in Aboriginal Head Start Urban and Northern Communities in Ontario. University of Toronto; 2008.
3. Mitchell S: Tobacco cessation strategies for First Nations, Inuit and Metis: an environmental scan and annotated bibliography: National Collaborating Centre for Aboriginal Health; 2007.
4. Aboriginal Cancer Care Unit: A case study approach: lessons learned in Ontario – Aboriginal tobacco cessation. Cancer Care Ontario. 2008.
5. Inuit Tapiriit Kanatami: Blue Light Campaign Data Comparison. Government of Nunavut; 2011.
6. Hayward LM, Campbell HS, Sutherland-Brown C: Aboriginal users of Canadian quitlines: an exploratory analysis. Tob Control 2007, 16.
7. Wesche S, Ryan R, Carry C: First Nations, Inuit and Métis: respiratory health initiatives environmental scan: National Aboriginal Health Organization; 2011.
8. Baydala L, Sewlal B, Rasmussen C, Alexis K, Fletcher F, Letendre L, Odishaw J, Kennedy M, Kootenay B: A culturally adapted drug and alcohol abuse prevention program for Aboriginal children and youth. Progress in Community Health Partnerships: Research, education and action 2009, 3(1):37-46.
9. Irfan S, Schwartz R: Youth engagement and tobacco control in on-reserve Aboriginal communities: Ontario Tobacco Research Unit; 2012.
10. Wesche S, Ryan R, Carry C: First Nations, Inuit and Métis: respiratory health initiatives environmental scan: National Aboriginal Health Organization; 2011.

11. McKennitt DW, Currie CL: Does a culturally sensitive smoking prevention program reduce smoking intentions among Aboriginal children? A pilot study. Am Indian Alsk Native Ment Health Res 2012, 19(2):55-63.
12. Wesche S, Ryan R, Carry C: First Nations, Inuit and Métis: respiratory health initiatives environmental scan: National Aboriginal Health Organization; 2011.
13. Aboriginal Cancer Care Unit: A case study approach: lessons learned in Ontario – Aboriginal tobacco cessation. Cancer Care Ontario. 2008.
14. Chansonneuve D: Addictive behaviours among Aboriginal People in Canada: Aboriginal Healing Foundations; 2007.
15. Inuit Tobacco Free Network: Smoke stories: quit clips by Inuit youth video screening contest report Nunavut classrooms: National Aboriginal Health Organization; 2011.
16. Wesche S, Ryan R, Carry C: First Nations, Inuit and Métis: respiratory health initiatives environmental scan: National Aboriginal Health Organization; 2011.
17. Irfan S, Schwartz R, Bierre S: Engaging Aboriginal youth in off-reserve communities: a case study of MAKWA: Ontario Tobacco Research Unit; 2012.

Studies in Fiji

1. Groth-Marnat G, Leslie S, Renneker M: Tobacco control in a traditional Fijian village: indigenous methods of smoking cessation and relapse prevention. Soc Sci Med 1996, 43.

Studies in New Zealand

1. Maddison R, Roberts V, McRobbie H, Bullen C, Prapavessis H, Glover M, Jiang Y, Brown P, Leung W, Taylor S et al: Exercise counseling to enhance smoking cessation outcomes: the Fit2Quit randomized controlled trial. Ann Behav Med 2014, 48(2):194-204.
2. Fernandez C, Wilson D. Maori women's views on smoking cessation initiatives. Nurs Prax N Z 2008;24:27e40.
3. Grigg M, Waa A, Bradbrook SK: Response to an indigenous smoking cessation media campaign - it's about whanau. Aust New Zeal J Publ Health 2008, 32.
4. Wilson N, Grigg M, Graham L, Cameron G: The effectiveness of television advertising campaigns on generating calls to a national Quitline by Māori. Tob Control 2005, 14.
5. Glover M, Scragg R, Nosa V, Bullen C, McCool J, Kira A: Keeping Kids Smokefree: rationale, design, and implementation of a community, school, and family-based intervention to modify behaviors related to smoking among Maori and Pacific Island children in New Zealand. Int Q Community Health Educ 2009, 30(3):205-222.
6. Wilson N, Weerasekera D, Hoek J, et al. Smoker recognition of a national quitline number following introduction of improved pack warnings: ITC Project New Zealand. Nicotine Tob Res 2010;12(Suppl 1):S72e7.
7. Bullen C, Howe C, Lin RB, Grigg M, Laugesen M, McRobbie H, Glover M, Walker N, Wallace-Bell M, Whittaker R et al: Pre-cessation nicotine replacement therapy: pragmatic randomized trial. Addiction 2010, 105(8):1474-1483.
8. Watson D, Glover M, McCool J, Bullen C, Adams B, Min S: Impact of national smoke free environments laws on teachers, schools and early childhood centres. Health promotion journal of Australia : official journal of Australian Association of Health Promotion Professionals 2011, 22.
9. Cowie N, Glover M, Gentles D: Taxing times? Smoker response to tax increases. Ethnicity and Inequalities in Health and Social Care 2014, 7.

10. Glover M, Kira A, Walker N, Bauld L: Using incentives to encourage smoking abstinence among pregnant Indigenous women? A feasibility study. Matern Child Health J 2014.
11. Holt S, Timu-Parata C, Ryder-Lewis S, Weatherall M, Beasley R: Efficacy of bupropion in the indigenous Māori population in New Zealand. Thorax 2005, 60.
12. Walker N, Howe C, Bullen C, Grigg M, Glover M, McRobbie H, Rodgers A, Whittaker R: Does improved access and greater choice of nicotine replacement therapy affect smoking cessation success? Findings from a randomized controlled trial. Addiction 2011, 106:1176–1185.
13. Whittaker R, Dorey E, Bramley D, Bullen C, Denny S, Elley CR, Maddison R, McRobbie H, Parag V, Rodgers A et al: A theory-based video messaging mobile phone intervention for smoking cessation: randomized controlled trial. J Med Internet Res 2011, 13(1):e10.
14. Whittaker R, Maddison R, McRobbie H, Bullen C, Denny S, Dorey E, et al. A multimedia mobile phone-based youth smoking cessation intervention: findings from content development and piloting studies. J Med Internet Res 2008;10(5):e49
15. Hiscock R, Pearce J, Barnett R, Moon G, Daley V: Do smoking cessation programmes influence geographical inequalities in health? An evaluation of the impact of the PEGS programme in Christchurch, New Zealand. Tob Control 2009, 18.
16. Walker N, Howe C, Bullen C, Grigg M, Glover M, McRobbie H, Laugesen M, Parag V, Whittaker R: The combined effect of very low nicotine content cigarettes, used as an adjunct to usual Quitline care (nicotine replacement therapy and behavioural support), on smoking cessation: a randomized controlled trial. Addiction 2012, 107(10):1857-1867.
17. Bramley D, Riddell T, Whittaker R, Corbett T, Lin RB, Wills M: Smoking cessation using mobile phone text messaging is as effective in Māori as non-Māori. N Z Med J 2005, 118.
18. Rodgers A, Corbett T, Bramley D, Riddell T, Wills M, Lin RB, et al. Do u smoke after txt? Results of a randomised trial of smoking cessation using mobile phone text messaging. Tob Control 2005 Aug;14(4):255-261
19. Glover M, Kira A, Gentles D, Cowie N, Paton C, Moetara W: The WERO group stop smoking competition: main outcomes of a pre- and post- study. BMC public health 2014, 14:599.

Studies in Taiwan

1. Lin MH, Huang SJ, Shih WM, Wany PY, Lin LH, Hsu HC: Effects of an anti-smoking program to prevent lung cancer among urban aboriginals in Taiwan. Asian Pac J Cancer Prev 2013, 14.

Studies in the United States of America

1. Patten CA, Windsor RA, Renner CC, Enoch C, Hochreiter A, Nevak C: Feasibility of a tobacco cessation intervention for pregnant Alaska Native women. Nicotine & tobacco research : official journal of the Society for Research on Nicotine and Tobacco 2010, 12.
2. Patten CA: Tobacco cessation intervention during pregnancy among Alaska Native women. J Cancer Educ 2012, 27.
3. Boles M, Rohde K, He H, Maher JE, Stark MJ, Fenaughty A: Effectiveness of a tobacco quitline in an indigenous population: a comparison between Alaska Native people and other first-time quitline callers who set a quit date. Int J Circumpolar Health 2009, 68.
4. Choi WS, Faseru B, Beebe LA, Greiner AK, Yeh HW, Shireman T: Culturally-tailored smoking cessation for American Indians: study protocol for a randomized controlled trial. Trials 2011, 12.
5. Daley CM, Cowan P, Nollen NL, et al. Assessing the scientific accuracy, readability, and cultural appropriateness of a culturally targeted smoking cessation program for American Indians. Health Promot Pract 2009;10:386e93.

6. Makosky Daley C, Greiner A, Nazir N, Daley S, Solomon C, Braiuca S, Smith E, Choi W: All nations breath of life: Using community-based participatory research to address health disparities in cigarette smoking among American Indians. Ethnicity and Disease 2010, 20(4):334-338.
7. Horn K, Dino G, Manzo K, McCracken L, Noerachmanto N, McGloin T: Quit and reduction rates for a pilot study of the American Indian Not On Tobacco (NOT) Program. Prev Chronic Dis 2005, 2.
8. Nadeau M, Blake N, Poupart J, Rhodes K, Forster JL: Circles of Tobacco Wisdom: learning about traditional and commercial tobacco with Native elders. Am J Prev Med 2012, 43.
9. Schinke SP, Singer B, Cole K, Contento IR: Reducing cancer risk among Native American adolescents. Prev Med 1996, 25.
10. Johnson KM, Lando HA, Schmid LS, and Solberg LI.The GAINS project: outcome of smoking cessation strategies in four urban Native American clinics. Giving American Indians no-smoking Strategies. Addict Behav 1997, 22.
11. Weaver H, Jackson K: Healthy living in two worlds: testing a wellness curriculum for urban native youth. Child Adolesc Soc Work J 2010, 27.
12. Dixon AL, Yabiku ST, Okamoto SK, Tann SS, Marsiglia FF, Kulis S, Burke AM: The efficacy of a multicultural prevention intervention among urban American Indian youth in the southwest U.S. J Prim Prev 2007, 28(6):547-568.
13. Montgomery M, Manuelito B, Nass C, Chock T, Buchwald D: The Native comic book project: native youth making comics and healthy decisions. J Cancer Educ 2012, 27.
14. Beckham S, Washburn A, Ka'aha'aina D, Bradley S: Filling the void: a multi-component, culturally adapted smoking cessation program integrating Western and non-Western therapies. Am J Health Educ 2007, 38.
15. Bonevski B, Paul C, D'Este C, Sanson-Fisher R, West R, Girgis A, Siahpush M, Carter R: RCT of a client-centred, caseworker-delivered smoking cessation intervention for a socially disadvantaged population. BMC public health 2011, 11(1):70.
16. Doorenbos AZ, Jacobsen C, Corpuz R, et al. A Randomized controlled calendar mailout to increase cancer screening among urban American Indian and Alaska Native patients. J Canc Educ 2011;26:549e54.
17. Gilchrist LD, Schinke SP, Trimble JE, Cvetkovich GT: Skills enhancement to prevent substance abuse among American Indian adolescents. Int J Addict 1987, 22(9):869-879.
18. Hensel MR, Cavanagh T, Lanier AP, Gleason T, Bouwens B, Tanttila H: Quit rates at one year follow-up of Alaska Native Medical Center Tobacco Cessation Program. Alaska Med 1995, 37.
19. Mitschke DB, Loebl K, Tatafu E Jr, et al. Using drama to prevent teen smoking: development, implementation, and evaluation of crossroads in Hawai'i. Health Promot Pract 2010;11:244e8.
20. Moncher M, Schinke S: Group intervention to prevent tobacco use among Native American youth. Res Soc Work Pract 1994, 4.
21. Patten CA, Fadahunsi O, Hanza M, Smith CM, Hughes CA, Brockman TA: Development of a tobacco cessation intervention for Alaska Native youth. Addiction Res Theor 2013, 21.
22. Patten CA, Fadahunsi O, Hanza MM, Smith CA, Decker PA, Boyer R: Tobacco cessation treatment for Alaska Native adolescents: group randomized pilot trial. Nicotine & tobacco research : official journal of the Society for Research on Nicotine and Tobacco 2014, 16.
23. Schinke SP, Tepavac L, Cole KC: Preventing substance use among Native American youth: three-year results. Addict Behav 2000, 25.
24. Smith SS, Rouse LM, Caskey M, Fossum J, Strickland R, Culhane JK, Waukau J: Culturally-Tailored Smoking Cessation for Adult American Indian Smokers: A Clinical Trial. Couns Psychol 2014, 42(6):852-886.
25. Swartz LH, Noell JW, Schroeder SW, et al. A randomised control study of a fully automated internet based smoking cessation programme. Tob Control 2006;15:7e12.
26. Taualii M, Bush N, Bowen DJ, et al. Adaptation of a smoking cessation and prevention website for urban American Indian/Alaska Native youth. J Cancer Educ 2010;25:23e31.

27. Vogeltanz-Holm N, Holm J, White Plume J, et al. Confirmed recall and perceived effectiveness of tobacco counter marketing media in rural youth. Prev Sci 2009;10:325e34.
28. Weaver HN: Health concerns for Native American youth: a culturally grounded approach to health promotion. J Hum Behav Soc Environ 1999, 2.
29. Davis S, Cunningham-Sabo L: "Pathways to Health": A school-based cancer prevention project for Southwestern native American youth. In: Preventing and controlling cancer in North America: A cross-cultural perspective. Edited by Weiner D: Greenwood Publishing Group; 1999: 85-94.
30. Santos LA, Braun KL, Ae'a K, Shearer L: Institutionalizing a comprehensive tobacco-cessation protocol in an indigenous health system: lessons learned. Prog Community Health Partnersh 2008, 2.
31. Maher JE, Rohde K, Dent CW, Stark MJ, Pizacani B, Boysun MJ, Dilley JA, Yepassis-Zembrou PL: Is a statewide tobacco quitline an appropriate service for specific populations? Tob Control 2007, 16 Suppl 1:i65-70.
32. Richards J, Mousseau A: Community-based participatory research to improve preconception health among Northern Plains American Indian adolescent women. Am Indian Alsk Native Ment Health Res 2012, 19.
33. Choi WS, Daley CM, James A, et al. Beliefs and attitudes regarding smoking cessation among American Indians: a pilot study. Ethn Dis 2006;16:35e40.
34. Daley CM, James A, Barnoskie RS, et al. "Tobacco has a purpose, not just a past": feasibility of developing a culturally appropriate smoking cessation program for a pantribal native population. Med Anthropol Q 2006;20:421e40.
35. Bowen DJ, Nez Henderson P, Harvill J, Buchwald D: Short-term effects of a smoking prevention website in American Indian youth. J Med Internet Res 2012, 14.
36. Farmer J, Whitemore A, Cooper C: Health System Changes Lead to Educating and Referring More Smokers to Cessation Services. Sonoma County Indian Health Project; 2014.
37. Fenn DC, Beiergrohslein M, Ambrosio J: Southcentral Foundation tobacco cessation initiative. Int J Circumpolar Health 2007, 66.
38. Davis S, Lambert L, Gomez Y, Skipper B: Southwest cardiovascular curriculum project: study findings for American Indian elementary students. Journal of Health Education 1995, 26(2):S72-81.
39. Schinke SP, Moncher MS, Singer BR: Native American youths and cancer risk reduction. Effects of software intervention. J Adolesc Health 1994, 15.
40. Johnson KW, Shamblen SR, Ogilvie KA, Collins D, Saylor B: Preventing youths™ use of inhalants and other harmful legal products in frontier Alaskan communities: a randomized trial. Prev Sci 2009, 10.
41. Witmer JM, Hensel MR, Holck PS, Ammerman AS, Will JC: Heart disease prevention for Alaska Native women: a review of pilot study findings. J Womens Health 2004, 13.
42. Pacheco J: Web-based smoking cessation program for tribal college students. In. ClinicalTrials.gov; 2014.
43. Bosma LM, D'Silva J, Jansen A, Sandman NR, Hink RL: The Wiidookowishin program: results from a qualitative process evaluation of a culturally tailored commercial tobacco cessation program. Am Indian Alsk Native Ment Health Res 2014, 21.
44. D'Silva J, Schillo BA, Sandman NR, Leonard TL, Boyle RG: Evaluation of a tailored approach for tobacco dependence treatment for American Indians. Am J Health Promot 2011, 25.
